# SARS-CoV-2 Infects Human Pluripotent Stem Cell-Derived Cardiomyocytes, Impairing Electrical and Mechanical Function

**DOI:** 10.1016/j.stemcr.2021.02.008

**Published:** 2021-02-13

**Authors:** Silvia Marchiano, Tien-Ying Hsiang, Akshita Khanna, Ty Higashi, Leanne S. Whitmore, Johannes Bargehr, Hongorzul Davaapil, Jean Chang, Elise Smith, Lay Ping Ong, Maria Colzani, Hans Reinecke, Xiulan Yang, Lil Pabon, Sanjay Sinha, Behzad Najafian, Nathan J. Sniadecki, Alessandro Bertero, Michael Gale, Charles E. Murry

**Affiliations:** 1 Department of Laboratory Medicine and Pathology, University of Washington, 1959 NE Pacific Street, Seattle, WA 98195, USA; 2 Center for Cardiovascular Biology, University of Washington, 850 Republican Street, Brotman Building, Seattle, WA 98109, USA; 3 Institute for Stem Cell and Regenerative Medicine, University of Washington, 850 Republican Street, Seattle, WA 98109, USA; 4 Center for Innate Immunity and Immune Disease, Department of Immunology, University of Washington School of Medicine, Seattle, WA 98109, USA; 5 Department of Mechanical Engineering, University of Washington, 3720 15th Avenue NE, Seattle, WA 98105, USA; 6 Wellcome – MRC Cambridge Stem Cell Institute, Jeffrey Cheah Biomedical Centre, Cambridge Biomedical Campus, University of Cambridge, Puddicombe Way, CB2 0AW Cambridge, UK; 7 Division of Cardiovascular Medicine, University of Cambridge, Addenbrooke's Hospital, ACCI Level 6, Box 110, Hills Road, Cambridge CB2 0QQ, UK; 8 Sana Biotechnology, 188 E Blaine Street, Seattle, WA 98102, USA; 9 Department of Bioengineering, University of Washington, 3720 15th Avenue NE, Seattle, WA 98105, USA; 10 Department of Medicine/Cardiology, University of Washington, 1959 NE Pacific Street, Seattle, WA 98195, USA

**Keywords:** SARS-CoV-2, COVID-19, hPSC-CMs, human pluripotent stem cell-derived cardiomyocytes, cardiovascular disease, cardiac infection, hESC-CMs, viral myocarditis, arrhythmias and heart failure

## Abstract

COVID-19 patients often develop severe cardiovascular complications, but it remains unclear if these are caused directly by viral infection or are secondary to a systemic response. Here, we examine the cardiac tropism of SARS-CoV-2 in human pluripotent stem cell-derived cardiomyocytes (hPSC-CMs) and smooth muscle cells (hPSC-SMCs). We find that that SARS-CoV-2 selectively infects hPSC-CMs through the viral receptor ACE2, whereas in hPSC-SMCs there is minimal viral entry or replication. After entry into cardiomyocytes, SARS-CoV-2 is assembled in lysosome-like vesicles and egresses via bulk exocytosis. The viral transcripts become a large fraction of cellular mRNA while host gene expression shifts from oxidative to glycolytic metabolism and upregulates chromatin modification and RNA splicing pathways. Most importantly, viral infection of hPSC-CMs progressively impairs both their electrophysiological and contractile function, and causes widespread cell death. These data support the hypothesis that COVID-19-related cardiac symptoms can result from a direct cardiotoxic effect of SARS-CoV-2.

## Highlights

• SARS-CoV-2 selectively infects hPSC-CMs via ACE2, and replicates at high copy number
• SARS-CoV-2 enters hPSC-CMs via endocytosis and fusion, and egresses using lysosomes
• SARS-CoV-2 alters gene expression of hPSC-CMs in a dose- and time-dependent manner
• Electrophysiological and contraction properties decline during SARS-CoV-2 infection

## Graphical Abstract

**Figure:**
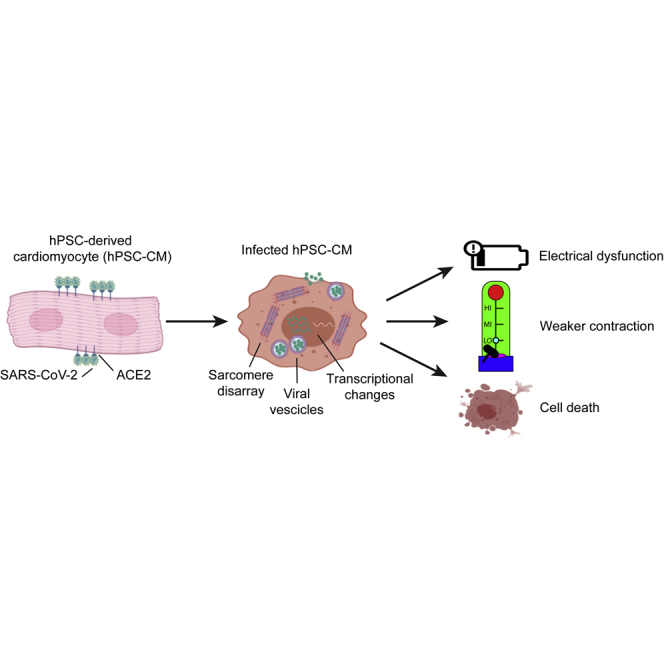

## Teaser

In this article, Marchiano and colleagues show that SARS-CoV-2 can infect human pluripotent stem cell-derived cardiomyocytes (hPSC-CMs), where it replicates to high copy number, significantly impairs electrical and mechanical functions, and eventually causes cell death. This supports the hypothesis that cardiac disease can result from direct infection of the heart.

## Introduction

With over 100 million people affected worldwide, the outbreak of severe acute respiratory syndrome coronavirus 2 (SARS-CoV-2) has already left its permanent mark on human history (Hopkins, 2020; Zhu et al., 2020). SARS-CoV-2 belongs to the family of *Coronaviridae*, a large group of single-stranded enveloped RNA viruses reported for the first time in humans in the 1960s (Andersen et al., 2020; Corman et al., 2018; Cui et al., 2019). Besides being long recognized as one of the common cold viruses, coronaviruses took center stage in infectious disease medicine following the outbreaks of SARS-CoV in 2003 and of Middle East respiratory syndrome coronavirus (MERS-CoV) a decade later. Coronaviruses became thus recognized as highly pathogenic for humans, with a symptomatology that focuses on the respiratory system while often also involving extra-respiratory organs (Alhogbani, 2016; Nishiga et al., 2020; Oudit et al., 2009; Zhou et al., 2020). Indeed, even though the lungs represent the main target, cardiovascular complications (including worsening of pre-existing conditions and onset of new disorders) were not only reported for SARS-CoV and MERS-CoV, but are also significantly contributing to the mortality of COVID-19 patients during the ongoing pandemic (Alhogbani, 2016; Oudit et al., 2009; Shi et al., 2020; Zhou et al., 2020).

The most common cardiovascular complications observed after SARS-CoV-2 infection are myocardial injury (including cases with and without classic coronary occlusion), arrhythmias, and heart failure (Baggiano et al., 2020; Nishiga et al., 2020; Ojha et al., 2020; Ruan et al., 2020; Shi et al., 2020; Wang et al., 2020; Xu et al., 2020; Zhou et al., 2020). In particular, myocardial injury, characterized by elevated serum levels of cardiac troponin I and/or electrocardiogram abnormalities, has been independently associated with increased mortality in COVID-19 patients (Guo et al., 2020). Moreover, as reported also for SARS-CoV (Madjid et al., 2007), SARS-CoV-2 can trigger acute coronary syndrome even in the absence of systemic inflammation (Nishiga et al., 2020; Wang et al., 2020). Retrospective studies show that hospitalized COVID-19 patients develop cardiac arrhythmias, including ventricular tachycardia and atrial fibrillation (Bhatla et al., 2020; Malaty et al., 2020; Wang et al., 2020; Zylla et al., 2021). Progressive left ventricular dysfunction and overall symptoms that resemble heart failure have also been observed in a significant number of patients (Dong et al., 2020; Huang et al., 2020a; Wang et al., 2020; Zhou et al., 2020). At the beginning of the outbreak, this symptomatology was reported mostly in critically ill COVID-19 patients (Zhou et al., 2020). A sizable number of more recent studies has reported that cardiac symptoms are observed also in mild and even asymptomatic cases of COVID-19 (Arentz et al., 2020; Huang et al., 2020b; Inciardi et al., 2020; Puntmann et al., 2020; Rajpal et al., 2021).

The mechanisms behind cardiac disease reported for COVID-19 are still unclear (Nishiga et al., 2020; Zhou et al., 2020). Upon lung infection, the uncontrolled release of inflammatory cytokines, termed “cytokine storm,” could induce multi-organ damage, ultimately leading to organ failure and worsening of pre-existing cardiovascular disorders (Driggin et al., 2020; Inciardi et al., 2020; Tay et al., 2020). Moreover, COVID-19 is associated with coagulopathies, which also can induce ischemic heart damage (Driggin et al., 2020; Nishiga et al., 2020; Ranucci et al., 2020). Finally, SARS-CoV-2 could directly mediate heart injury by entering cardiomyocytes or other cardiac stromal and/or vascular cells via binding of the viral spike glycoprotein to its extracellular receptor, angiotensin I converting enzyme 2 (ACE2) (Baggiano et al., 2020; Hoffmann et al., 2020). This protein is expressed in different tissues of the human body, including cardiomyocytes and cardiac pericytes, and its primary function is to counterbalance the renin-angiotensin-aldosterone system (Chen et al., 2020a; Hikmet et al., 2020; Li et al., 2020; Verdecchia et al., 2020).

Several studies detected SARS-CoV-2 genome in the heart and/or reported signs of viral myocarditis in COVID-19-infected individuals, including asymptomatic cases (Bradley et al., 2020; Dolhnikoff et al., 2020; Lindner et al., 2020; Rajpal et al., 2021). Moreover, *in vivo* and *in vitro* studies utilizing both human adult cardiomyocytes and human pluripotent stem cell-derived cardiomyocytes (hPSC-CMs) have shown that SARS-CoV-2 can infect cardiomyocytes, indicating that SARS-CoV-2 could exhibit cardiac tropism (Bojkova et al., 2020; Chen et al., 2020b; Sharma et al., 2020; Yang et al., 2020). However, whether SARS-CoV-2 infection of human cardiomyocytes leads to a direct impairment of cardiac function is still unresolved. Furthermore, whether other cardiac cell types are also susceptible to SARS-CoV-2 remains unclear.

In this study we examine the mechanisms behind COVID-19-related cardiac symptoms using hPSC-CMs and hPSC-derived smooth muscle cells (hPSC-SMCs), established models for cardiovascular disease research (Bertero et al., 2019b; Cheung et al., 2012; Serrano et al., 2019; Yang et al., 2018). SARS-CoV-2 specifically infects and propagates within hPSC-CMs, a process that appears to exquisitely rely on ACE2 and to both involve direct membrane fusion and entry through the endo-lysosomal pathway. Pathways involved in RNA splicing and chromatin accessibility are significantly upregulated after infection, whereas pathways involved in oxidative metabolism and mitochondrial function are downregulated. SARS-CoV-2 infection results in disruption of the contractile cytoskeleton, electrical and mechanical dysfunction, and eventual cell death. These findings provide evidence for a direct viral cytopathic pathway involving cardiac myocytes in the context of COVID-19-related cardiac disease.

## Results

### hPSC-CMs Express SARS-CoV-2 Receptors and Entry Cofactors

**Figure 1:**
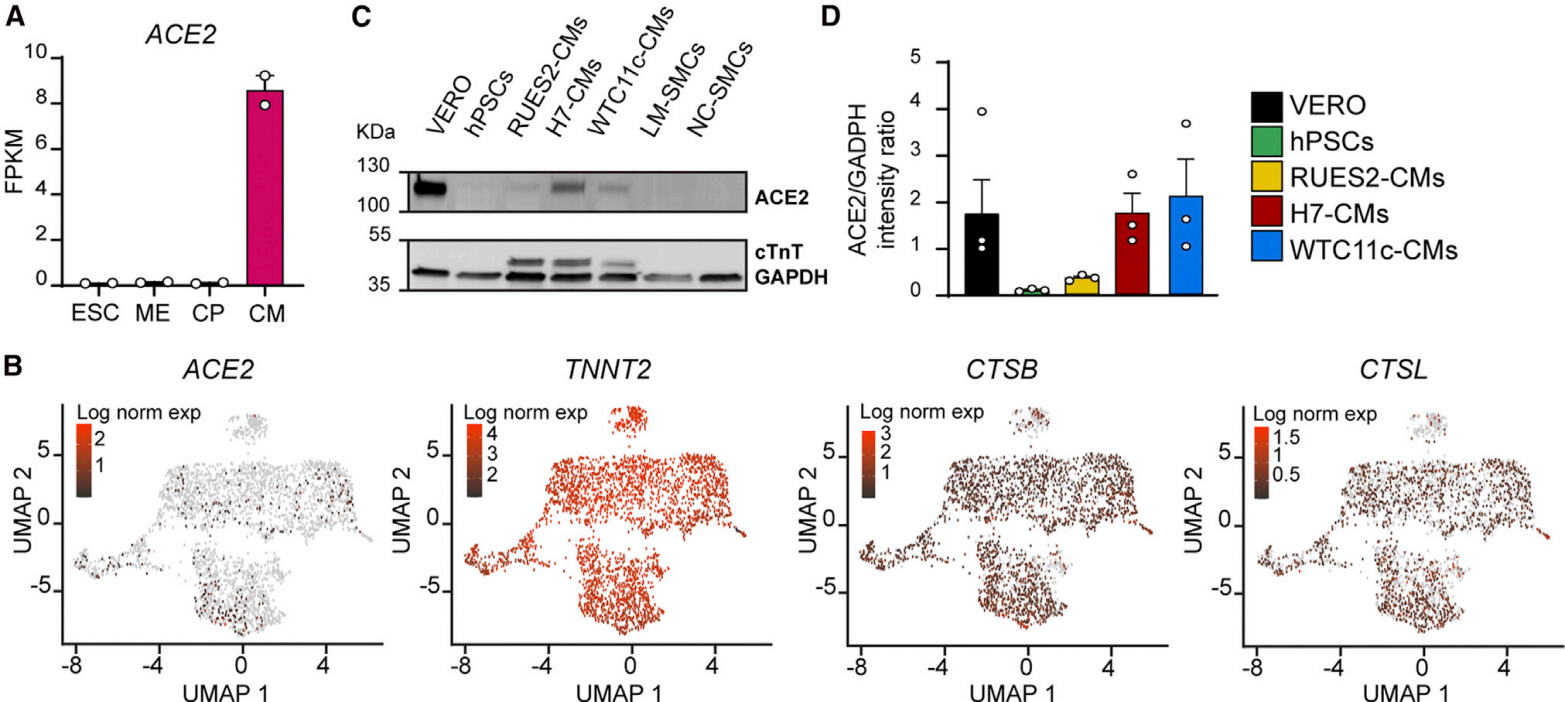
hPSC-CMs Express SARS-CoV-2 Receptors and Processing Factors (A) RNA-seq during RUES2 hESC-CM differentiation. *ACE2* is quantified as fragments per kilobase of transcript per million mapped reads (FPKM). Mean ± SEM of two independent experiments. ESC, embryonic stem cells (day 0); ME, mesoderm (day 2); CP, cardiac progenitors (day 5); CM, cardiomyocytes (day 14). (B) Single-cell RNA-seq gene expression heatmaps from RUES2 hESC-CMs after dimensionality reduction through Uniform Manifold Approximation and Projection (UMAP). *TNNT2* provides a pan-cardiomyocyte marker. (C) Western blot for ACE2 in hPSC-CMs from multiple lines and different types of hPSC-SMCs. LM, lateral plate mesoderm-derived; NC, neural crest-derived; hPSCs, negative control; VERO cells, positive control. (D) Quantification of ACE2 level normalized by GADPH. Mean ± SEM of three independent experiments.

Susceptibility to SARS-CoV-2 infection is thought to depend on expression of both the viral receptor ACE2 and various host proteases (Hoffmann et al., 2020; Millet and Whittaker, 2015; Shang et al., 2020; Zumla et al., 2016). We found that *ACE2* is transcriptionally activated during cardiac differentiation of both RUES2 embryonic stem cell-derived cardiomyocytes (hESC-CMs; Figure 1A) and WTC11c-induced pluripotent stem cell-derived cardiomyocytes (hiPSC-CMs; Figure S1A). Single-cell RNA sequencing (RNA-seq) analysis detected *ACE2* mRNA in ∼9% of hESC-CMs, indicating low and/or transitory expression (Figure 1B). A larger fraction of cells expressed moderate to high levels of endosomal cysteine proteases *CTSB* (cathepsin B; ∼71.0%) and *CTSL* (cathepsin L; ∼46.0%). Detection of these factors is relevant because they can cleave the spike glycoprotein leading to endomembrane fusion-mediated release of the SARS-CoV-2 genome inside the cytoplasm (Kang et al., 2020; Millet and Whittaker, 2015; Ou et al., 2020; Yang and Shen, 2020). Importantly, these viral processing factors were often co-expressed with *ACE2* (Figure S1B). Although viral entry can also be mediated by *TMPRSS2* (Hoffmann et al., 2020; Shulla et al., 2011), this transmembrane serine protease was not detectable in hESC-CMs (Figure S1C), as also reported for the adult human heart (Litvinukova et al., 2020). Interestingly, the lipid phosphatase, *PIKFYVE*, another endosomal viral processing factor, and *FURIN*, a membrane-bound serine protease, were also broadly expressed in hESC-CMs (Figure S1C), overall suggesting that the mechanism of SARS-CoV-2 entry in cardiomyocytes might be different from the TMPRSS2-dependent one reported for lung epithelial cells (Hoffmann et al., 2020; Schneider et al., 2020; Shang et al., 2020; Xia et al., 2020b).

Despite the relatively low levels of mRNA, ACE2 protein was clearly detectable by western blot in hPSC-CMs derived from multiple lines (RUES2 female hESCs, H7 female hESCs, and WTC11c male hiPSCs), reaching levels comparable with those of VERO cells, a primate kidney epithelial line with established SARS-CoV-2 tropism (Figures 1C and 1D). Emphasizing the specific tropism of SARS-CoV-2 for hPSC-CMs, ACE2 was expressed at very low levels in hESC-SMCs of varying embryonic origin (lateral mesoderm- or neural crest-derived, all differentiated from H9 female hESCs; Figures 1C and S1D). Collectively, hPSC-CMs express proteins that may render them susceptible to SARS-CoV-2 infection (Bojkova et al., 2020; Sharma et al., 2020; Yang et al., 2020).

### SARS-CoV-2 Can Infect and Replicate in hPSC-CMs Using ACE2

**Figure 2:**
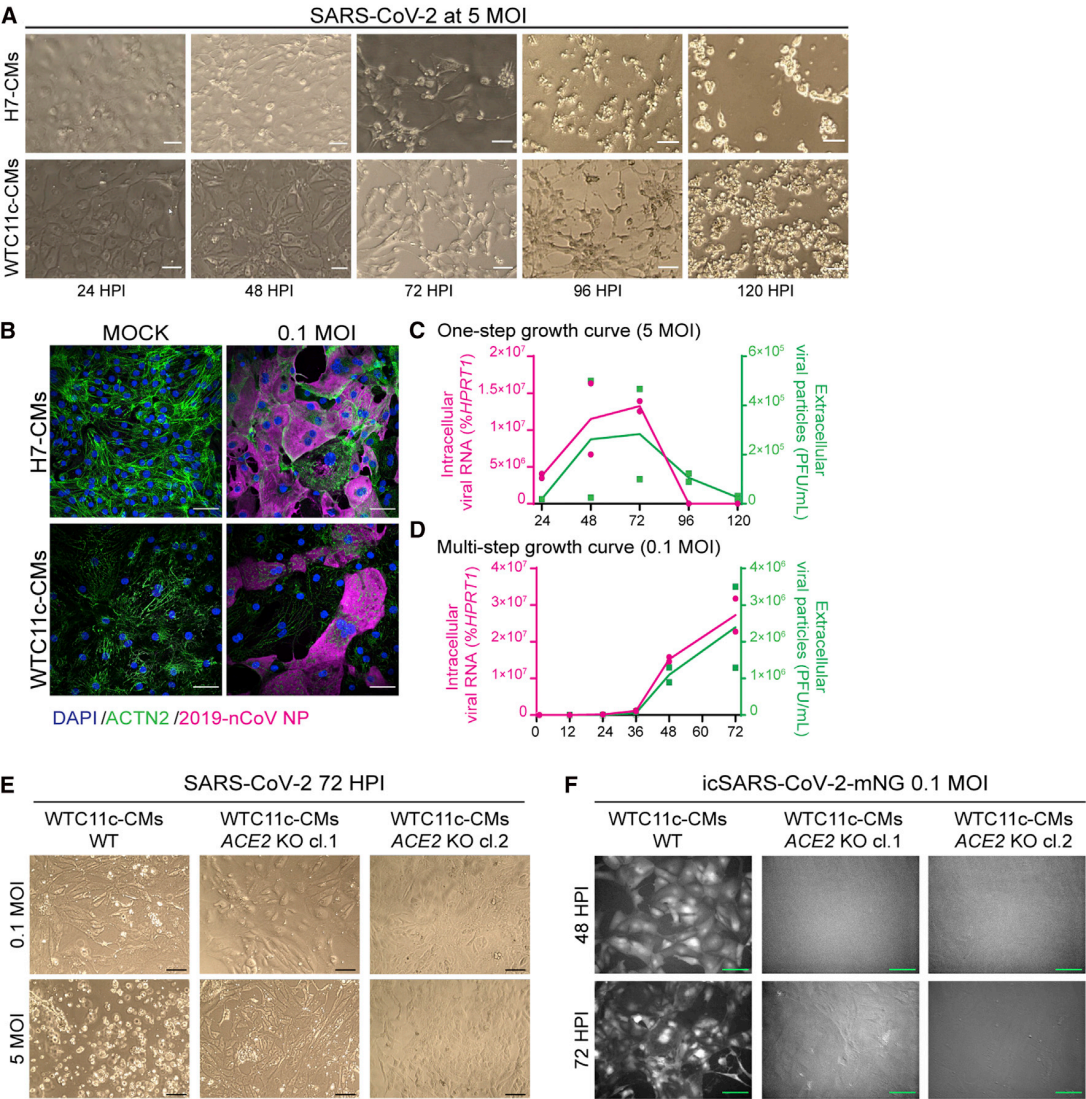
hPSC-CMs Are Permissive to SARS-CoV-2 Infection and Replication (A) Cytopathic effects of SARS-CoV-2 at 5 MOI in H7 hESC-CMs and WTC11c hiPSC-CMs during a time course of 120 h. Scale bars, 100 μm. (B) Immunofluorescent staining of H7 hESC-CMs and WTC11c hiPSC-CMs at 48 hpi with SARS-CoV-2 dose of 0.1 MOI. Scale bars, 50 μm. Individual channels are shown in Figure S2C. (C) One-step viral growth curve in H7 hESC-CMs infected with SARS-CoV-2 at 5 MOI over a time course of 120 h. (D) Multi-step viral growth curve in H7 hESC-CMs infected with SARS-CoV-2 at 0.1 MOI over a time course of 72 h. For both (C) and (D), lines connect the means of two independent experiments. Viral RNA indicating intracellular viral replication is plotted on the left y axis as percent of *HPRT1*. Viral particles secreted in the supernatant are plotted on the right y axis as plaque-forming units (PFU) per mL. (E) Cytopathic effects of 0.1 MOI and 5 MOI of SARS-CoV-2 at 72 hpi in wild-type (WT) and *ACE2* knockout (KO) WTC11c-CMs. Two *ACE2* KO hiPSC clones (cl.1 and cl. 2) were analyzed (Figure S3). Scale bars, 100 μm. (F) Fluorescence microscopy assessment of viral entry of icSARS-CoV2-mNG at 0.1 MOI in WTC11c-CMs WT and *ACE2* KO clones at 48 and 72 hpi. mNG fluorescence is shown in grayscale. ACE2 KO clones showed background autofluorescence only. Scale bars, 100 μm.

Since H7- and WTC11c-derived hPSC-CMs showed the highest levels of ACE2 (Figures 1C and 1D), we tested their functional susceptibility to SARS-CoV-2. For this, we incubated highly pure hPSC-CMs (over 80% positive for cardiac troponin T [cTnT+]; Figure S2A) with SARS-CoV-2/Wa-1 strain. We used a multiplicity of infection (MOI) (i.e., the number of infectious viral particles per cell) of either 0.1 (requiring propagation of the virus within the cells and secondary infection of others) or 5 (aiming to infect all susceptible cells at the same time). We observed marked and disseminated viral cytopathic effects in both H7 hESC-CMs and WTC11c hiPSC-CMs. These effects were accelerated at 5 MOI, as expected (Figures 2A and S2B). Most notably, the highest MOI led to cessation of beating and signs of cell death as early as at 48 h post infection (hpi) in both cell lines, with more pronounced effects for H7 cardiomyocytes. Immunofluorescence staining of SARS-CoV-2 nucleocapsid protein revealed substantial presence of viral factors in the cytoplasm of both H7 hESC-CMs and WTC11c hiPSC-CMs (Figures 2B and S2C).

To investigate whether hPSC-CMs are permissive to SARS-CoV-2 replication, we quantified extracellular viral particles and intracellular viral RNA (by plaque assay and quantitative reverse transcription PCR [qRT-PCR], respectively). The one-step growth curve after 5 MOI infection indicated that viral replication occurred steadily from 24 to 72 hpi, followed by a precipitous decline as the cells died (Figure 2C). The multi-step growth curve (0.1 MOI infection) confirmed that SARS-CoV-2 replicated inside hPSC-CMs (Figure 2D), with a marked increase in viral particles and RNA at 48 and 72 hpi (at which point the experiment was stopped). In agreement with morphological observations, H7-derived cardiomyocytes were more permissive to SARS-CoV-2 replication than WTC11c-derived ones (compare Figures 2C, 2D, S2D, and S2E), perhaps as a reflection of genetic or epigenetic differences that deserve further study.

Distinct from hPSC-CMs, hESC-SMCs exposed to SARS-CoV-2 did not show any cytopathic effects even at the highest MOI at 72 hpi (Figure S2F). Accordingly, extracellular viral particles and intracellular viral RNA in hPSC-SMCs were more than two orders of magnitude lower than the ones observed for hPSC-CMs (Figure S2G). These findings highlight the specific tropism of SARS-CoV-2 for hPSC-CMs, and exclude that cytopathic effects observed in cardiomyocytes may be attributable to toxic contaminants in the viral preparation.

**Figure 3:**
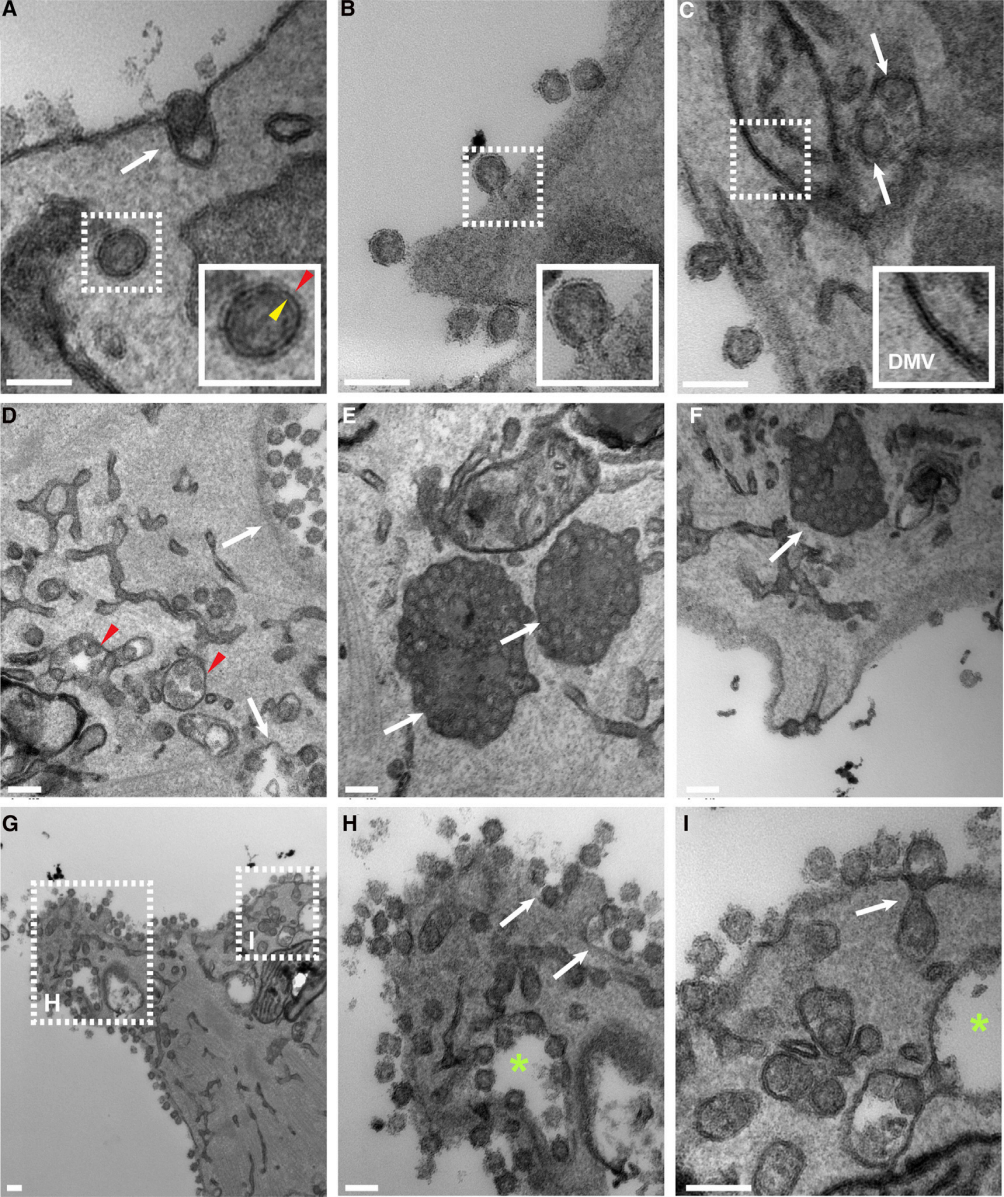
Electron Microscopy Analysis of Infected hPSC-CMs (A) SARS-CoV-2 enters WTC11c-CMs by endocytosis (white arrow). The dashed box (magnified in the inset) shows a virus in an endosome. Note that two lipid layers are identifiable. The outer layer (red arrowhead) belongs to the endosome and the inner layer (yellow arrowhead) to the virus. (B) A virion envelope fuses directly with the cell membrane upon entry (dashed box and magnified view in the inset). (C) A double-membrane vesicle (DMV) (dashed box and magnified in the inset) adjacent to two completely formed virus particles inside a vesicle (arrows). (D–F) Numerous intracellular virus particles packed into different types of vesicles. White arrows in (D) show smooth-walled vesicles, and red arrowheads show vesicles with branched connections, most consistent with the endoplasmic reticulum Golgi intermediate compartment. White arrows in (E and F) show vesicles with viral particles and electron dense content, likely representing lysosomes. (G–I) Virions egressing the cell. Dashed boxes in (G) are magnified in (H and I). The asterisks in (H and I) show smooth-walled vesicles. The white arrows show vesicles opening into the extracellular space (exocytosis), releasing their virions. Scale bars, 100 μm.

To investigate the role of ACE2 during cardiomyocyte infection by SARS-CoV-2, we generated *ACE2* knockout (KO) WTC11c hiPSC-CMs (Figures 3A–3D). Loss of ACE2 prevented cell death following SARS-CoV-2 exposure even at 5 MOI (Figure 2E). To confirm the absence of viral entry in *ACE2* KO cells, we used SARS-CoV-2 genetically engineered to express the mNeonGreen protein (mNG) (Xie et al., 2020). Similarly to wild-type SARS-CoV-2, the SARS-CoV-2-mNG reporter became detectable at 48 hpi at 0.1 MOI only in wild-type cardiomyocytes, with increased fluorescence intensity at 72 hpi (Figure 2F). Overall, these findings indicate that SARS-CoV-2 infection in cardiomyocytes is prominently mediated by the expression of ACE2.

### SARS-CoV-2 Viral Entry, Replication, and Egress Engage Lysosome-like Structures

To examine in finer detail the viral propagation mechanisms in hPSC-CMs we performed extensive electron microscopy analyses. We readily identified numerous viral particles entering and replicating inside the cytoplasm of WTC11c hiPSC-CMs (Figures 3A and 3B), clearly visible as 80–90-nm-wide spherical structures, confirming that hPSC-CMs can be infected directly by SARS-CoV-2. The presence of viruses was remarkably greater at 72 hpi. Interestingly, we observed both endocytosis of intact virions (Figure 3A) and direct fusion of viral envelope with cell membrane (Figure 3B), suggesting dual mechanisms of entry in cardiomyocytes. Double-membrane vesicles, organelles associated with viral replication and assembly, were commonly seen in close association with viral particles (Figure 3C) (Snijder et al., 2020). We also observed dilated membrane-bound tubular structures, likely representing the endoplasmic reticulum Golgi intermediate compartment, in close proximity to membrane-enclosed viral particles (Figure 3D). In addition, we observed a variety of vesicles containing viruses (Figures 3D–3F). Most notable were large vesicles with electron dense content packed with mature virus particles (Figure 3E). In some of these vesicles, we also observed lipid droplets and multilamellar bodies, consistent with lysosomes, whereas others were smooth-walled vesicles (Figures 3E and 3F) (Ghosh et al., 2020; Snijder et al., 2020). Finally, exocytosis of virions was readily identifiable on the cell surface (Figures 3G–3I). In summary, these electron microscopic studies demonstrated viral entry via both direct fusion and endocytosis, replication in subcellular membrane structures, and “hijacking” of lysosomal vesicles for the bulk release of mature virions by exocytosis.

### SARS-CoV-2 Reprograms Chromatin-Modifying, RNA Processing, and Energy Metabolism Pathways

**Figure 4:**
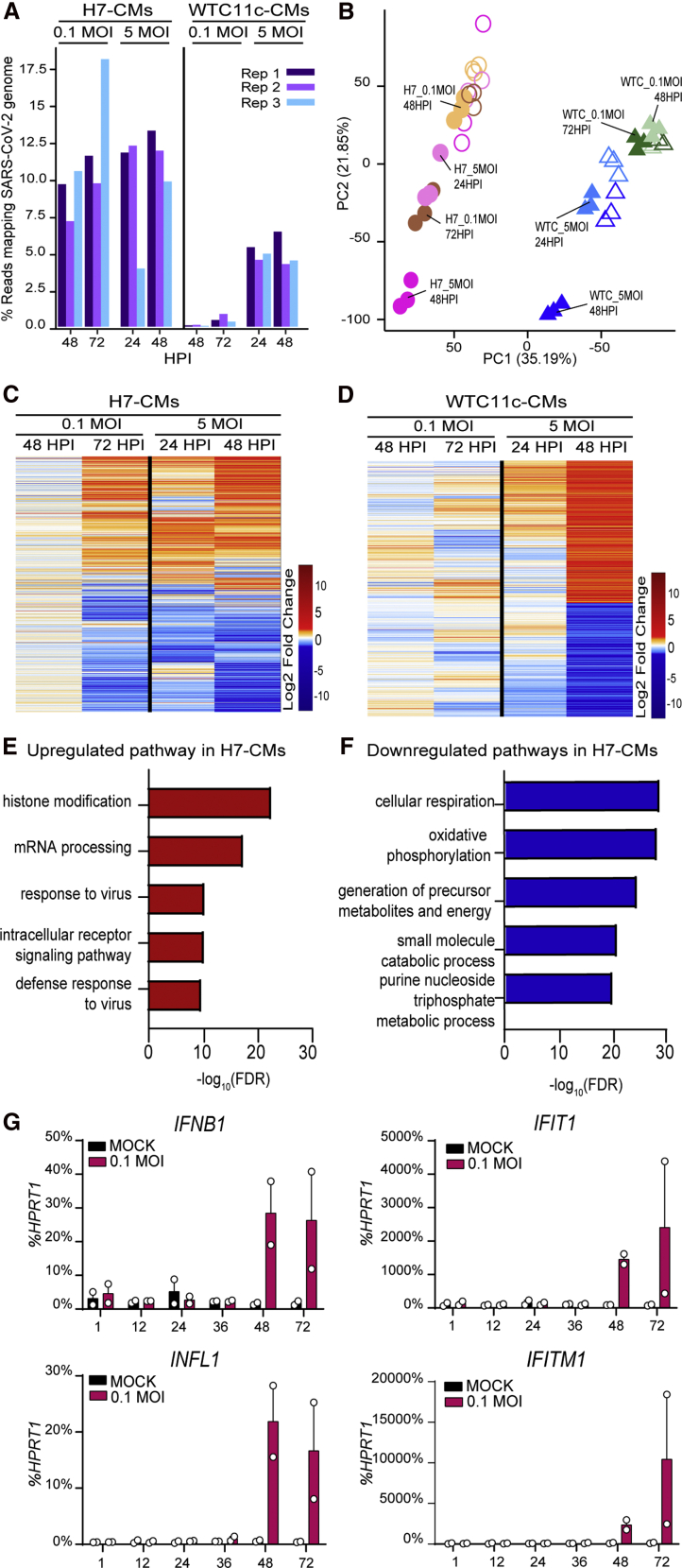
Gene Expression Analysis in hPSC-CMs Infected with SARS-CoV-2 (A) Percentage of RNA-seq reads mapping to the SARS-CoV-2 genome in hPSC-CMs infected with 0.1 or 5 MOI SARS-CoV-2 at different time points (three technical replicates per condition). (B) Principal-component analysis (PCA) plot describing the gene expression variation between samples. Circles and triangles identify H7-CMs and WTC11c-CMs, respectively. The outline of empty symbols (uninfected samples) are color-matched to filled symbols (infected samples) by time point and MOI. (C and D) Heatmaps showing significant differentially expressed genes for infected samples versus mock in H7-CMs (C) and WTC11c-CMs (D). (E) Gene ontology (GO) analysis of upregulated pathways in H7-CMs infected with 5 MOI at 48 hpi. (F) GO analysis of downregulated pathways in H7-CMs infected with 5 MOI at 48 hpi. (**G**) qRT-PCR of interferon response genes in H7 hESC-CMs infected with SARS-CoV-2 at 0.1 MOI. Mean ± SEM of two independent experiments.

To clarify the genome-wide transcriptional alterations induced by SARS-CoV-2 infection in hPSC-CMs, we performed RNA-seq analyses in both H7 hESC-CMs and WTC11c hiPSC-CMs subjected to infection at 0.1 or 5 MOI and sampled every 24 h for 3 days. A high fraction of next-generation sequencing reads mapped to the SARS-CoV-2 genome at high MOI and/or late time points, particularly for H7 hESC-CMs (up to ∼18% of all reads), in agreement with their stronger susceptibility to SARS-CoV-2 infection (Figure 4A). Dimensionality reduction of the data with principal-component analysis (PCA) showed that gene expression variability correlated most strongly with the cell type of origin (PC1) and subsequently with the degree and progression of SARS-CoV-2 infection (PC2) (Figure 4B). Accordingly, gene expression profiles change in a time- and dose-dependent manner for both H7-CMs and WTC11c-CMs, with major differences observed at the latest time point and with the highest MOI (Figures 4C and 4D). Interestingly, pathways involved in RNA processing and chromatin accessibility (i.e., histone modification) were upregulated together with the response to viral infection, suggesting that SARS-CoV-2 may induce broad epigenetic reprogramming of the host to promote its own replication (Figures 4E and S4A; Tables S1 and S3) (Banerjee et al., 2020). In addition, we observed that genes involved in mitochondrial function and energy production were downregulated (Figures 4F and S4B; Tables S2 and S4), indicating that SARS-CoV-2 might promote a shift toward a glycolytic metabolism by suppressing mitochondrial oxidative phosphorylation, which could also favor its replication (Ajaz et al., 2021; Icard et al., 2021).

Upon viral infection, pathogen-associated molecular patterns (PAMPs) initiate the early immune response via host pattern recognition receptors. After virus uncoating, RIG-I-like receptors bind to the uncapped and double-stranded viral RNA in the cytosol and trigger innate immune activation, leading to the production of type I and type III interferons and the interferon-induced antiviral response (Loo and Gale, 2011). Upregulated pathways after SARS-CoV-2 infection in hPSC-CMs included those involved in viral defense (Figure 4E). To more finely clarify the underlying kinetics, we analyzed the interferon response from 2 to 72 hpi by qRT-PCR. We found that interferon transcripts (*INFB1* and *IFNL1)* were markedly upregulated at 48 and 72 hpi in both cell lines, with a stronger effect in the more sensitive H7 cardiomyocytes (Figures 4G and S4C). The interferon-stimulated genes *IFIT1* and *IFITM1* were also upregulated at the latest time point. These results indicate that SARS-CoV-2 induces innate immune activation and interferon response in hPSC-CMs, similar to other cell types (Lei et al., 2020).

### Electrophysiological Characteristics of hPSC-CMs Infected with SARS-CoV-2

**Figure 5:**
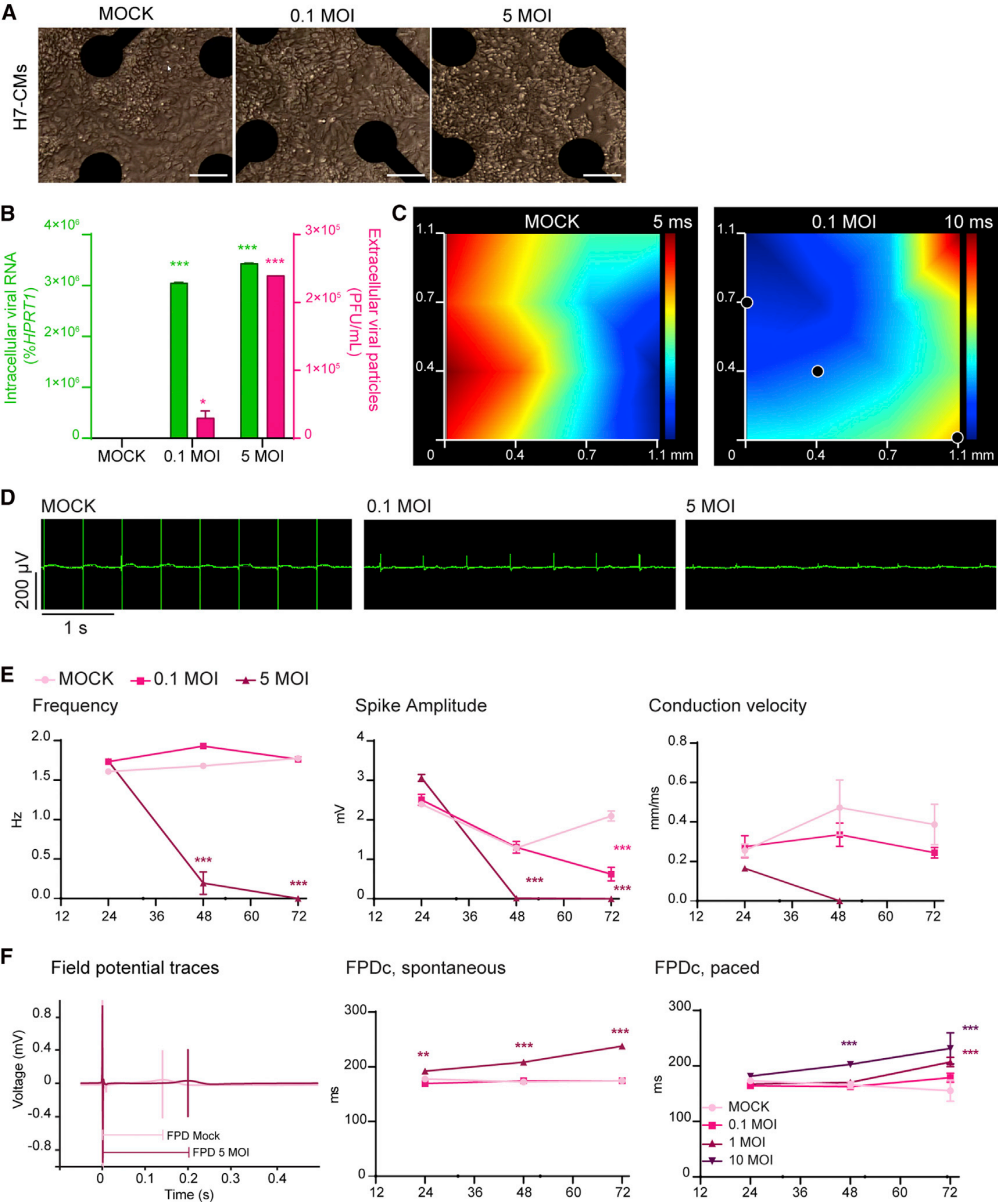
Electrophysiological Alterations in hPSC-CMs Infected with SARS-CoV-2 (A and B) (A) Representative images of SARS-CoV-2-infected H7 hESC-CMs on MEA wells at 72 hpi. Scale bars, 50 μm. (B) Intracellular and extracellular viral particles in H7 hESC-CMs seeded and infected in the MEA plate. Intracellular viral particles are plotted on the right axis as viral RNA (percent of *HPRT1*); extracellular viral particles are plotted on the left axis as PFU/mL. Data are shown as mean ± SEM of eight wells. Differences versus mock control by two-way ANOVA with Sidak correction for multiple comparisons (^∗^p < 0.05, ^∗∗∗^p < 0.001). (C) Representative propagation maps at 72 hpi. The axes illustrate the position of 16 total electrodes, with black dots indicating inactive ones. Electrical propagation starts in the blue area and moves toward the red area, according to the color-coded time interval. (D) Representative recordings of spontaneous electrical activity of SARS-CoV-2-infected H7 hESC-CMs at 72 hpi. (E and F) (E) Representative quantifications of electrophysiological properties from MEA analyses in SARS-CoV-2-infected H7 hESC-CMs. Mean ± SEM of eight wells. Statistical analyses of intra-experimental variability as for (B). (F) Representative field potential traces in SARS-CoV-2-infected H7 hESC-CMs at 72 hpi, and quantification of FPD corrected by beat rate in spontaneous and paced experiments. Mean ± SEM of eight and six wells for spontaneously beating and paced cells, respectively. Statistical analyses of intra-experimental variability as for (B) (^∗∗^p < 0.01).

We next investigated whether SARS-CoV-2 infection impairs the function of hPSC-CMs. First, we evaluated electrophysiological properties of infected H7 hESC-CMs and WTC11c hiPSC-CMs using multi-electrode arrays (MEA) over a time course of 72 hpi at MOI of 0.1 and 5. Distinct from our earlier experiments on sparser hPSC-CM cultures in standard tissue culture dishes, in this context we did not observe profound cytopathic effects in any of the conditions (Figures 5A and S5A). This outcome may reflect a decreased efficiency of viral propagation as hPSC-CMs were plated at high density to ensure robust assessment of electrophysiological properties. Nevertheless, viral RNA and viral particles could still be detected at 0.1 MOI, with the highest levels at 5 MOI (Figures 5B and S5B), showing that the infection occurred also in these highly dense cultures. Representative propagation maps are shown in Figures 5C and S5C, while representative field potential recordings showcasing changes in spike amplitude and frequency are included in Figures 5D and S5D. Remarkably, SARS-CoV-2 infection rapidly resulted in reduced beating rate, lower depolarization spike amplitude, and decreased electrical conduction velocity (Figures 5E, S5E, and S5F). In H7 hESC-CMs we also observed a time-dependent increase in the field potential duration (FPD) both in spontaneously beating and electrically paced cultures (Figure 5F; similar measurements could not be reliably obtained from WTC11c hiPSC-CMs due to the limited amplitude of the repolarization wave after SARS-CoV-2 infection). Overall, abnormalities in the generation and propagation of electrical signals were significant even in the absence of extensive cell death, suggesting that SARS-CoV-2 infection in cardiomyocytes could directly create a substrate for arrhythmias (Bhatla et al., 2020; Zylla et al., 2021).

### SARS-CoV-2 Infection Progressively Impairs Force Generation in Engineered Heart Tissues

**Figure 6:**
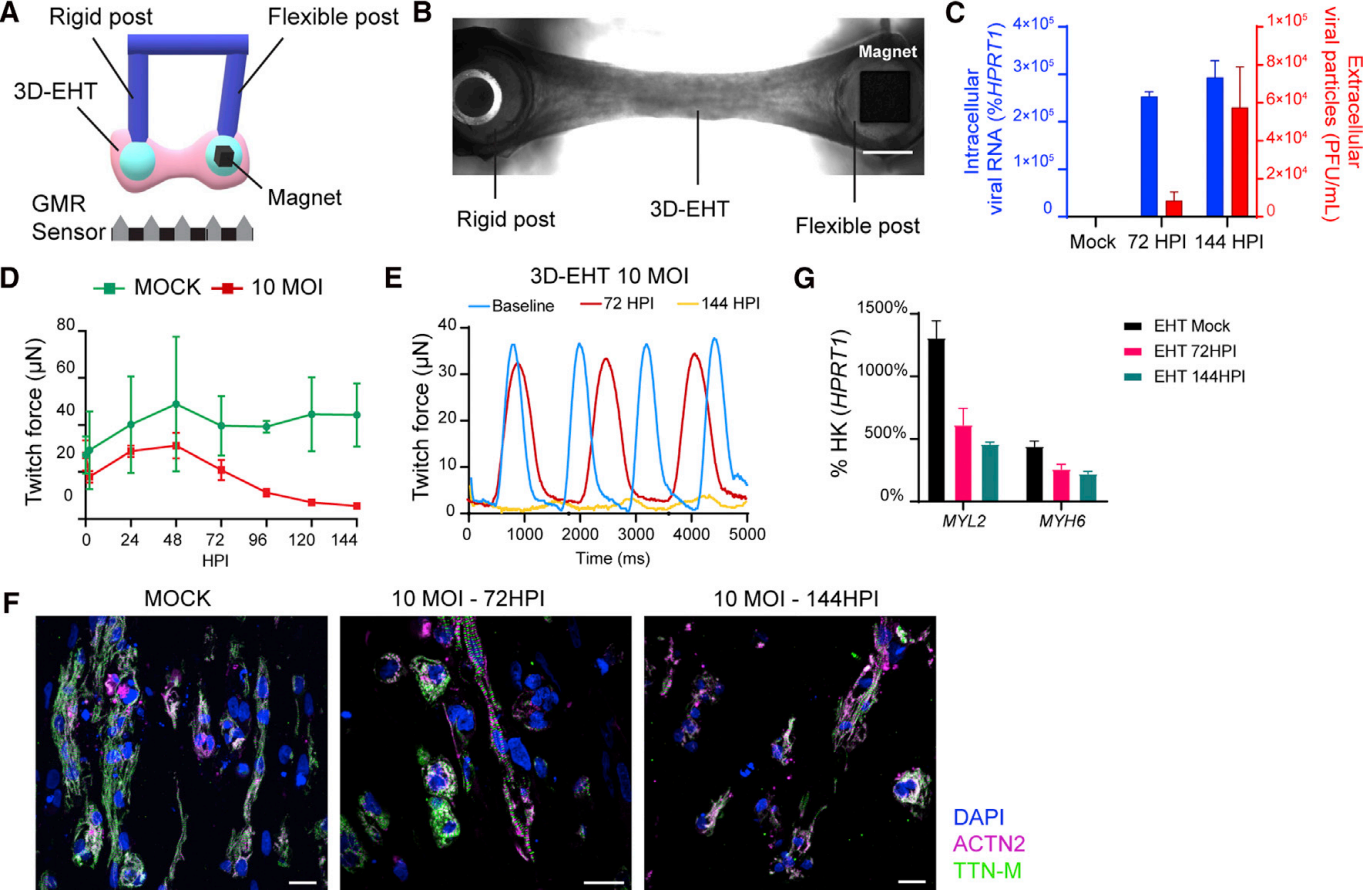
Force Production of 3D-EHTs Made from hiPSC-CMs Progressively Declines during SARS-CoV-2 Infection (A) Schematic representation of the magnetic sensing system: the 3D-EHT is suspended between two posts (one rigid, one flexible). The magnet is localized inside the flexible post, and the post movement during 3D-EHT contraction is recorded by giant magnetoresistance (GMR) sensor located at the bottom of the dish (converting displacement into voltage changes). (B) Representative picture of a 3D-EHT. Scale bar, 1 mm. (C) SARS-CoV-2 viral RNA and particles detected in 3D-EHTs infected at 10 MOI at specified time points. Intracellular viral RNA is plotted on the right axis as percent *HPRT1*; extracellular viral particles are plotted on the right axis as PFU/mL. Mean ± SEM of two 3D-EHTs per condition. (D) Representative time course analysis of twitch force in 3D-EHTs from WTC11c hiPSC-CMs after SARS-CoV-2 infection at 10 MOI. Data are shown as mean ± SEM for two mock controls and four infected 3D-EHTs. (E) Representative twitch traces of 3D-EHT at 10 MOI at different time points. (F and G) (F) Immunofluorescent images of 3D-EHTs sections at different time points. Scale bars, 20 μm. (G) qRT-PCR of sarcomeric genes in 3D-EHTs infected with SARS-CoV-2 at 10 MOI. Mean ± SEM of two 3D-EHTs.

We then evaluated the contractile properties of hPSC-CMs using three-dimensional engineered heart tissues (3D-EHTs), following their contractile behavior through magnetic field sensing (Bielawski et al., 2016) (Figures 6A and 6B). For these experiments we focused on WTC11c hiPSCs since 3D-EHTs from H7 hESC-CMs proved to beat spontaneously at too high a frequency (>2 Hz) to enable accurate measurements of contractile behavior (i.e., the tissue had a tetanic-like contraction with minimal relaxation between beats at this frequency). We infected 3D-EHTs from WTC11c hiPSC-CMs with 10 MOI (to facilitate infection within the non-vascularized, cell-dense tissue), and analyzed their contraction for a week. This 3D model experienced viral replication comparable with that of 2D cultures, highlighting once again the cardiac tropism of SARS-CoV-2 (Figure 6C). The maximal twitch force in infected tissues decreased as early as 72 hpi (Figure S6A), and the contractions continued to subside to less than 25% of the force measured at the baseline at 144 hpi (Figures 6D and 6E; Videos S1 and S2). Cardiomyocyte density progressively decreased while cells also became more rounded (i.e., dedifferentiated) and less aligned with the longitudinal axis of the 3D-EHTs (Figures 6F and S6B). This could collectively contribute to the loss of force production. Infected 3D-EHTs also showed decreased expression of the sarcomeric genes *MYL2* and *MYH6*, which may be correlated to the loss of sarcomere organization (Figure 6G). Overall, the significant impairment in the contractile properties of 3D-EHTs demonstrates that the mechanical function of cardiomyocytes is impacted by SARS-CoV-2 infection *in vitro*, and suggest that similar mechanisms could contribute to whole-organ cardiac dysfunction also in patients (Dong et al., 2020).

Video S1. 3D-EHT before SARS-CoV-2 Infection

Video S2. SARS-CoV-2-Infected 3D-EHT at 144 hpi

## Discussion

A rapidly increasing number of reports acknowledge cardiovascular involvement as a prevalent complication observed in COVID-19 patients, but discriminating between direct versus indirect effects is still an open challenge (Nishiga et al., 2020; Shi et al., 2020; Tay et al., 2020). In this study, we show that SARS-CoV-2 has the ability to directly infect cardiomyocytes, to impair both their electrophysiological and contractile properties, and to eventually induce cell death.

In agreement with earlier reports, we find that cardiomyocytes (but not smooth muscle cells) express ACE2, making them susceptible to SARS-CoV-2 infection. Our experiments in ACE2 KO hPSC-CMs formally demonstrate the key role of this factor for SARS-CoV-2 entry in this cell type. Interestingly, mRNA levels of ACE2 are heterogeneous within hPSC-CMs from the same culture, and the resulting protein is differentially abundant in hPSC-CMs from different genetic backgrounds. The cytopathic effects of SARS-CoV-2 infection also strongly vary between cardiomyocytes derived from different hPSC lines. ACE2 expression and SARS-CoV-2 susceptibility may be similarly heterogeneous *in vivo*, both across different regions of the heart and within different subjects (Litvinukova et al., 2020). This might partially explain the discrepancy between the strong prevalence of heart damage in COVID-19 patients and the limited evidence for viral particles in the heart found by autopsy examinations. We suggest that future analyses should aim to sample various regions of the heart and focus on those patients that had shown the strongest cardiac symptoms.

A puzzling observation is that the cytopathic effects of SARS-CoV-2 infection expands to virtually the entire monolayer of hPSC-CMs, even though single-cell RNA-seq indicates that many cardiomyocytes do not express detectable levels of *ACE2*. One possible explanation is that ACE2 transcription is episodic and/or still meaningful low levels that are below the sensitivity of single-cell RNA-seq (either way resulting in sufficient protein levels to allow SARS-CoV-2 entry). Alternatively, cytotoxic stimuli triggered by SARS-CoV-2 might spread to the adjacent cells via gap junctions or through the supernatant as toxic cytokines and/or other danger signals. Finally, the fusogenic properties of the SARS-CoV-2 spike protein may mediate membrane fusion not only between viral and host membranes but also within cardiomyocytes, leading to intercellular viral spreading (Schneider et al., 2020). Spike protein fusogenicity is secondary to its proteolytic cleavage by host proteases, such as furin (Millet and Whittaker, 2015), which is expressed in hPSC-CMs. Noticeably, we observed direct fusion between the virus and hPSC-CMs, a process that may be mediated by proteolytically primed spike proteins, as in the case of MERS-CoV (Millet and Whittaker, 2014; Xia et al., 2020a, 2020b). Future studies are needed to clarify whether any of these non-mutually exclusive mechanisms are involved in the strong susceptibility of hPSC-CMs to SARS-CoV-2 infection, and whether similar events are recapitulated *in vivo*.

We found that hPSC-CMs are also extremely permissive to viral replication. Among other types of ultrastructural features, we detected the presence of double-membrane vesicles. These are used by viruses both to concentrate their building materials for efficient replication, and to evade the immune surveillance by “hiding” viral factors that can trigger the PAMP pathway (Wolff et al., 2020). Accordingly, we observed activation of interferon-responsive genes only at late time points of SARS-CoV-2 infection. The interferon response, which is part of the innate immune response activation (Loo and Gale, 2011), usually occurs within hours from viral infection. The fact that cardiomyocytes infected with SARS-CoV-2 show a delayed response may facilitate viral replication to high levels (Lei et al., 2020). Furthermore, RNA-seq showed that SARS-CoV-2 infection affects pathways involved in RNA regulation. SARS-CoV-2 can impair RNA splicing to evade the intracellular innate immune response (Banerjee et al., 2020), which may be the case also in hPSC-CMs. Moreover, oxidative phosphorylation and mitochondrial function are severely downregulated in the infected cells. SARS-CoV-2 may shift cellular metabolism to promote glycolytic metabolic activity in support of viral replication (Icard et al., 2021), providing yet another way to boost its replication also in hPSC-CMs.

The presence of highly replicating virus severely affects both the morphology and the function of hPSC-CMs. Infected cardiomyocytes lose cytoskeletal organization, become packed with different types of vesicles, and show broad alterations of gene expression. Using an MEA system, which has been validated to detect potential arrhythmogenic properties of novel drugs (Blinova et al., 2018), we identified several electrophysiological abnormalities induced by SARS-CoV-2 infection. Prolongation of FPD is particularly noticeable. This measurement reflects the interval between membrane depolarization and repolarization, and as such represents an *in vitro* surrogate of the QT interval measured by an electrocardiogram. It is well known that prolongation of the QT interval is pro-arrhythmogenic (Chiang and Roden, 2000). Thus, FPD prolongation in SARS-CoV-2-infected hPSC-CMs may be an *in vitro* surrogate phenotype mirroring the arrhythmias observed in ∼20% of COVID-19 patients (Malaty et al., 2020). Last, but not least, we found marked impairment in both contractile function and histological organization in 3D-EHTs infected with SARS-CoV-2. If similar effects were to occur in the hearts of some COVID-19 patients, this could contribute to cardiac dysfunction. Overall, hPSC-CMs on MEAs and/or organized in 3D-EHTs may represent valuable scalable platforms to identify active compounds that may provide therapeutic value.

Collectively, our results support the notion that, independent of inflammation or coagulopathy, SARS-CoV-2 can cause direct functional heart damage by either inducing cell death and/or by impairing electro-mechanical functions. One limitation of this study is our reliance on hPSC-CMs, which are well known for their functional immaturity (Guo and Pu, 2020; Karbassi et al., 2020; Marchiano et al., 2019). While the *in vitro* systems we used have been successfully leveraged to model electrophysiological and contractile alterations due to drugs or inherited mutations (Blinova et al., 2018; Paik et al., 2020), their application to modeling COVID-19 still requires further validation. Nevertheless, a recent report by Dolhnikoff et al. (2020) identified coronaviral particles in the cytoplasm of cardiomyocytes, endothelial cells, and fibroblasts by electron microscopy in the heart of an 11-year-old child who died from multi-system inflammatory syndrome in children following COVID-19 infection. This indicates that *in vivo* cardiomyocytes with substantially greater maturity than used here are susceptible to SARS-CoV-2 infection. COVID-19 patients are commonly treated with steroids to control systemic inflammation. However, our data suggest that treatments aimed to control the direct damage of SARS-CoV-2, not only by preventing infection but also by preventing viral replication or rescuing cardiac function, should also be taken into consideration to prevent long-term cardiovascular complications.

## Experimental Procedures

### Cell Culture

RUES2 hESCs and WTC11c hiPSCs were maintained and differentiated using small-molecule modulators of the WNT pathway (Bertero et al., 2019a). H7 hESCs were differentiated in suspension culture format by collaborators at the Center for Applied Technology Development at the City of Hope in California. H9 hESCs were maintained and differentiated into lateral mesoderm- and neural crest-derived SMCs as described previously (Bargehr et al., 2016; Serrano et al., 2019). ACE2 KO clones were generated using CRISPR-Cas9 ribonucleoprotein complexes (Synthego).

### Gene Expression Analysis

Bulk RNA-seq datasets from RUES2 hESC-CMs had been previously generated and analyzed (Bertero et al., 2019a). Bulk mRNA-seq data from infected cardiomyocytes were generated by constructing mRNA-seq libraries using the KAPA mRNA HyperPrep Kit (Kapa Biosystems). Libraries were sequenced on an Illumina NovaSeq. For single-cell RNA-seq analysis, a single-cell suspension was generated from RUES2 hESC-CMs and single-cell RNA-seq was performed using the Chromium Next GEM Single Cell 3′ Kit (10X Genomics). For real-time qRT-PCR, RNA from infected cardiomyocytes was harvested using TRIzol reagent. cDNA was obtained with M-MLV reverse transcriptase (Invitrogen), and qRT-PCR was performed with SYBR Select Master Mix (Applied Biosystems). Primers are reported in Table S5.

### Western Blot

hPSC-CMs were lysed using RIPA Buffer. Samples were run on mini-PROTEAN TGX precast gels (Bio-Rad) and then transferred onto polyvinylidene fluoride membranes. Primary and secondary antibodies (Supplemental Experimental Procedures) were incubated in blocking buffer, and fluorescent signals were acquired using a GelDoc Imager (Bio-Rad).

### SARS-CoV-2 Generation

All experiments using live virus were performed in the Biosafety Level 3 (BSL-3) facility at the University of Washington in compliance with the BSL-3 laboratory safety protocols (CDC BMBL 5^th^ ed.) and the recent CDC guidelines for handling SARS-CoV-2. SARS-related coronavirus 2, Isolate USA-WA1/2020 (SARS-CoV-2) and icSARS-CoV-2mNG were obtained from BEI Resources (NR-52281) and the University of Texas (Xie et al., 2020), respectively, and propagated in VERO cells (USAMRIID). Viral preparations and culture supernatant from SARS-CoV-2-infected cardiomyocytes were titered using a plaque assay.

### Viral Infection

SARS-CoV-2 wild-type or expressing mNeonGreen protein was diluted to the desired MOI in DMEM and incubated on hPSC-CMs or hPSC-SMCs for 1 h at 37°C (non-infected [mock] controls were incubated with DMEM only). Cells were then washed with Dulbecco's Phosphate-buffered Saline (DPBS) and cultured in the appropriate maintenance media.

### Immunofluorescence

hPSC-CMs were fixed with 4% paraformaldehyde (PFA) and permeabilized using 0.25% Triton X-100 (Sigma-Aldrich) before staining with primary and secondary antibodies (Supplemental Experimental Procedures).

### Electron Microscopy

hPSC-CMs were fixed with Karnovsky's fixative. Heavy metal impregnation was performed as detailed elsewhere (Deerinck et al., 2010). Thin sections were viewed using a JEOL 1230 transmission electron microscope.

### Electrophysiological Analysis with MEA

CytoView MEA plates (Axion BioSystems) were coated with Matrigel and hPSC-CMs were plated as described previously (Bertero et al., 2019b). SARS-CoV-2 effects on hPSC-CMs electrophysiology were recorded using the Maestro Pro system and analyzed with Cardiac Analysis Software v.3.1.8 (all from Axion BioSystems).

### 3D-EHTs Analysis

Post-suspended, fibrin-based 3D-EHTs were generated with hPSC-CMs and HS27a stromal cells (ATCC) at a 1:10 ratio. Twitch force was recorded by tracking the movement of magnets embedded in the flexible posts, as described previously (Bielawski et al., 2016). For immunostaining, 3D-EHTs were arrested in diastole, fixed in 4% PFA, and embedded in Tissue-Tek O.C.T. before sectioning and staining.

### Statistical Analyses

Statistical analyses were performed only for experiments with more than two replicates using Prism 8.1.3 (GraphPad). The type and number of replicates, the statistics plotted, the statistical test used, and the test results are described in the figure legends.

### Data and Code Availability

The data supporting this publication have been made available in GEO under accession numbers: GSE157175 (single-cell RNA-seq) and GSE162736 (bulk RNA-seq).

## Author Contributions

S.M. performed hiPSC-CM differentiation, viral infections, western blots, viral plaque, MEA and 3D-EHTs assays, and wrote the first draft of the manuscript. T.-Y.H. cultured and expanded SARS-CoV-2, performed immunofluorescence and collected samples for RNA-seq. A.K. generated and characterized *ACE2* KO cell lines and performed qRT-PCR. T.H. designed and fabricated the 3D-EHT magnetic sensing system, and analyzed the data. L.S.W., J.C., and E.S. performed library preparation and RNA-seq data analysis. J.B., H.D., M.C., and L.P.O. differentiated hPSC-SMCs and contributed to experimental design. H.R. contributed to experimental design and differentiated hiPSC-CMs. X.Y. contributed to experimental design and analyzed both bulk and single-cell RNA-seq data. L.P. and S.S. contributed to experimental design and supervised the experiments. B.N. performed electron microscopy and contributed to data analysis and writing the manuscript. A.B. contributed to the study conception, experimental design and execution, and assisted in writing the manuscript. N.J.S., M.G., and C.E.M. conceived and supervised the study, obtained research funding, and contributed to data analysis and writing the manuscript.

## Supplemental Information

Document S1. Supplemental Experimental Procedures, Figures S1–S6, and Tables S1–S5

Document S2. Article plus Supplemental Information
