# Supplementary material for: SARS-CoV-2 Infects Human Pluripotent Stem Cell-Derived Cardiomyocytes, Impairing Electrical and Mechanical Function
Source: Stem Cell Reports. 2021 Feb 13;16(3):478–92. doi: 10.1016/j.stemcr.2021.02.008 (PMC7881699; doi:10.1016/j.stemcr.2021.02.008)
Supplement: Document S1. Supplemental Experimental Procedures, Figures S1–S6, and Tables S1–S5 [file mmc1.pdf]

Stem Cell Reports, Volume *16*

Supplemental Information

SARS-CoV-2 Infects Human Pluripotent Stem Cell-Derived Cardiomyocytes, Impairing Electrical and Mechanical Function

Silvia Marchiano, Tien-Ying Hsiang, Akshita Khanna, Ty Higashi, Leanne S. Whitmore, Johannes Bargehr, Hongorzul Davaapil, Jean Chang, Elise Smith, Lay Ping Ong, Maria Colzani, Hans Reinecke, Xiulan Yang, Lil Pabon, Sanjay Sinha, Behzad Najafian, Nathan J. Sniadecki, Alessandro Bertero, Michael Gale Jr., and Charles E. Murry

Supplementary Information

Supplementary Figure 1 – hPSC-CMs express SARS-CoV-2 receptors and processing factors.

Supplementary Figure 2 – hPSC-CMs are permissive to SARS-CoV-2 infection and replication.

Supplementary Figure 3 – Genotyping and validation of WTC11c hiPSC *ACE2* knockout clones.

Supplementary Figure 4 – Gene expression analysis of WTC11c-CMs infected with SARS-CoV-2.

Supplementary Figure 5 – Electrophysiological alterations in hPSC-CMs infected with SARS-CoV-2.

Supplementary Figure 6 – WTC11c hiPSC-CMs 3D-EHTs infected with SARS-CoV-2.

Supplementary Table 1 – Extended data from GO analysis (upregulated pathways for H7-CMs).

Supplementary Table 2 – Extended data from GO analysis (downregulated pathways for H7-CMs).

Supplementary Table 3 – Extended data from GO analysis (upregulated pathways for WTC11c-CMs).

Supplementary Table 4 – Extended data from GO analysis (downregulated pathways for WTC11c-CMs).

Supplementary Table 5 – RT-qPCR primer sequences.

Supplemental Experimental Procedures

Supplemental References

Supplementary Video 1 – 3D-EHT before SARS-CoV-2 infection (online).

Supplementary Video 2 – SARS-CoV-2 infected 3D-EHT at 144 HPI (online).

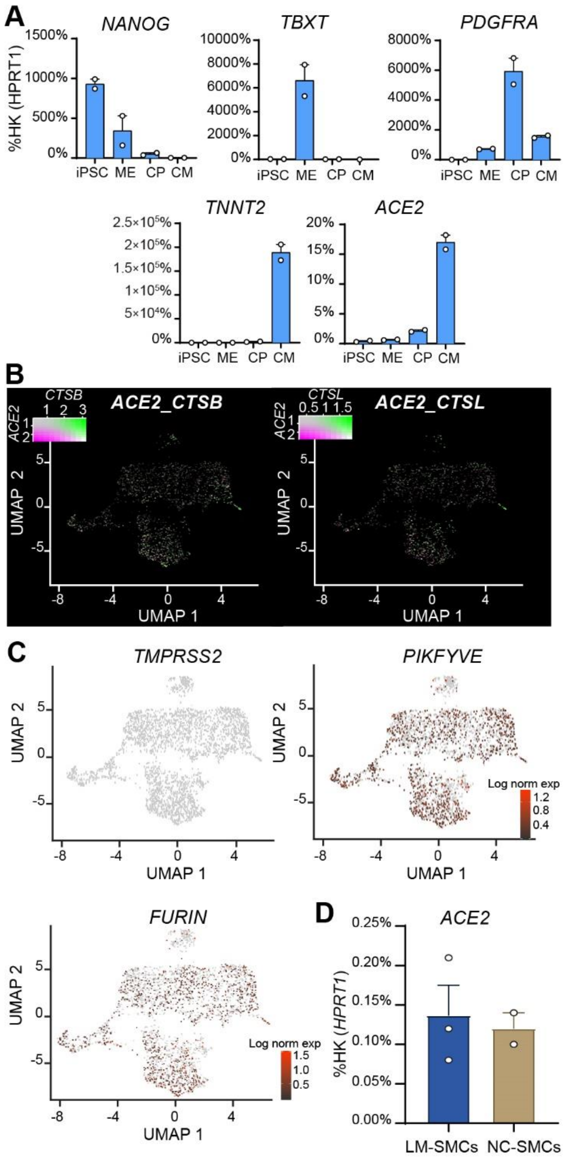

Supplementary Figure 1 – hPSC-CMs express SARS-CoV-2 receptors and processing factors. (**A**) RT-qPCR analysis during WTC11c hiPSC-CM differentiation (two independent experiments). iPSC: induced pluripotent stem cells (day 0); ME: mesoderm (day 2); CP: cardiac progenitors (day 5); CM: cardiomyocytes (day 14). Mean ± SEM of two independent experiments. (**B-C**) sc-RNA-seq gene expression heatmaps from RUES2 hESC-CMs after dimensionality reduction through UMAP. In B, plots showcase double positive cells for *ACE2* and *CTSL*, and *ACE2* and *CTSB*. Related to Figure 1B. (**D**) RT-qPCR analysis of H9 hESC-SMCs (LM: lateral plate mesoderm-derived; NC: neural crest-derived). Mean ± SEM of 3 differentiation batches. In one batch of NC-SMCs, *ACE2* was undetectable (Ct > 40). Related to Figure 1C.

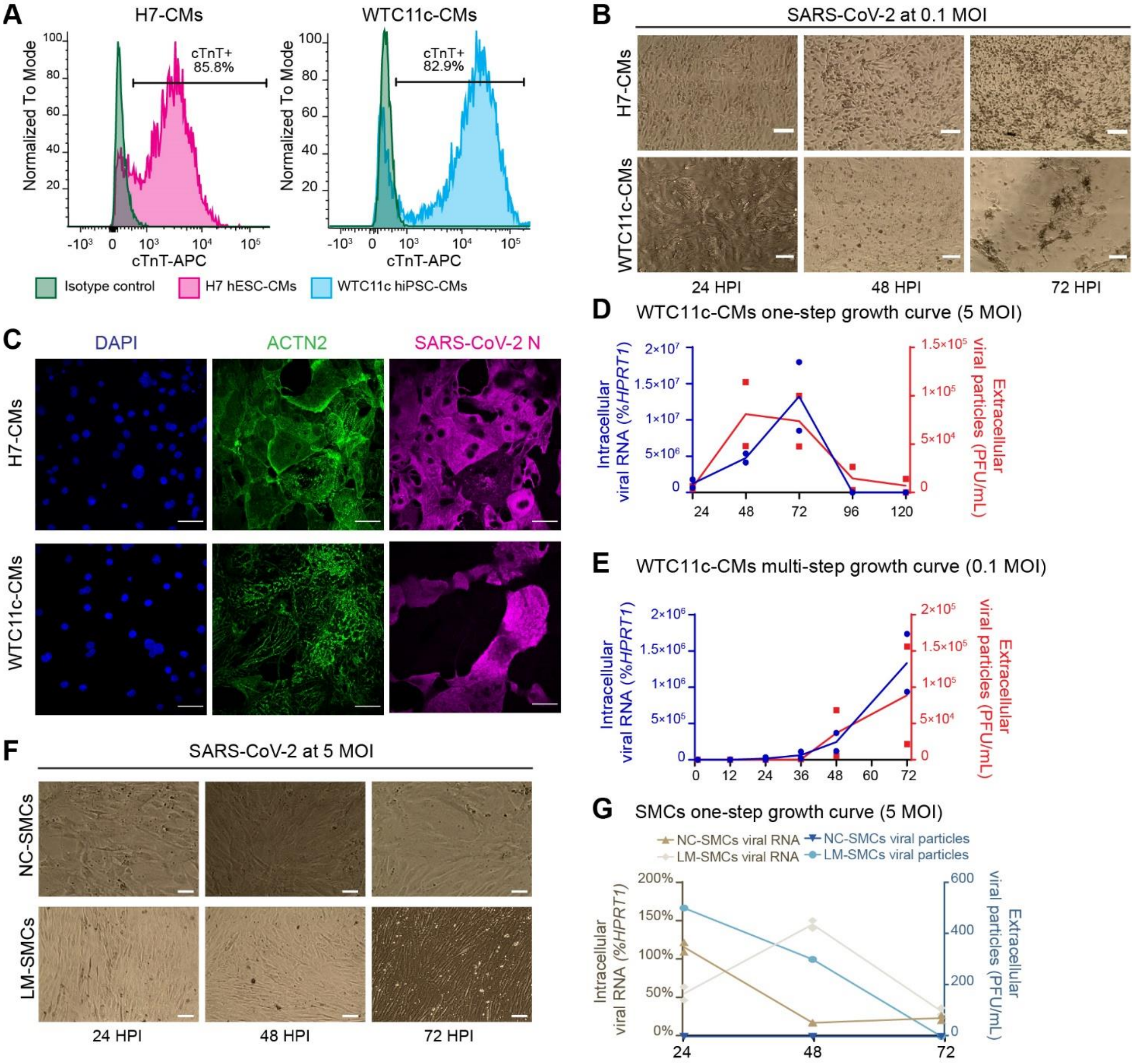

Supplementary Figure 2 – hPSC-CMs are permissive to SARS-CoV-2 infection and replication. (**A**) Representative flow cytometry analyses for cardiac troponin T (cTnT) expression in H7 hESC-CMs and WTC11c hiPSC-CMs. (**B**) Representative images of H7 hESC-CMs and WTC11c hiPSC-CMs infected with SARS-CoV-2 at 0.1 MOI during a time course of 72 h. Scale bars: 100 µm. Related to Figure 2A. (**C**) Single channel images for the immunostainings of SARS-CoV-2-infected hPSC-CMs shown in Figure 2B. (**D**) One-step viral growth curve in WTC11c hiPSC-CMs infected with SARS-CoV-2 at 5 MOI. (**E**) Multi-step viral growth curve in WTC11c hiPSC-CMs infected with SARS-CoV-2 at 0.1 MOI. For both D and E, lines connect the mean of two independent experiments. Viral RNA indicating intracellular viral replication is plotted on the left y axis as % of *HPRT1*. Viral particles secreted in the supernatant are plotted on the right y axis as PFU/mL. (**F**) Representative images of LM-SMCs and NC-SMCs infected with SARS-CoV-2 at 5 MOI during a time course of 72 h. Scale bars: 100 µm. (**G**) One-step viral growth curve in LM-SMCs and NC-SMCs infected with SARS-CoV-2 at 5 MOI. Viral RNA indicating intracellular viral replication is plotted on the left y axis as % of *HPRT1*. Plots as for panels D-E. Lines connect the means of two independent experiments.

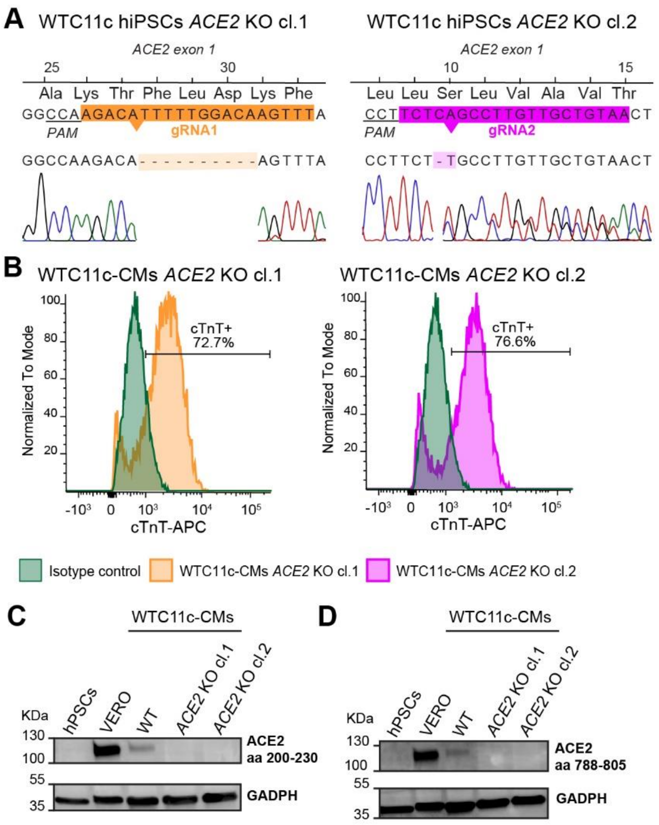

Supplementary Figure 3 – Genotyping and validation of WTC11c hiPSC *ACE2* knockout clones. Related to Figures 2E, F. (**A**) Genotyping by Sanger sequencing of WTC11c hiPSCs *ACE2* knockout (KO) clone 1 and clone 2. The location of sgRNAs protospacer sequences is highlighted. (**B**) Flow cytometry analyses of WTC11c-CMs *ACE2* KO clones. (**C-D**) Western blot for ACE2 N-terminal domain (C) and C-terminal domain (D) in WTC11c-CMs *ACE2* KO clones, confirming loss of full length ACE2 as well as of potential truncations (no bands detected at a lower molecular weight).

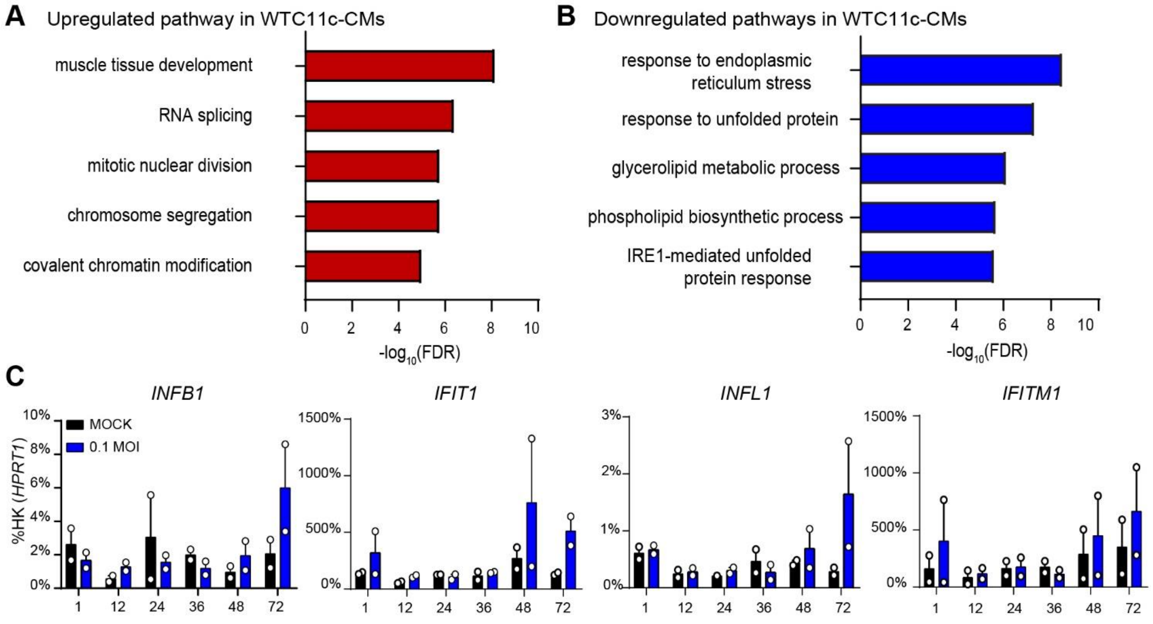

Supplementary Figure 4 – Gene expression analysis of WTC11c-CMs infected with SARS-CoV-2. (**A**) GO analysis of upregulated pathways in WTC11c-CMs infected with SARS-CoV-2 at 5 MOI after 48 HPI. (**B**) GO analysis of downregulated pathways in WTC11c-CMs infected with SARS-CoV-2 at 5 MOI after 48 HPI. (**C**) RT-qPCR of interferon response genes in WTC11c hiPSC-CMs infected with SARS-CoV-2 at 0.1 MOI. Mean ± SEM of 2 independent experiments.

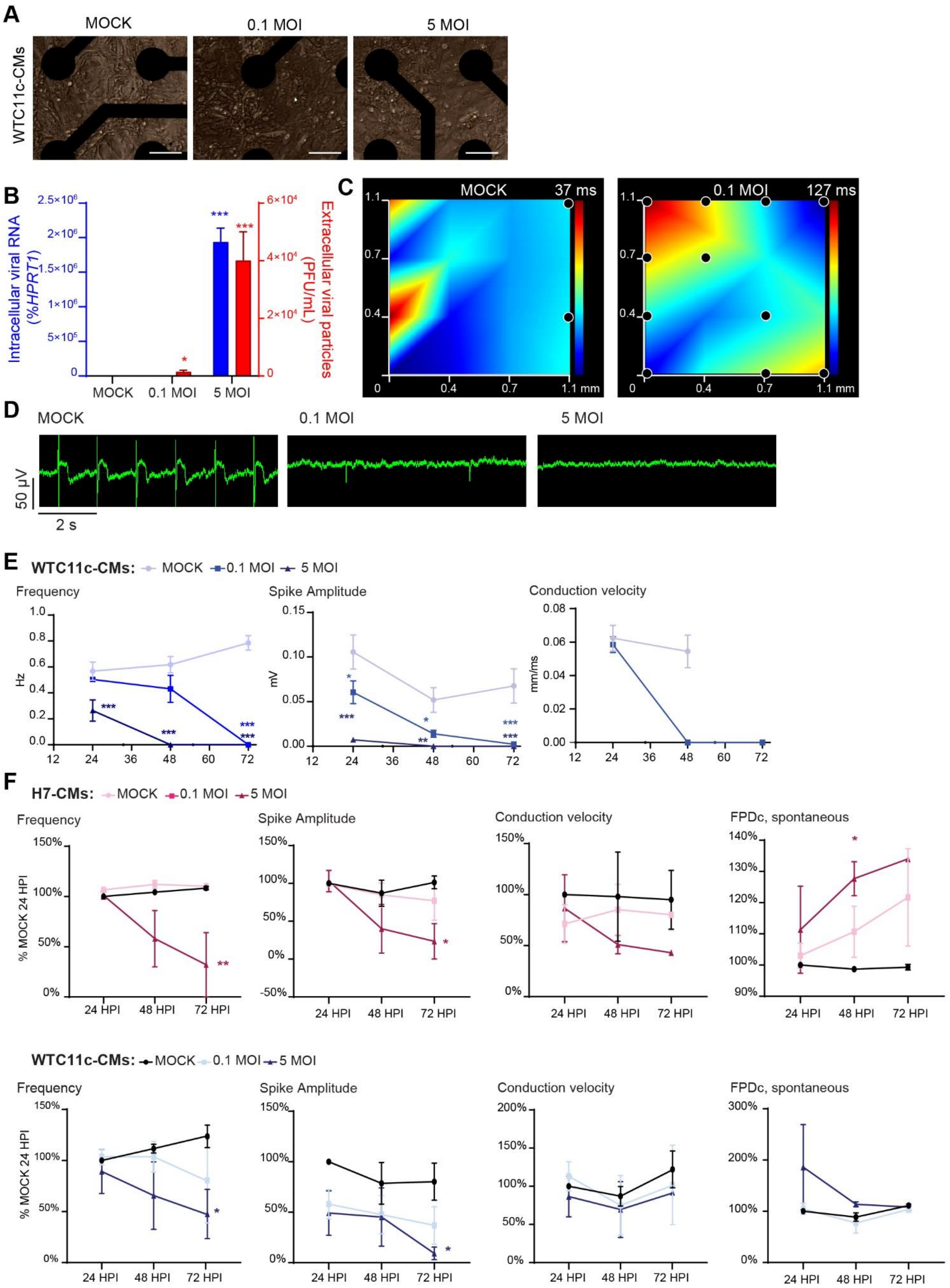

Supplementary Figure 5 – Electrophysiological alterations in hPSC-CMs infected with SARS-CoV-2. (**A**) Representative images of SARS-CoV-2-infected WTC11c hiPSC-CMs on MEA well at 72 HPI. Scale bars: 50 µm. (**B**) SARS-CoV-2 viral RNA and extracellular particles detected in WTC11c hiPSC-CMs in MEA plate. Viral RNA is plotted on the right axis as % *HPRT1*, extracellular viral particles are plotted on the left axis as PFU/mL. (**C**) Representative propagation maps of SARS-CoV-2-infected WTC11c hiPSC-CMs at 72 HPI. (**D**) Representative recording of spontaneous electrical activity of SARS-CoV-2-infected WTC11c hiPSC-CMs at 72 HPI. (**E**) Representative quantification of electrophysiological properties from MEA analyses on SARS-CoV-2-infected WTC11c hiPSC-CMs. Mean ± SEM of 8 wells. Differences versus mock calculated by two-way ANOVA with Sidak correction for multiple comparisons (* = $p < 0.05$; ** = $p < 0.01$; *** = $p < 0.001$). (**F**) Aggregated quantification of electrophysiological properties of SARS-CoV-2-infected hPSC-CMs. Data are shown as percentage of MOCK samples at 24 HPI. Mean ± SEM of 3 independent experiments. Statistical analyses as for panel E. Related to Figures 5E, F.

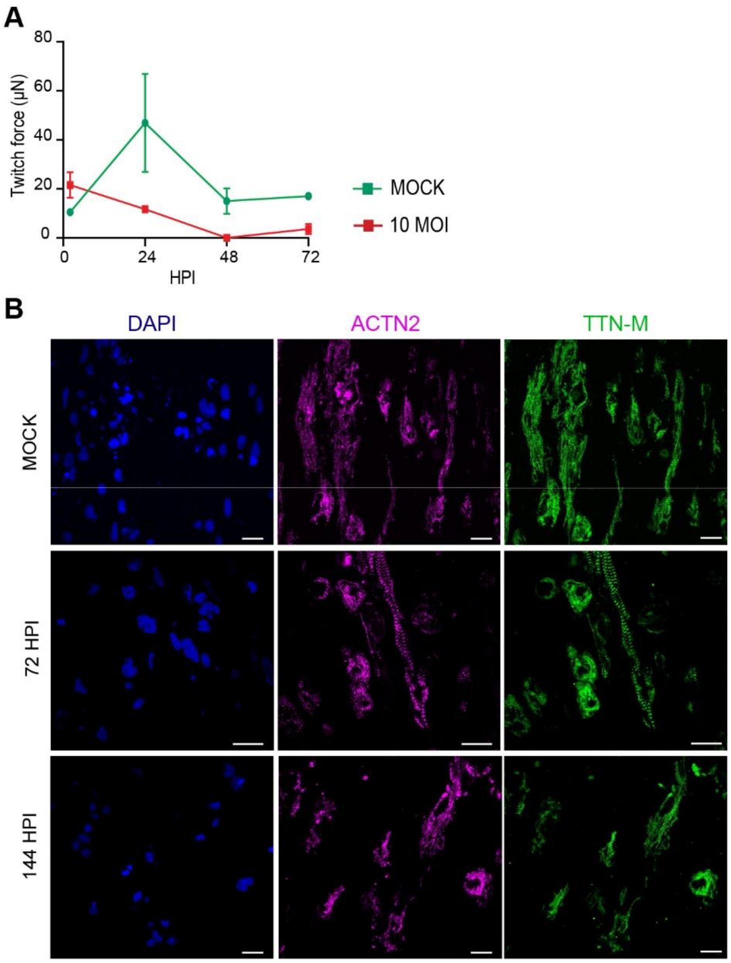

Supplementary Figure 6 - WTC11c hiPSC-CMs 3D-EHTs infected with SARS-CoV-2. (**A**) Additional example of a time course analysis of twitch force in 3D-EHTs from WTC11c hiPSC-CMs after SARS-CoV-2 infection at 10 MOI (see Fig. 6D). Data are shown as mean ± SEM for 2 mock controls and 4 infected 3D-EHTs. (**B**) Single channel images for the immunostainings of SARS-CoV-2-infected 3D-EHTs shown in Figure 6F.

Supplementary Table 1 – Extended data from GO analysis (upregulated pathway for H7-CMs)

H7-CMs 0.1 MOI at 48 HPI			
Ranking	**Pathway**	**FDR**	**-Log$_{10}$(FDR)**
**1**	Defense response to virus	1.76E-07	6.75448733
**2**	Mesenchymal cell differentiation	6.60E-07	6.18045606
**3**	Renal system development	6.75E-07	6.17091075
**4**	Kidney development	7.57E-07	6.12101888
**5**	Mesenchyme development	7.57E-07	6.12101888
**6**	Cardiac chamber development	1.89E-06	5.72452411
**7**	Urogenital system development	1.89E-06	5.72452411
**8**	Response to type I interferon	3.23E-06	5.4912459
**9**	Epithelial tube morphogenesis	3.23E-06	5.4912459
**10**	Cardiac septum development	5.44E-06	5.2644011
**11**	Mesenchymal cell development	5.44E-06	5.2644011
**12**	Regulation of cell morphogenesis	5.87E-06	5.23160859
**13**	Type I interferon signaling pathway	6.91E-06	5.16025268
**14**	Cellular response to type I interferon	6.91E-06	5.16025268
**15**	Connective tissue development	8.28E-06	5.08184627
H7-CMs 5 MOI at 48 HPI			
Ranking	**Pathway**	**FDR**	**-Log$_{10}$(FDR)**
1	*Histone modification*	*1.76E-23*	*22.7544873*
**2**	Covalent chromatin modification	1.14E-22	21.941574
3	*mRNA processing*	*2.85E-18*	*17.5458668*
**4**	Peptidyl-lysine modification	3.30E-15	14.4814861
**5**	RNA splicing	7.22E-15	14.1417035
**6**	Regulation of hemopoiesis	4.55E-12	11.3423069
**7**	Regulation of mRNA processing	3.81E-11	10.4186952
8	*Response to virus*	*3.81E-11*	*10.4186952*
**9**	Myeloid cell differentiation	3.81E-11	10.4186952
10	*Intracellular receptor signaling pathway*	*4.93E-11*	*10.3073293*
**11**	Regulation of RNA splicing	1.44E-10	9.84163751
12	*Defense response to virus*	*1.56E-10*	*9.80773284*
**13**	Nuclear export	1.56E-10	9.80773284
**14**	Histone acetylation	2.95E-10	9.52954751
**15**	Mesenchyme development	3.64E-10	9.4392169

The pathways in italic are the ones plotted in Fig. 4E.

Supplementary Table 2 – Extended data from GO analysis (downregulated pathway for H7-CMs)

H7-CMs 0.1 MOI at 48 HPI			
Ranking	**Pathway**	**FDR**	**-Log$_{10}$(FDR)**
**1**	ATP synthesis coupled electron transport	2.64E-27	26.57840
**2**	Cellular respiration	2.64E-27	26.57840
**3**	Mitochondrial ATP synthesis coupled electron transport	2.64E-27	26.57840
**4**	Mitochondrion organization	2.64E-27	26.57840
**5**	Respiratory electron transport chain	4.58E-27	26.33913
**6**	Mitochondrial respiratory chain complex assembly	6.16E-26	25.21042
**7**	Oxidative phosphorylation	1.06E-25	24.97469
**8**	mRNA processing	2.75E-25	24.56067
**9**	RNA splicing	1.86E-23	22.73049
**10**	ATP metabolic process	3.68E-23	22.43415
**11**	Electron transport chain	3.68E-23	22.43415
**12**	Ribonucleoprotein complex biogenesis	4.55E-22	21.34199
**13**	Ribonucleoside monophosphate metabolic process	5.01E-22	21.30016
**14**	Purine nucleoside triphosphate metabolic process	2.07E-21	20.68403
**15**	Purine ribonucleoside monophosphate metabolic process	2.85E-21	20.54516
H7-CMs 5 MOI at 48 HPI			
Ranking	**Pathway**	**FDR**	**-Log$_{10}$(FDR)**
1	*Cellular respiration*	*2.93E-27*	*26.53264*
**2**	Mitochondrial respiratory chain complex assembly	2.93E-27	26.53264
**3**	ATP synthesis coupled electron transport	2.93E-27	26.53264
**4**	Mitochondrial ATP synthesis coupled electron transport	5.06E-27	26.29585
5	*Oxidative phosphorylation*	*8.45E-27*	*26.07325*
**6**	Respiratory electron transport chain	2.20E-26	25.65758
**7**	Electron transport chain	1.63E-25	24.78667
8	*Generation of precursor metabolites and energy*	*2.42E-23*	*22.61618*
**9**	NADH dehydrogenase complex assembly	1.50E-22	21.82507
**10**	Mitochondrial respiratory chain complex I assembly	1.50E-22	21.82507
**11**	Energy derivation by oxidation of organic compounds	8.80E-22	21.05552
**12**	Mitochondrial electron transport, NADH to ubiquinone	6.75E-21	20.17091
13	*Small molecule catabolic process*	*6.77E-20*	*19.16946*
14	*Purine nucleoside triphosphate metabolic process*	*4.02E-19*	*18.39547*
**15**	Purine ribonucleotide metabolic process	8.21E-19	18.08548

The pathways in italic are the ones plotted in Fig. 4F.

Supplementary Table 3 – Extended data from GO analysis (upregulated pathway for WTC11c-CMs)

WTC11c-CMs 0.1 MOI at 48 HPI			
Ranking	**Pathway**	**FDR**	**$-Log_{10}(FDR)$**
**1**	RNA splicing	8.80E-07	6.05551733
**2**	Double-strand break repair	1.06E-05	4.97633608
**3**	Sister chromatid segregation	3.52E-05	4.45345734
**4**	mRNA processing	4.84E-05	4.31515464
**5**	RNA splicing, via transesterification reactions	6.91E-05	4.16025268
**6**	RNA splicing, via transesterification reactions with bulged adenosine as nucleophile	6.91E-05	4.16025268
**7**	mRNA splicing, via spliceosome	6.91E-05	4.16025268
**8**	Mitotic nuclear division	0.000165	3.78251606
**9**	Chromosome segregation	0.00025422	3.59478649
**10**	Nuclear division	0.000264	3.57839607
**11**	Mitotic sister chromatid segregation	0.000264	3.57839607
**12**	Organelle fission	0.00052067	3.28344023
**13**	Nuclear chromosome segregation	0.00052123	3.28296995
**14**	Double-strand break repair via homologous recombination	0.00075429	3.12246412
**15**	Recombinational repair	0.000825	3.08354605
WTC11c-CMs 5 MOI at 48 HPI			
Ranking	**Pathway**	**FDR**	**$-Log_{10}(FDR)$**
**1**	Muscle organ development	2.11E-12	11.6753061
2	*Muscle tissue development*	*7.04E-09*	*8.15242734*
**3**	Striated muscle tissue development	9.68E-09	8.01412464
**4**	Sister chromatid segregation	3.96E-07	6.40230481
5	*RNA splicing*	*3.96E-07*	*6.40230481*
**6**	Reproductive system development	3.96E-07	6.40230481
**7**	Reproductive structure development	8.93E-07	6.04935702
8	*Mitotic nuclear division*	*1.66E-06*	*5.77931092*
9	*Chromosome segregation*	*1.66E-06*	*5.77931092*
**10**	Rhythmic process	1.67E-06	5.77676373
**11**	Heart morphogenesis	5.04E-06	5.29756946
**12**	Mitotic sister chromatid segregation	5.65E-06	5.24820785
**13**	Peptidyl-lysine modification	8.80E-06	5.05551733
14	*Covalent chromatin modification*	*9.97E-06*	*5.00115967*
**15**	Nuclear chromosome segregation	9.97E-06	5.00115967

The pathways in italic are the ones plotted in Supplementary Fig. 4A.

Supplementary Table 4 – Extended data from GO analysis (downregulated pathway for WTC11c-CMs)

WTC11c-CMs 0.1 MOI at 48 HPI			
Ranking	**Pathway**	**FDR**	**-Log$_{10}$(FDR)**
**1**	Glycerolipid metabolic process	8.80E-06	5.05551733
**2**	Glycoprotein metabolic process	1.76E-05	4.75448733
**3**	Response to endoplasmic reticulum stress	3.81E-05	4.41869523
**4**	Glycerolipid biosynthetic process	7.54E-05	4.12246412
**5**	Glycerophospholipid metabolic process	7.54E-05	4.12246412
**6**	Urogenital system development	7.54E-05	4.12246412
**7**	Protein localization to plasma membrane	7.54E-05	4.12246412
**8**	Glycoprotein biosynthetic process	0.00012711	3.89581648
**9**	Renal system development	0.00012711	3.89581648
**10**	Fat cell differentiation	0.000184	3.73518218
**11**	Kidney development	0.000184	3.73518218
**12**	Phospholipid metabolic process	0.000352	3.45345734
**13**	Membrane lipid metabolic process	0.00037231	3.42909799
**14**	Glycolipid metabolic process	0.00054686	3.26212611
**15**	Liposaccharide metabolic process	0.00064533	3.1902159
WTC11c-CMs 5 MOI at 48 HPI			
Ranking	**Pathway**	**FDR**	**-Log$_{10}$(FDR)**
1	*Response to endoplasmic reticulum stress*	*3.26E-09*	*8.4873156*
2	*Response to unfolded protein*	*4.84E-08*	*7.31515464*
**3**	Response to topologically incorrect protein	1.03E-07	6.98857054
**4**	Small molecule catabolic process	1.21E-07	6.91721463
**5**	Glycerophospholipid metabolic process	2.99E-07	6.52403841
**6**	Lipid modification	4.25E-07	6.37127058
7	*Glycerolipid metabolic process*	*7.42E-07*	*6.12976336*
**8**	Organic acid catabolic process	9.68E-07	6.01412464
**9**	Carboxylic acid catabolic process	9.68E-07	6.01412464
**10**	Glycoprotein metabolic process	9.68E-07	6.01412464
**11**	Protein folding	1.28E-06	5.89279003
**12**	Glycosylation	1.83E-06	5.73675857
13	*Phospholipid biosynthetic process*	*2.03E-06*	*5.69233943*
14	*IRE1-mediated unfolded protein response*	*2.33E-06*	*5.63344364*
**15**	Glycoprotein biosynthetic process	4.84E-06	5.31515464

The pathways in italic are the ones plotted in Supplementary Fig. 4B.

Supplementary Table 5 – RT-qPCR primer sequences used in this study

Target	Forward primer (5' – 3')	Reverse primer (5' – 3')
HPRT1	TGACACTGGCAAAACAATGCA	GGTCCTTTTCACCAGCAAGCT
NANOG	TTTGTGGGCCTGAAGAAAACT	AGGGCTGTCCTGAATAAGCAG
TBXT	CAAATCCTCATCCTCAGTTTG	GTCAGAATAGGTTGGAGAATTG
PDGFRA	GCTCACTTCACTCTCCCCAAAG	CCGGCGTTCCTGGTCTTAG
TNNT2	TTCACCAAAGATCTGCTCCTCGCT	TTATTACTGGTGTGGAGTGGGTGTGG
ACE2	CCATCAGGATGTCCCGGAG	TGGAGGCATAAGGATTTTCTCCA
SARS-CoV-2_E	GAACCGACGACGACTACTAGC	ATTGCAGCAGTACGCACACA
IFIT1	AGAAGCAGGCAATCACAGAAAA	CTGAAACCGACCATAGTGGAAAT
IFITM1	TACTCCGTGAAGTCTAGGGACAG	AACAGGATGAATCCAATGGTCA
IFNB	CTTGGATTCCTACAAAGAAGCAGC	TCCTCCTTCTGGAACTGCTGCA
IFNL	AACTGGGAAGGGCTGCCACATT	GGAAGACAGGAGAGCTGCAACT
MYL2	TTGGGCGAGTGAACGTGAAAA	CCGAACGTAATCAGCCTTCAG
MYH6	GCCCTTTGACATTCGCACTG	GGTTTCAGCAATGACCTTGCC

Supplemental Experimental Procedures

Cell culture. Undifferentiated RUES2 hESCs (RUESe002-A; WiCell) and WTC11c hiPSCs (a gift of Dr. Bruce Conklin, Gladstone Institutes, San Francisco) were maintained in mTeSR1 (Stemcell Technologies) on tissue culture dishes coated with Matrigel (Corning) at 0.17 mg/mL, and passaged as small clumps using Versene (Gibco). Cardiomyocytes from RUES2 and WTC11c were differentiated as previously described (Bertero et al., 2019). Briefly, undifferentiated cells were seeded at 1,000 cells/cm^2 in mTeSR1 supplemented with 10 µM Y-27632 (Tocris) on Matrigel-coated dishes. After 24 h media was changed to mTeSR1 with 1 µM CHIR-99021 (Cayman). On day 0 mesoderm differentiation was induced with 3 µM CHIR-99021 in RPMI-1640 media (ThermoFisher) supplemented with 500 µg/mL BSA (Sigma-Aldrich) and 213 µg/mL ascorbic acid (Sigma-Aldrich), denoted as RBA media. On day 2 media was changed to RBA containing 2 µM WNTC59 (Selleckchem). On day 4 media was changed to plain RBA. On day 6 media was changed to RPMI-1640 plus B-27 supplement (ThermoFisher), with further media changes every other day. Heat-shock was performed on day 13 for 30 min at 42 °C, and on day 14 cardiomyocytes were dissociated and frozen in CS10 cryopreservation media (Sigma-Aldrich). H7 hESCs (WA07; WiCell) were differentiated in suspension culture format by collaborators at the Center for Applied Technology Development at the City of Hope in California (as previously described (Chen et al., 2015), received by dry-ice shipment, and stored in liquid nitrogen before use.

Undifferentiated H9 hESCs (WA09; WiCell) were maintained and differentiated into LM-SMCs and NC-SMCs as previously described (Bargehr et al., 2016; Serrano et al., 2019). In brief, early mesoderm differentiation was commenced in chemically defined medium with polyvinyl alcohol (CDM-PVA) supplemented with FGF2 (20 ng/mL), LY294002 (10 mM), and BMP4 (10 ng/mL) for 1.5 days. Subsequently, lateral mesoderm differentiation was started in CDM-PVA with FGF2 (20 ng/mL) and BMP4 (50 ng/mL) for 3.5 days. For SMC differentiation, LM cells were resuspended as single cells in CDM-PVA supplemented with PDGF-BB (10 ng/mL) and TGF- β1 (2 ng/mL) for 12 days with media change every other day. To generate NC-SMCs, the NC intermediate was produced by culturing H9 hESCs in CDM-PVA with FGF2 (12 ng/mL) and SB431542 (10 µM; Tocris), which were then split into single cells after 4 days. NC cells were cultured with daily media changes and passaged as needed. After passage 5, SMC induction was initiated with the addition of CDM-PVA with PDGF-BB (10ng/mL) and TGF-β1 (2 ng/mL) for 12 days with media changes every other day. Both LM- and NC-SMCs were subsequently maintained in DMEM F12 (Gibco) supplemented with 10% FBS for 7 days prior to freezing. Following their generation in Cambridge, UK, cells were shipped in liquid nitrogen to Seattle, WA, USA.

Gene-editing for *ACE2* KO cell line and clonal isolation. WTC11c hiPSCs were electroporated with CRISPR/Cas9 ribonucleoprotein complexes targeting exon 1 of ACE2 (Liang et al., 2015). Briefly, a mixture of 60 pmol of *Sp*Cas9 2xNLS nuclease (Synthego) and 60 pmol of single guide RNA mix (protospacers 5'-TTTGAACAGGTTTTTACAG-3'; 5'-AATGTCGTTGTTCCGACTCT-3' and 5'-CTGTAAGAGAAGTCATTATA-3'; Synthego) were incubated in 300 µL of Neon Buffer R (ThermoFisher) at room temperature for 1 h. 500,000 WTC11c hiPSCs were resuspended in the ribonucleoprotein mixture, electroporated (1 pulse at 1,300 V for 30 ms), and immediately replated on matrigel-coated 6-well plate in mTeSR1 supplemented with 10 µM Y-27632. Clonal isolation was performed through limiting dilution during passaging. Briefly, the targeted WTC11c hiPSC pool was harvested as single-cell suspension using 0.75X TrypLE in Versene at 37 °C for 3 min, collected with mTeSR1 supplemented with 10 µM Y-27632, serially diluted to reach a density of 5 cell/mL, and seeded onto 96-well plates at a density of 0.5 cell/well. Single cell-derived clones were subsequently maintained and differentiated in cardiomyocytes as described above. Standard G-banding analysis was performed on undifferentiated cells to confirm absence of karyotype abnormalities (Diagnostic Cytogenetics, Seattle WA).

Genotyping. Genomic DNA (gDNA) was isolated from *ACE2* KO clones with the DNeasy Blood & Tissue kit (QIAGEN) according to manufacturer's instructions. 500 ng of gDNA was amplified using Q5 High Fidelity 2X Master Mix (NEB) and 5 µM of forward and reverse primers (5'- GCCATAAAGTGACAGGAGAG-3' and 5'-GAAATCCTGACTGTGATGAG-3'). PCR products were purified using the QIAquick PCR purification kit (QIAGEN) and analyzed by Sanger sequencing (sequencing primer: 5'TTTGATTTCCCTTTTCAGTT-3').

Flow cytometry. Cardiac differentiation efficiency was determined by flow cytometry in hPSC-CMs fixed with 4% paraformaldehyde for 15 min at room temperature. Cells were centrifuged at 300 g for 5 min and incubated at room temperature for 1 h with either APC-cTnT antibody (Miltenyi Biotech #130-120-543) or APC-IgG1 isotype control antibody (Miltenyi Biotech #130-120-709), both used at 1:100 dilution in DPBS (Gibco) with 5% fetal bovine serum (FBS) and 0.75% saponin. Following washes with DPBS with 5% FBS, samples were run on a BD FACSCanto II flow cytometer and data from 10,000 valid events were acquired with the BD FACSDIVA software. Analysis was performed with FlowJo v10.7.

RNA-seq. Bulk RNA-seq datasets from differentiating RUES2 hESC-CMs had been previously generated and analyzed (Bertero et al., 2019) (GEO dataset: GSE106688). Bulk mRNA-seq data from infected cardiomyocytes were generated by constructing mRNA-seq libraries using the KAPA mRNA HyperPrep Kit (Kapa Biosystems) and 100 ng of RNA. Both the RNA and resulting libraries were quality controlled and quantified using a 4200 TapeStation System (Agilent Technologies, Inc.) and a Qubit Fluorometer (Invitrogen). Libraries were sequenced on an Illumina NovaSeq using S2 200 cycle flow cells which generates paired-end reads of 100 nucleotides. Each sample was sequenced to a minimum of 20 million mapped reads. Read quality for each sample was first assessed with FASTQC (Andrews, 2010) and then adapters and bases with phred quality score below 20 were removed from the end of the reads with TrimGalore (Martin, Cutadapt Removes Adapter

Sequences From High-Throughput Sequencing Reads). Globin and rRNA reads were filtered out using bowtie2 v2.1.0 (Langmead and Salzberg, 2012), and remaining reads were mapped to the hg19 genome using STARv2.7.5a (Dobin et al., 2013). Read counts for each gene were quantified using HTSeqv0.12.4 (Anders et al., 2015). We also mapped reads to the SARS-CoV-2 genome (GCF_009858895.2) using bowtie2v2.1.0 to quantify how many of the reads were derived from virus. Genes with a read count average of less than 10 across all samples were removed. Read counts were normalized using TMM normalization and differentially expressed (DE) genes were determined using the EdgeR and limma Bioconductor packages (Ritchie et al., 2015; Robinson et al., 2010; Robinson, 2010). Significant DE genes had to have an adjusted p-value of less than 0.05 and an absolute fold change greater than 1.5. TopGO R package with Fisher's Exact Test was used for Gene Ontology enrichment analysis (Alexa et al., 2006).

Western blot. Cell pellets were incubated in RIPA Buffer supplemented with 1X Protease inhibitors (ThermoFisher) at 4 °C for 20 min. Samples were then centrifuged at 21,000 g for 15 min at 4 °C, and protein concentration in the supernatant was quantified with BCAssay (ThermoFisher). 25 µg of protein were mixed with 1X non-reducing SDS sample buffer and incubated at 37 °C for 30 min. Samples were run on 4-20% mini-PROTEAN TGX precast gels (Bio-Rad) and then transferred on PVDF membranes. Membranes were incubated with 5% non-fat dry milk in TBS buffer supplemented with 0.1% Tween-20 (blocking buffer) for 1 h at room temperature. Primary antibodies were incubated in blocking buffer for 2 h at room temperature (rabbit anti-ACE2 C-terminal domain [Abcam #ab15348, used at 1:500 dilution], rabbit anti-ACE2 N-terminal domain [Novusbio #SN0754, used at 1:500 dilution], mouse anti-cTnT [ThermoFisher #MA5-12960 used at 1:200 dilution], and mouse anti-GAPDH [Abcam #ab8245, used at 1:3,000 dilution]). Membranes were washed and further incubated with fluorescent dye-conjugated secondary antibodies for 1 h at room temperature (AlexaFluor 647 goat anti-rabbit IgG1 and AlexaFluor 488 goat anti-mouse IgG1, both used at 1:1,000 dilution in blocking buffer) and fluorescent signals were acquired using with a GelDoc Imager (Bio-Rad). Quantification of bands intensity was performed using Fiji software.

Single cell RNA-seq. A single cell suspension was generated from cultures of RUES2 hESC-CMs at day 30 of differentiation, and single cell RNA-seq was performed using the Chromium NextGEM Single Cell 3' kit (10X Genomics). 10,000 cells per condition were loaded on independent microfluidics channels to generate Gel bead-in-Emulsion (GEMs), which were further processed according to the manufacturer's instructions to generate Illumina-compatible sequencing libraries. The sample was analyzed using two runs of high output NextSeq 500 with a 150 cycle kit, reading 28 base pairs for read 1 (barcode and UMI), 91 base pairs for read 2 (3' end of cDNAs), and 7 base pairs for the i7 index. Initial data analysis relied on the cellranger package from 10X Genomics. Cellranger mkfastq was used to transfer demultiplexed raw base call files into library-specific FASTQ files. The FASTQ files were separately mapped to the GRC38 human reference genome using STAR as a part of the cellranger pipeline. Gene expression counts were done using cellranger count based on Gencode v25 annotation, and cell identifiers and Unique Molecular Identifiers (UMI) were filtered and corrected with default setting. Raw cellranger count outputs was aggregated and visualized by a subsampling procedure using cellranger aggr. Downstream analysis was performed in the R package Seurat (Butler et al., 2018; Stuart et al., 2019). Filters were applied to eliminate cells with less than 1,000 genes detected, with over 40,000 UMIs, or with over 35% mitochondrial gene reads. Post filtering, cell-to-cell gene expression was normalized by total expression, multiplied by the scale factor of 10000, and the result is log-transformed [Log norm exp]). Data dimensionality reduction was done by principal component analysis on the top 2,000 most variable genes. The top 10 principal components (PCs) that explained most variance were selected, as confirmed by an Elbow plot. For visualization, UMAP dimensionality reduction for the top 10 PCs was performed to produce coordinates for cells in 2 dimensional space. The relative expression levels of genes of interest were plotted using the FeaturePlot function in Seurat.

SARS-CoV-2 generation. All experiments using live virus were performed in the Biosafety Level 3 (BSL-3) facility at the University of Washington in compliance with the BSL-3 laboratory safety protocols (CDC BMBL 5th ed.) and the recent CDC guidelines for handling SARS-CoV-2. Before removing samples from BSL-3 containment, samples were inactivated by Trizol or 4% paraformaldehyde, and the absence of viable SARS-CoV-2 was confirmed for each sample by plaque assays. SARS-Related Coronavirus 2, Isolate USA-WA1/2020 (SARS-CoV-2) and icSARS-CoV-2mNG were obtained from BEI Resources (NR-52281) and the University of Texas (Xie et al., 2020), respectively and propagated in VERO cells (USAMRIID). Briefly, VERO cells were maintained in DMEM (Gibco) supplemented with 10% heat-inactivated FBS, 100 U/mL penicillin, and 100 U/mL streptomycin at 37 °C in a 5% CO_2 humidified incubator. To generate virus stock, cells were washed once with DPBS and infected with SARS-CoV-2 in serum-free DMEM. After 1 h of virus adsorption, the inoculum was replaced with DMEM supplemented with 2% heat-inactivated FBS, and cells were incubated at 37 °C in a 5% CO_2 incubator until ~70% of cells manifested cytopathic effects. The virus was harvested by collecting the culture supernatant followed by centrifugation at 3,000 g for 15 min at 4 °C to remove the cell debris. Virus titer was then measured by plaque assay on VERO cells (as described below), and stocks were stored at -80°C.

SARS-CoV-2 titering. Viral preparations and culture supernatant from SARS-CoV-2-infected cardiomyocytes were titered using a plaque assay. Briefly, 350,000 VERO cells were seeded in 12-well plates and incubated for 1 h at 37 °C with 10-fold dilutions of virus-containing media. A solution of 1:1 1% agarose and 2X DMEM supplemented with 4% heat-inactivated FBS, L-glutamine, 1X antibiotic-antimycotic (Gibco), and 220 mg/mL sodium pyruvate was layered on top of the cells, followed by incubation at 37 °C for 2 days. After fixing with 10% formaldehyde, the agarose layer was removed and cells

were stained with 0.5% crystal violet solution in 20% ethanol. Plaques were counted, and the virus titer in the original sample was assessed as plaque-formation unit per mL (PFU/mL).

Viral infection. Cryopreserved hPSC-CMs were thawed and plated in RPMI-1640 supplemented with B-27, 5% FBS, and 10 µM Y-27632. After 24 h the media was replaced with RPMI-1640 supplemented with B-27 only. After 3 days, cardiomyocytes were harvested with Versene supplemented with 0.5% Trypsin (Gibco) at 37 °C for 5 min to obtain single-cell suspensions. 250,000 cardiomyocytes were seeded in Matrigel-coated 12-well plates in RPMI-1640 supplemented with B-27, 5% FBS, and 10 µM Y-27632. The media was replaced with RPMI-1640 supplemented with B-27 the next day, and then every other day for 1 week. SARS-CoV-2 wild-type or expressing Neon green protein was diluted to the desired MOI in DMEM and incubated on hPSC-CMs for 1 h at 37 °C (non-infected [mock] controls were incubated with DMEM only). Cells were then washed with DPBS and cultured in RPMI-1640 supplemented with B-27. Cryopreserved hPSC-SMCs were thawed in DMEM F12 supplemented with 10% FBS and 10 µM Y-27632. After 24 h, the media was replaced with DMEM F12 supplemented with 10% FBS only. After 4 days, smooth muscle cells were harvested with 0.5% Trypsin (Gibco) at 37 °C for 5 min to obtain single-cell suspensions. 100,000 smooth muscles cells were seeded in gelatin-coated 12-well plates in DMEM F12 supplemented with 10% FBS and 10 µM Y-27632. The media was replaced after 24 h with fresh DMEM F12 with 10% FBS. After 5 days from replating, hPSC-SMCs were incubated with SARS-CoV-2 diluted to the desired MOI in DMEM for 1 h at 37 °C (non-infected [mock] controls were incubated with DMEM only). Cells were then washed with DPBS and cultured in DMEM F12 supplemented with 10% FBS.

Immunofluorescence. 200,000 cardiomyocytes were plated on glass-bottom 24-well plate (CellVis) and infected as described above. Cells were fixed with 4% paraformaldehyde in DPBS for 30 min at room temperature and then washed 3 times with DPBS for 5 min. Cells were permeabilized using 0.25% Triton X-100 (Sigma-Aldrich) in DPBS and blocked for 1 h with 10% normal goat serum supplemented with 0.1% Tween-20 in DPBS. Primary antibodies were incubated overnight at 4 °C in DPBS with 1% normal goat serum and 0.1% Tween-20 (rabbit anti-2019-nCoV NP [Sino Biological #40143-R019, used at 1:200 dilution], and mouse anti-Sarcomeric α-actinin [Abcam ab# ab9465, used at 1:500 dilution]). Cell were washed three times with DPBS containing 0.2% Tween-20, and incubated for 1 h at room temperature with secondary antibodies diluted in DPBS supplemented with 1% BSA and 0.1% Tween-20 (AlexaFluor 594 goat anti-rabbit IgG1 and AlexaFluor 647 goat anti-mouse IgG1, both used at 1:1,000 dilution). DAPI (Sigma-Aldrich) was diluted at 300 nM in water and incubated on the cells for 15 min at room temperature, followed by three washes in DPBS containing 0.2% Tween-20. Images were taken with a 40x oil objective on a Nikon Eclipse microscope with Yokogawa W1 spinning disk head, and formatted with Fiji software.

Electron Microscopy. 200,000 cardiomyocytes were plated on 35-mm petri dish (Corning) and infected as described above. Cells were fixed with Karnovsky's fixative for 30 min at room temperature and then washed 3 times with 0.1 M PIPES buffer, for 5 min. Heavy metal impregnation was performed as detailed elsewhere (Deerinck, 2010). The embedded cellular layer was cut into small pieces and re-embedded in en-face and perpendicular orientations in Durcopan (EMS). Thin sections were viewed using a JEOL 1230 transmission electron microscope.

Gene expression analysis and viral RNA detection. Infected cardiomyocytes were washed once with DPBS and incubated with 400 µL per well of Trizol reagent (Invitrogen) for 10 min at room temperature. Chloroform was added in a 5:1 ratio to Trizol, and samples were incubated at room temperature for 2 min. The aqueous phase was separated by centrifugation (21,000 g for 15 min at 4 °C) and incubated with isopropanol (1:1 ratio) and 25 µg of Glycoblue (ThermoFisher) for 10 min at room temperature. RNA pellets were harvested by centrifugation (21,000 g for 15 min at 4 °C), washed twice with 75% ethanol, and resuspended in nuclease-free water. cDNA was prepared with M-MLV reverse transcriptase according to the manufacturers' instruction. Quantitative real-time reverse transcription PCR (RT-qPCR) was performed with SYBR Select Master Mix (Applied Biosystems) using 10 ng of cDNA and 400 nM forward and reverse primers (Supplementary Table 5). Reactions were run on a CFX384 Real-Time System (Bio-Rad), and data was analyzed using the ΔΔCt method using *HPRT1* as the housekeeping gene. Primers were designed using PrimerBlast, and confirmed to amplify a single product.

Electrophysiological analysis with MEA. Cryopreserved cardiomyocytes were thawed and cultured as described above. CytoView MEA 48- and 24-well plates (Axion BioSystems) were coated with 0.17 mg/mL of Matrigel for 1 h at 37 °C. 50,000 (48-well plate) or 100,000 (24-well plate) hPSC-CMs were resuspended in 6 µL or 10 µL, respectively, and plated on each MEA well, as previously described (Hayes et al., 2019). Media was changed with RPMI-1640 supplemented with B-27 every other day for 1 week. One the day of viral infection, cells were washed once with DPBS and incubated with 50 µL (48-well plate) or 100 µL (24-well plate) of SARS-CoV-2 suspension for 1 h at 37 °C. Media was replaced with RPMI-1640 supplemented with B-27, and the plate was transferred directly into Maestro Pro system (Axion BioSystems) and kept at 37 °C with 5% CO_2 for the duration of the experiment. Electrophysiological recordings were taken for 5 min at specified time points using Axis software version 2.0.4. (Axion BioSystems). Voltage was acquired simultaneously for all the electrodes at 12.5 kHz, with a low-pass digital filter of 2 kHz for noise reduction. The beat detection threshold was 100 µV, and the FPD detection used a polynomial regression algorithm with the threshold set at 1.5 × noise to detect repolarization waves. Pacing was performed at 2 Hz with an alternating square wave (± 1 V, 100 nA, 8.33 kHz) delivered through the dedicated stimulator in the Maestro Pro system to a selected electrode (not used for recording). Automated analysis was performed using Cardiac

Analysis Software v3.1.8 (Axion BioSystems), which automatically computes the Fridericia correction to account for beat rate variability during FPD measurements [FPDc = FPD/(beat period)$^{1/3}$].

Contractility analysis with 3D-EHTs. 3D-EHTs were generated from hPSC-CMs embedded with stromal cells in a 3D fibrin gel suspended between pairs of silicone posts, as previously described (Bielawski et al., 2016). For each pair of silicone posts one was flexible and had a 1 mm^3 magnet embedded in its tip, and the other post was rendered rigid by embedding a 1.1 mm glass capillary tube. Each 3D-EHT was casted in a mold made of 2% agarose by adding 500,000 WTC11c hiPSC-CMs and 50,000 HS27a stromal cells (ATCC) in a fibrin gel solution (89 µL RPMI-1640 supplemented with B-27, 5.5 µl of DMEM/F12, [Gibco], 2.5 µL of 200 mg/mL bovine fibrinogen [Sigma-Aldrich], and 3 µL of 100 U/mL thrombin [Sigma-Aldrich]). The cell-gel mixture was incubated at 37 °C for 2 h to allow for fibrin polymerization. Afterwards, 3D-EHTs were transferred from the agarose molds to 24-well tissue culture dishes containing 3D-EHT media (RPMI-1640 media supplemented with B-27 and 5 mg/mL aminocaproic acid [Sigma-Aldrich]. Media was changed every other day for 2 weeks. For SARS-CoV-2 infection, 3D-EHTs were temporarily housed in 2% agarose molds, and 200 µL of viral solution in DMEM was used to infect each single tissue for 1 h at 37 °C (DMEM was used for mock controls). 3D-EHTs were then transferred in fresh 3D-EHT media for the rest of the experiment. Twitch force was recorded by tracking the movement of magnets embedded in the flexible posts, as previously described (Bielawski et al., 2016). Briefly, we used a custom-built printed circuit board (PCB) containing giant magnetoresistive (GMR) sensors (NVE, Eden Prairie, MN) in a Wheatstone bridge configuration and relying on instrumentation amplifiers and operational amplifiers to filter out signal noise. 3D-EHTs in the 24-well dish were placed into a 3D-printed caddy that contained the PCB with GMR sensors such that the flexible, magnetic posts of 3D-EHTs were directly above each GMR sensor. Data from the PCB was collected by LabView (National Instruments) on a laptop in the BSL-3 facility. The voltage traces from the magnetic sensors were analyzed for amplitude and frequency using a custom Matlab protocol. The amplitudes were then converted from voltage to twitch force using a characterization constant.

3D-EHTs immunostaining. At the indicated time points, 3D-EHTs were harvested for immunofluorescence. 3D-EHTs were treated with 150 mM KCl to arrest contraction in diastole, and fixed in 4% paraformaldehyde for 30 min at room temperature. 3D-EHTs were dehydrated with 30% sucrose overnight before embedding in TissueTek O.C.T. compound (VWR). Cryoblocks were sectioned at a 5 µm thickness. Slides housing tissue sections were blocked and permeabilized with 1% Bovine Serum Albumin (Sigma-Aldrich) and 0.1% Triton-X-100 (Sigma-Aldrich) for 1 h at room temperature. Primary antibodies were diluted in the blocking buffer and incubated overnight at 4 °C (mouse anti-Sarcomeric α-actinin [Abcam ab# ab9465, used at 1:200 dilution], and mouse anti-Titin M-line [Myomedix #M8M10, used at 1:200 dilution]). After three washes with DPBS (5min/each), slides were incubated for 1 h at room temperature with secondary antibodies diluted in blocking buffer (AlexaFluor 488 goat anti-rabbit IgG1 and AlexaFluor 647 goat anti-mouse IgG1, both used at 1:500 dilution). Tissue slides were mounted with coverslips using Vectashield mounting media with DAPI (Vector). Images were taken with a 60x oil objective on a Nikon Ti microscope and formatted with Fiji software.

Supplemental References

Alexa, A., Rahnenfuhrer, J., and Lengauer, T. (2006). Improved scoring of functional groups from gene expression data by decorrelating GO graph structure. Bioinformatics *22*, 1600-1607.

Anders, S., Pyl, P.T., and Huber, W. (2015). HTSeq--a Python framework to work with high-throughput sequencing data. Bioinformatics *31*, 166-169.

Andrews, S. (2010). FastQC: a quality control tool for high throughput sequence data.

Bielawski, K.S., Leonard, A., Bhandari, S., Murry, C.E., and Sniadecki, N.J. (2016). Real-Time Force and Frequency Analysis of Engineered Human Heart Tissue Derived from Induced Pluripotent Stem Cells Using Magnetic Sensing. Tissue Eng Part C Methods *22*, 932-940.

Butler, A., Hoffman, P., Smibert, P., Papalexi, E., and Satija, R. (2018). Integrating single-cell transcriptomic data across different conditions, technologies, and species. Nat Biotechnol *36*, 411-420.

Chen, V.C., Ye, J., Shukla, P., Hua, G., Chen, D., Lin, Z., Liu, J.C., Chai, J., Gold, J., Wu, J.*, et al.* (2015). Development of a scalable suspension culture for cardiac differentiation from human pluripotent stem cells. Stem Cell Res *15*, 365-375.

Dobin, A., Davis, C.A., Schlesinger, F., Drenkow, J., Zaleski, C., Jha, S., Batut, P., Chaisson, M., and Gingeras, T.R. (2013). STAR: ultrafast universal RNA-seq aligner. Bioinformatics *29*, 15-21.

Langmead, B., and Salzberg, S.L. (2012). Fast gapped-read alignment with Bowtie 2. Nat Methods *9*, 357-359.

Liang, X., Potter, J., Kumar, S., Zou, Y., Quintanilla, R., Sridharan, M., Carte, J., Chen, W., Roark, N., Ranganathan, S.*, et al.* (2015). Rapid and highly efficient mammalian cell engineering via Cas9 protein transfection. J Biotechnol *208*, 44-53.

Martin, M. (Cutadapt Removes Adapter Sequences From High-Throughput Sequencing Reads). Cutadapt Removes Adapter Sequences From High-Throughput Sequencing Reads EMBnet.

Ritchie, M.E., Phipson, B., Wu, D., Hu, Y., Law, C.W., Shi, W., and Smyth, G.K. (2015). limma powers differential expression analyses for RNA-sequencing and microarray studies. Nucleic Acids Res *43*, e47.

Robinson, M.D., McCarthy, D.J., and Smyth, G.K. (2010). edgeR: a Bioconductor package for differential expression analysis of digital gene expression data. Bioinformatics *26*, 139-140.

Robinson, M.D., Oshlack, A. (2010). A scaling normalization method for differential expression analysis of RNA-seq data. Genome Biol *11*.

Stuart, T., Butler, A., Hoffman, P., Hafemeister, C., Papalexi, E., Mauck, W.M., 3rd, Hao, Y., Stoeckius, M., Smibert, P., and Satija, R. (2019). Comprehensive Integration of Single-Cell Data. Cell *177*, 1888-1902 e1821.
