# Supplementary material for: SARS-CoV-2 Infects Human Pluripotent Stem Cell-Derived Cardiomyocytes, Impairing Electrical and Mechanical Function
Source: Stem Cell Reports. 2021 Feb 13;16(3):478–92. doi: 10.1016/j.stemcr.2021.02.008 (PMC7881699; doi:10.1016/j.stemcr.2021.02.008)
Supplement: Document S2. Article plus Supplemental Information [file mmc4.pdf]

Stem Cell Reports
Article
OPEN ACCESS

# SARS-CoV-2 Infects Human Pluripotent Stem Cell-Derived Cardiomyocytes, Impairing Electrical and Mechanical Function

Silvia Marchiano,[1,2,3] Tien-Ying Hsiang,[4] Akshita Khanna,[1,2,3] Ty Higashi,[1,2,3,5] Leanne S. Whitmore,[4] Johannes Bargehr,[6,7] Hongorzul Davaapil,[6,7] Jean Chang,[4] Elise Smith,[4] Lay Ping Ong,[6,7] Maria Colzani,[6,7] Hans Reinecke,[1,2,3] Xiulan Yang,[1,2,3] Lil Pabon,[1,2,3,8] Sanjay Sinha,[6,7] Behzad Najafian,[1] Nathan J. Sniadecki,[1,2,3,5,9] Alessandro Bertero,[1,2,3] Michael Gale, Jr.,[4,*] and Charles E. Murry[1,2,3,8,9,10,*]
[1]Department of Laboratory Medicine and Pathology, University of Washington, 1959 NE Pacific Street, Seattle, WA 98195, USA
[2]Center for Cardiovascular Biology, University of Washington, 850 Republican Street, Brotman Building, Seattle, WA 98109, USA
[3]Institute for Stem Cell and Regenerative Medicine, University of Washington, 850 Republican Street, Seattle, WA 98109, USA
[4]Center for Innate Immunity and Immune Disease, Department of Immunology, University of Washington School of Medicine, Seattle, WA 98109, USA
[5]Department of Mechanical Engineering, University of Washington, 3720 15th Avenue NE, Seattle, WA 98105, USA
[6]Wellcome – MRC Cambridge Stem Cell Institute, Jeffrey Cheah Biomedical Centre, Cambridge Biomedical Campus, University of Cambridge, Puddicombe Way, CB2 0AW Cambridge, UK
[7]Division of Cardiovascular Medicine, University of Cambridge, Addenbrooke's Hospital, ACCI Level 6, Box 110, Hills Road, Cambridge CB2 0QQ, UK
[8]Sana Biotechnology, 188 E Blaine Street, Seattle, WA 98102, USA
[9]Department of Bioengineering, University of Washington, 3720 15th Avenue NE, Seattle, WA 98105, USA
[10]Department of Medicine/Cardiology, University of Washington, 1959 NE Pacific Street, Seattle, WA 98195, USA
*Correspondence: mgale@uw.edu (M.G.), murry@uw.edu (C.E.M.)
https://doi.org/10.1016/j.stemcr.2021.02.008

## SUMMARY

COVID-19 patients often develop severe cardiovascular complications, but it remains unclear if these are caused directly by viral infection or are secondary to a systemic response. Here, we examine the cardiac tropism of SARS-CoV-2 in human pluripotent stem cell-derived cardiomyocytes (hPSC-CMs) and smooth muscle cells (hPSC-SMCs). We find that that SARS-CoV-2 selectively infects hPSC-CMs through the viral receptor ACE2, whereas in hPSC-SMCs there is minimal viral entry or replication. After entry into cardiomyocytes, SARS-CoV-2 is assembled in lysosome-like vesicles and egresses via bulk exocytosis. The viral transcripts become a large fraction of cellular mRNA while host gene expression shifts from oxidative to glycolytic metabolism and upregulates chromatin modification and RNA splicing pathways. Most importantly, viral infection of hPSC-CMs progressively impairs both their electrophysiological and contractile function, and causes widespread cell death. These data support the hypothesis that COVID-19-related cardiac symptoms can result from a direct cardiotoxic effect of SARS-CoV-2.

## INTRODUCTION

With over 100 million people affected worldwide, the outbreak of severe acute respiratory syndrome coronavirus 2 (SARS-CoV-2) has already left its permanent mark on human history (Hopkins, 2020; Zhu et al., 2020). SARS-CoV-2 belongs to the family of *Coronaviridae*, a large group of single-stranded enveloped RNA viruses reported for the first time in humans in the 1960s (Andersen et al., 2020; Corman et al., 2018; Cui et al., 2019). Besides being long recognized as one of the common cold viruses, coronaviruses took center stage in infectious disease medicine following the outbreaks of SARS-CoV in 2003 and of Middle East respiratory syndrome coronavirus (MERS-CoV) a decade later. Coronaviruses became thus recognized as highly pathogenic for humans, with a symptomatology that focuses on the respiratory system while often also involving extra-respiratory organs (Alhogbani, 2016; Nishiga et al., 2020; Oudit et al., 2009; Zhou et al., 2020). Indeed, even though the lungs represent the main target, cardiovascular complications (including worsening of pre-existing conditions and onset of new disorders) were not only reported for SARS-CoV and MERS-CoV, but are also significantly contributing to the mortality of COVID-19 patients during the ongoing pandemic (Alhogbani, 2016; Oudit et al., 2009; Shi et al., 2020; Zhou et al., 2020).

The most common cardiovascular complications observed after SARS-CoV-2 infection are myocardial injury (including cases with and without classic coronary occlusion), arrhythmias, and heart failure (Baggiano et al., 2020; Nishiga et al., 2020; Ojha et al., 2020; Ruan et al., 2020; Shi et al., 2020; Wang et al., 2020; Xu et al., 2020; Zhou et al., 2020). In particular, myocardial injury, characterized by elevated serum levels of cardiac troponin I and/or electrocardiogram abnormalities, has been independently associated with increased mortality in COVID-19 patients (Guo et al., 2020). Moreover, as reported also for SARS-CoV (Madjid et al., 2007), SARS-CoV-2 can trigger acute coronary syndrome even in the absence of systemic inflammation (Nishiga et al., 2020; Wang et al., 2020). Retrospective studies show that hospitalized COVID-19 patients develop cardiac arrhythmias, including ventricular tachycardia and atrial fibrillation (Bhatla et al., 2020; Malaty et al., 2020; Wang et al., 2020; Zylla et al., 2021).

 © 2021 The Authors.
This is an open access article under the CC BY license (http://creativecommons.org/licenses/by/4.0/).

Progressive left ventricular dysfunction and overall symptoms that resemble heart failure have also been observed in a significant number of patients (Dong et al., 2020; Huang et al., 2020a; Wang et al., 2020; Zhou et al., 2020). At the beginning of the outbreak, this symptomatology was reported mostly in critically ill COVID-19 patients (Zhou et al., 2020). A sizable number of more recent studies has reported that cardiac symptoms are observed also in mild and even asymptomatic cases of COVID-19 (Arentz et al., 2020; Huang et al., 2020b; Inciardi et al., 2020; Puntmann et al., 2020; Rajpal et al., 2021).

The mechanisms behind cardiac disease reported for COVID-19 are still unclear (Nishiga et al., 2020; Zhou et al., 2020). Upon lung infection, the uncontrolled release of inflammatory cytokines, termed "cytokine storm," could induce multi-organ damage, ultimately leading to organ failure and worsening of pre-existing cardiovascular disorders (Driggin et al., 2020; Inciardi et al., 2020; Tay et al., 2020). Moreover, COVID-19 is associated with coagulopathies, which also can induce ischemic heart damage (Driggin et al., 2020; Nishiga et al., 2020; Ranucci et al., 2020). Finally, SARS-CoV-2 could directly mediate heart injury by entering cardiomyocytes or other cardiac stromal and/or vascular cells via binding of the viral spike glycoprotein to its extracellular receptor, angiotensin I converting enzyme 2 (ACE2) (Baggiano et al., 2020; Hoffmann et al., 2020). This protein is expressed in different tissues of the human body, including cardiomyocytes and cardiac pericytes, and its primary function is to counterbalance the renin-angiotensin-aldosterone system (Chen et al., 2020a; Hikmet et al., 2020; Li et al., 2020; Verdecchia et al., 2020).

Several studies detected SARS-CoV-2 genome in the heart and/or reported signs of viral myocarditis in COVID-19-infected individuals, including asymptomatic cases (Bradley et al., 2020; Dolhnikoff et al., 2020; Lindner et al., 2020; Rajpal et al., 2021). Moreover, *in vivo* and *in vitro* studies utilizing both human adult cardiomyocytes and human pluripotent stem cell-derived cardiomyocytes (hPSC-CMs) have shown that SARS-CoV-2 can infect cardiomyocytes, indicating that SARS-CoV-2 could exhibit cardiac tropism (Bojkova et al., 2020; Chen et al., 2020b; Sharma et al., 2020; Yang et al., 2020). However, whether SARS-CoV-2 infection of human cardiomyocytes leads to a direct impairment of cardiac function is still unresolved. Furthermore, whether other cardiac cell types are also susceptible to SARS-CoV-2 remains unclear.

In this study we examine the mechanisms behind COVID-19-related cardiac symptoms using hPSC-CMs and hPSC-derived smooth muscle cells (hPSC-SMCs), established models for cardiovascular disease research (Bertero et al., 2019b; Cheung et al., 2012; Serrano et al., 2019; Yang et al., 2018). SARS-CoV-2 specifically infects and propagates within hPSC-CMs, a process that appears to exquisitely rely on ACE2 and to both involve direct membrane fusion and entry through the endo-lysosomal pathway. Pathways involved in RNA splicing and chromatin accessibility are significantly upregulated after infection, whereas pathways involved in oxidative metabolism and mitochondrial function are downregulated. SARS-CoV-2 infection results in disruption of the contractile cytoskeleton, electrical and mechanical dysfunction, and eventual cell death. These findings provide evidence for a direct viral cytopathic pathway involving cardiac myocytes in the context of COVID-19-related cardiac disease.

## RESULTS

### hPSC-CMs Express SARS-CoV-2 Receptors and Entry Cofactors

Susceptibility to SARS-CoV-2 infection is thought to depend on expression of both the viral receptor ACE2 and various host proteases (Hoffmann et al., 2020; Millet and Whittaker, 2015; Shang et al., 2020; Zumla et al., 2016). We found that *ACE2* is transcriptionally activated during cardiac differentiation of both RUES2 embryonic stem cell-derived cardiomyocytes (hESC-CMs; Figure 1A) and WTC11c-induced pluripotent stem cell-derived cardiomyocytes (hiPSC-CMs; Figure S1A). Single-cell RNA sequencing (RNA-seq) analysis detected *ACE2* mRNA in ~9% of hESC-CMs, indicating low and/or transitory expression (Figure 1B). A larger fraction of cells expressed moderate to high levels of endosomal cysteine proteases *CTSB* (cathepsin B; ~71.0%) and *CTSL* (cathepsin L; ~46.0%). Detection of these factors is relevant because they can cleave the spike glycoprotein leading to endomembrane fusion-mediated release of the SARS-CoV-2 genome inside the cytoplasm (Kang et al., 2020; Millet and Whittaker, 2015; Ou et al., 2020; Yang and Shen, 2020). Importantly, these viral processing factors were often co-expressed with *ACE2* (Figure S1B). Although viral entry can also be mediated by *TMPRSS2* (Hoffmann et al., 2020; Shulla et al., 2011), this transmembrane serine protease was not detectable in hESC-CMs (Figure S1C), as also reported for the adult human heart (Litvinukova et al., 2020). Interestingly, the lipid phosphatase, *PIKFYVE*, another endosomal viral processing factor, and *FURIN*, a membrane-bound serine protease, were also broadly expressed in hESC-CMs (Figure S1C), overall suggesting that the mechanism of SARS-CoV-2 entry in cardiomyocytes might be different from the TMPRSS2-dependent one reported for lung epithelial cells (Hoffmann et al., 2020; Schneider et al., 2020; Shang et al., 2020; Xia et al., 2020b).

Despite the relatively low levels of mRNA, ACE2 protein was clearly detectable by western blot in hPSC-CMs derived from multiple lines (RUES2 female hESCs, H7 female

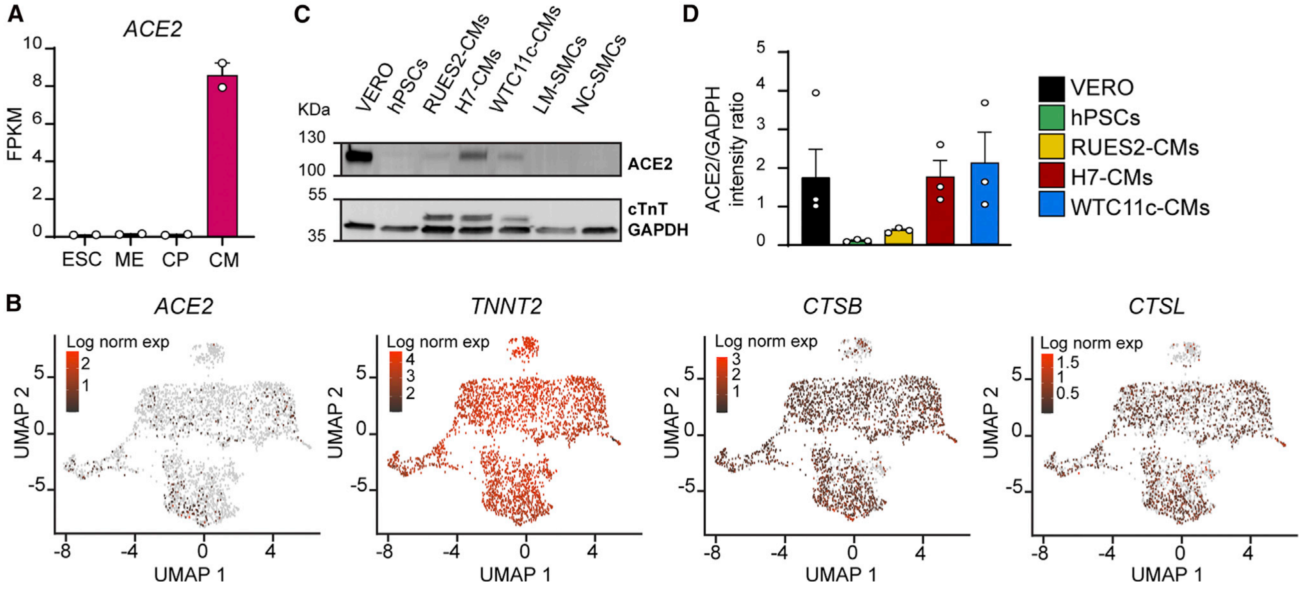


**Figure 1. hPSC-CMs Express SARS-CoV-2 Receptors and Processing Factors**

(A) RNA-seq during RUES2 hESC-CM differentiation. *ACE2* is quantified as fragments per kilobase of transcript per million mapped reads (FPKM). Mean ± SEM of two independent experiments. ESC, embryonic stem cells (day 0); ME, mesoderm (day 2); CP, cardiac progenitors (day 5); CM, cardiomyocytes (day 14).

(B) Single-cell RNA-seq gene expression heatmaps from RUES2 hESC-CMs after dimensionality reduction through Uniform Manifold Approximation and Projection (UMAP). *TNNT2* provides a pan-cardiomyocyte marker.

(C) Western blot for ACE2 in hPSC-CMs from multiple lines and different types of hPSC-SMCs. LM, lateral plate mesoderm-derived; NC, neural crest-derived; hPSCs, negative control; VERO cells, positive control.

(D) Quantification of ACE2 level normalized by GADPH. Mean ± SEM of three independent experiments.

hESCs, and WTC11c male hiPSCs), reaching levels comparable with those of VERO cells, a primate kidney epithelial line with established SARS-CoV-2 tropism (Figures 1C and 1D). Emphasizing the specific tropism of SARS-CoV-2 for hPSC-CMs, ACE2 was expressed at very low levels in hESC-SMCs of varying embryonic origin (lateral mesoderm- or neural crest-derived, all differentiated from H9 female hESCs; Figures 1C and S1D). Collectively, hPSC-CMs express proteins that may render them susceptible to SARS-CoV-2 infection (Bojkova et al., 2020; Sharma et al., 2020; Yang et al., 2020).

## SARS-CoV-2 Can Infect and Replicate in hPSC-CMs Using ACE2

Since H7- and WTC11c-derived hPSC-CMs showed the highest levels of ACE2 (Figures 1C and 1D), we tested their functional susceptibility to SARS-CoV-2. For this, we incubated highly pure hPSC-CMs (over 80% positive for cardiac troponin T [cTnT+]; Figure S2A) with SARS-CoV-2/Wa-1 strain. We used a multiplicity of infection (MOI) (i.e., the number of infectious viral particles per cell) of either 0.1 (requiring propagation of the virus within the cells and secondary infection of others) or 5 (aiming to infect all susceptible cells at the same time). We observed marked and disseminated viral cytopathic effects in both H7 hESC-CMs and WTC11c hiPSC-CMs. These effects were accelerated at 5 MOI, as expected (Figures 2A and S2B). Most notably, the highest MOI led to cessation of beating and signs of cell death as early as at 48 h post infection (hpi) in both cell lines, with more pronounced effects for H7 cardiomyocytes. Immunofluorescence staining of SARS-CoV-2 nucleocapsid protein revealed substantial presence of viral factors in the cytoplasm of both H7 hESC-CMs and WTC11c hiPSC-CMs (Figures 2B and S2C).

To investigate whether hPSC-CMs are permissive to SARS-CoV-2 replication, we quantified extracellular viral particles and intracellular viral RNA (by plaque assay and quantitative reverse transcription PCR [qRT-PCR], respectively). The one-step growth curve after 5 MOI infection indicated that viral replication occurred steadily from 24 to 72 hpi, followed by a precipitous decline as the cells died (Figure 2C). The multi-step growth curve (0.1 MOI infection) confirmed that SARS-CoV-2 replicated inside hPSC-CMs (Figure 2D), with a marked increase in viral particles and RNA at 48 and 72 hpi (at which point the experiment was stopped). In agreement with morphological observations, H7-derived cardiomyocytes were more permissive to SARS-CoV-2 replication than

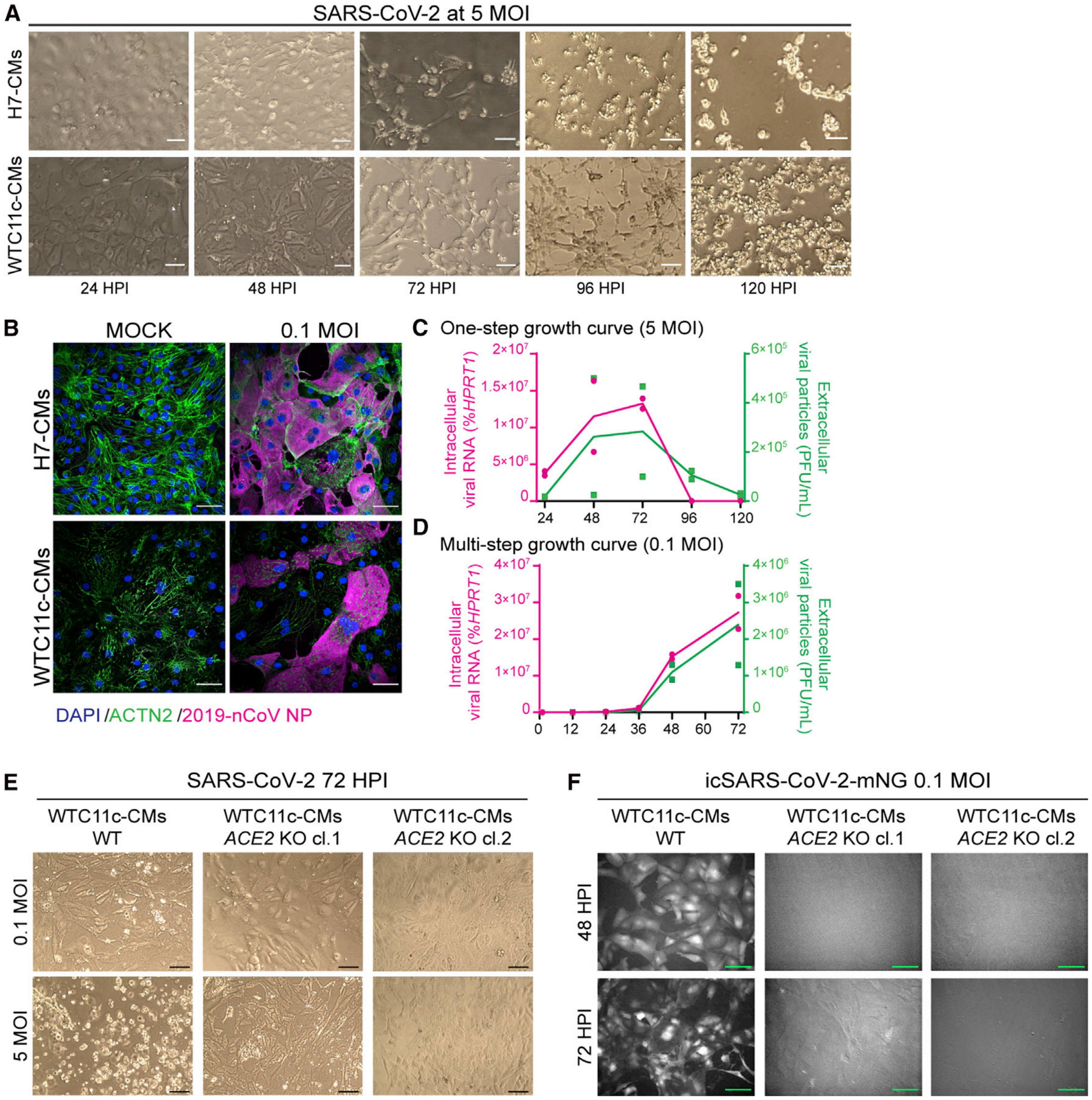


**Figure 2. hPSC-CMs Are Permissive to SARS-CoV-2 Infection and Replication**

(A) Cytopathic effects of SARS-CoV-2 at 5 MOI in H7 hESC-CMs and WTC11c hiPSC-CMs during a time course of 120 h. Scale bars, 100 μm.

(B) Immunofluorescent staining of H7 hESC-CMs and WTC11c hiPSC-CMs at 48 hpi with SARS-CoV-2 dose of 0.1 MOI. Scale bars, 50 μm. Individual channels are shown in Figure S2C.

(C) One-step viral growth curve in H7 hESC-CMs infected with SARS-CoV-2 at 5 MOI over a time course of 120 h.

(D) Multi-step viral growth curve in H7 hESC-CMs infected with SARS-CoV-2 at 0.1 MOI over a time course of 72 h. For both (C) and (D), lines connect the means of two independent experiments. Viral RNA indicating intracellular viral replication is plotted on the left y axis as percent of *HPRT1*. Viral particles secreted in the supernatant are plotted on the right y axis as plaque-forming units (PFU) per mL.

(E) Cytopathic effects of 0.1 MOI and 5 MOI of SARS-CoV-2 at 72 hpi in wild-type (WT) and *ACE2* knockout (KO) WTC11c-CMs. Two *ACE2* KO hiPSC clones (cl.1 and cl. 2) were analyzed (Figure S3). Scale bars, 100 μm.

(F) Fluorescence microscopy assessment of viral entry of icSARS-CoV2-mNG at 0.1 MOI in WTC11c-CMs WT and *ACE2* KO clones at 48 and 72 hpi. mNG fluorescence is shown in grayscale. ACE2 KO clones showed background autofluorescence only. Scale bars, 100 μm.

WTC11c-derived ones (compare Figures 2C, 2D, S2D, and S2E), perhaps as a reflection of genetic or epigenetic differences that deserve further study.

Distinct from hPSC-CMs, hESC-SMCs exposed to SARS-CoV-2 did not show any cytopathic effects even at the highest MOI at 72 hpi (Figure S2F). Accordingly, extracellular

viral particles and intracellular viral RNA in hPSC-SMCs were more than two orders of magnitude lower than the ones observed for hPSC-CMs (Figure S2G). These findings highlight the specific tropism of SARS-CoV-2 for hPSC-CMs, and exclude that cytopathic effects observed in cardiomyocytes may be attributable to toxic contaminants in the viral preparation.

To investigate the role of ACE2 during cardiomyocyte infection by SARS-CoV-2, we generated *ACE2* knockout (KO) WTC11c hiPSC-CMs (Figures 3A–3D). Loss of ACE2 prevented cell death following SARS-CoV-2 exposure even at 5 MOI (Figure 2E). To confirm the absence of viral entry in *ACE2* KO cells, we used SARS-CoV-2 genetically engineered to express the mNeonGreen protein (mNG) (Xie et al., 2020). Similarly to wild-type SARS-CoV-2, the SARS-CoV-2-mNG reporter became detectable at 48 hpi at 0.1 MOI only in wild-type cardiomyocytes, with increased fluorescence intensity at 72 hpi (Figure 2F). Overall, these findings indicate that SARS-CoV-2 infection in cardiomyocytes is prominently mediated by the expression of ACE2.

### SARS-CoV-2 Viral Entry, Replication, and Egress Engage Lysosome-like Structures

To examine in finer detail the viral propagation mechanisms in hPSC-CMs we performed extensive electron microscopy analyses. We readily identified numerous viral particles entering and replicating inside the cytoplasm of WTC11c hiPSC-CMs (Figures 3A and 3B), clearly visible as 80–90-nm-wide spherical structures, confirming that hPSC-CMs can be infected directly by SARS-CoV-2. The presence of viruses was remarkably greater at 72 hpi. Interestingly, we observed both endocytosis of intact virions (Figure 3A) and direct fusion of viral envelope with cell membrane (Figure 3B), suggesting dual mechanisms of entry in cardiomyocytes. Double-membrane vesicles, organelles associated with viral replication and assembly, were commonly seen in close association with viral particles (Figure 3C) (Snijder et al., 2020). We also observed dilated membrane-bound tubular structures, likely representing the endoplasmic reticulum Golgi intermediate compartment, in close proximity to membrane-enclosed viral particles (Figure 3D). In addition, we observed a variety of vesicles containing viruses (Figures 3D–3F). Most notable were large vesicles with electron dense content packed with mature virus particles (Figure 3E). In some of these vesicles, we also observed lipid droplets and multilamellar bodies, consistent with lysosomes, whereas others were smooth-walled vesicles (Figures 3E and 3F) (Ghosh et al., 2020; Snijder et al., 2020). Finally, exocytosis of virions was readily identifiable on the cell surface (Figures 3G–3I). In summary, these electron microscopic studies demonstrated viral entry via both direct fusion and endocytosis, replication in subcellular membrane structures, and "hijacking" of lysosomal vesicles for the bulk release of mature virions by exocytosis.

### SARS-CoV-2 Reprograms Chromatin-Modifying, RNA Processing, and Energy Metabolism Pathways

To clarify the genome-wide transcriptional alterations induced by SARS-CoV-2 infection in hPSC-CMs, we performed RNA-seq analyses in both H7 hESC-CMs and WTC11c hiPSC-CMs subjected to infection at 0.1 or 5 MOI and sampled every 24 h for 3 days. A high fraction of next-generation sequencing reads mapped to the SARS-CoV-2 genome at high MOI and/or late time points, particularly for H7 hESC-CMs (up to ~18% of all reads), in agreement with their stronger susceptibility to SARS-CoV-2 infection (Figure 4A). Dimensionality reduction of the data with principal-component analysis (PCA) showed that gene expression variability correlated most strongly with the cell type of origin (PC1) and subsequently with the degree and progression of SARS-CoV-2 infection (PC2) (Figure 4B). Accordingly, gene expression profiles change in a time- and dose-dependent manner for both H7-CMs and WTC11c-CMs, with major differences observed at the latest time point and with the highest MOI (Figures 4C and 4D). Interestingly, pathways involved in RNA processing and chromatin accessibility (i.e., histone modification) were upregulated together with the response to viral infection, suggesting that SARS-CoV-2 may induce broad epigenetic reprogramming of the host to promote its own replication (Figures 4E and S4A; Tables S1 and S3) (Banerjee et al., 2020). In addition, we observed that genes involved in mitochondrial function and energy production were downregulated (Figures 4F and S4B; Tables S2 and S4), indicating that SARS-CoV-2 might promote a shift toward a glycolytic metabolism by suppressing mitochondrial oxidative phosphorylation, which could also favor its replication (Ajaz et al., 2021; Icard et al., 2021).

Upon viral infection, pathogen-associated molecular patterns (PAMPs) initiate the early immune response via host pattern recognition receptors. After virus uncoating, RIG-I-like receptors bind to the uncapped and double-stranded viral RNA in the cytosol and trigger innate immune activation, leading to the production of type I and type III interferons and the interferon-induced antiviral response (Loo and Gale, 2011). Upregulated pathways after SARS-CoV-2 infection in hPSC-CMs included those involved in viral defense (Figure 4E). To more finely clarify the underlying kinetics, we analyzed the interferon response from 2 to 72 hpi by qRT-PCR. We found that interferon transcripts (*INFB1* and *IFNL1)* were markedly upregulated at 48 and 72 hpi in both cell lines, with a stronger effect in the more sensitive H7 cardiomyocytes (Figures 4G and S4C). The interferon-stimulated genes *IFIT1* and *IFITM1* were also upregulated at the latest time point. These results

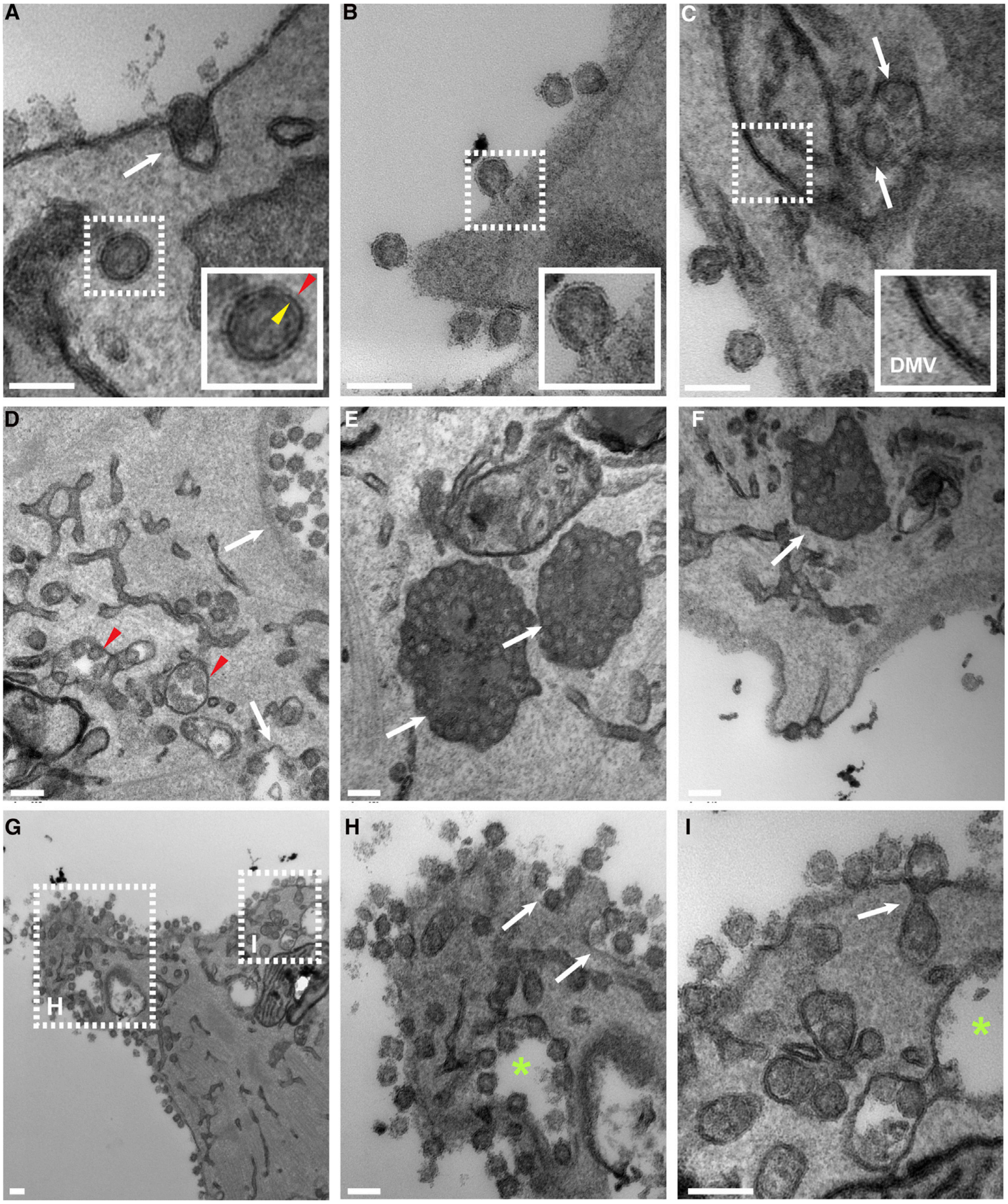


**Figure 3. Electron Microscopy Analysis of Infected hPSC-CMs**

(A) SARS-CoV-2 enters WTC11c-CMs by endocytosis (white arrow). The dashed box (magnified in the inset) shows a virus in an endosome. Note that two lipid layers are identifiable. The outer layer (red arrowhead) belongs to the endosome and the inner layer (yellow arrowhead) to the virus.

(B) A virion envelope fuses directly with the cell membrane upon entry (dashed box and magnified view in the inset).

(C) A double-membrane vesicle (DMV) (dashed box and magnified in the inset) adjacent to two completely formed virus particles inside a vesicle (arrows).

(D–F) Numerous intracellular virus particles packed into different types of vesicles. White arrows in (D) show smooth-walled vesicles, and red arrowheads show vesicles with branched connections, most consistent with the endoplasmic reticulum Golgi intermediate compartment. White arrows in (E and F) show vesicles with viral particles and electron dense content, likely representing lysosomes.

*(legend continued on next page)*

indicate that SARS-CoV-2 induces innate immune activation and interferon response in hPSC-CMs, similar to other cell types (Lei et al., 2020).

### Electrophysiological Characteristics of hPSC-CMs Infected with SARS-CoV-2

We next investigated whether SARS-CoV-2 infection impairs the function of hPSC-CMs. First, we evaluated electrophysiological properties of infected H7 hESC-CMs and WTC11c hiPSC-CMs using multi-electrode arrays (MEA) over a time course of 72 hpi at MOI of 0.1 and 5. Distinct from our earlier experiments on sparser hPSC-CM cultures in standard tissue culture dishes, in this context we did not observe profound cytopathic effects in any of the conditions (Figures 5A and S5A). This outcome may reflect a decreased efficiency of viral propagation as hPSC-CMs were plated at high density to ensure robust assessment of electrophysiological properties. Nevertheless, viral RNA and viral particles could still be detected at 0.1 MOI, with the highest levels at 5 MOI (Figures 5B and S5B), showing that the infection occurred also in these highly dense cultures. Representative propagation maps are shown in Figures 5C and S5C, while representative field potential recordings showcasing changes in spike amplitude and frequency are included in Figures 5D and S5D. Remarkably, SARS-CoV-2 infection rapidly resulted in reduced beating rate, lower depolarization spike amplitude, and decreased electrical conduction velocity (Figures 5E, S5E, and S5F). In H7 hESC-CMs we also observed a time-dependent increase in the field potential duration (FPD) both in spontaneously beating and electrically paced cultures (Figure 5F; similar measurements could not be reliably obtained from WTC11c hiPSC-CMs due to the limited amplitude of the repolarization wave after SARS-CoV-2 infection). Overall, abnormalities in the generation and propagation of electrical signals were significant even in the absence of extensive cell death, suggesting that SARS-CoV-2 infection in cardiomyocytes could directly create a substrate for arrhythmias (Bhatla et al., 2020; Zylla et al., 2021).

### SARS-CoV-2 Infection Progressively Impairs Force Generation in Engineered Heart Tissues

We then evaluated the contractile properties of hPSC-CMs using three-dimensional engineered heart tissues (3D-EHTs), following their contractile behavior through magnetic field sensing (Bielawski et al., 2016) (Figures 6A and 6B). For these experiments we focused on WTC11c hiPSCs since 3D-EHTs from H7 hESC-CMs proved to beat spontaneously at too high a frequency (>2 Hz) to enable accurate measurements of contractile behavior (i.e., the tissue had a tetanic-like contraction with minimal relaxation between beats at this frequency). We infected 3D-EHTs from WTC11c hiPSC-CMs with 10 MOI (to facilitate infection within the non-vascularized, cell-dense tissue), and analyzed their contraction for a week. This 3D model experienced viral replication comparable with that of 2D cultures, highlighting once again the cardiac tropism of SARS-CoV-2 (Figure 6C). The maximal twitch force in infected tissues decreased as early as 72 hpi (Figure S6A), and the contractions continued to subside to less than 25% of the force measured at the baseline at 144 hpi (Figures 6D and 6E; Videos S1 and S2). Cardiomyocyte density progressively decreased while cells also became more rounded (i.e., dedifferentiated) and less aligned with the longitudinal axis of the 3D-EHTs (Figures 6F and S6B). This could collectively contribute to the loss of force production. Infected 3D-EHTs also showed decreased expression of the sarcomeric genes *MYL2* and *MYH6*, which may be correlated to the loss of sarcomere organization (Figure 6G). Overall, the significant impairment in the contractile properties of 3D-EHTs demonstrates that the mechanical function of cardiomyocytes is impacted by SARS-CoV-2 infection *in vitro*, and suggest that similar mechanisms could contribute to whole-organ cardiac dysfunction also in patients (Dong et al., 2020).

## DISCUSSION

A rapidly increasing number of reports acknowledge cardiovascular involvement as a prevalent complication observed in COVID-19 patients, but discriminating between direct versus indirect effects is still an open challenge (Nishiga et al., 2020; Shi et al., 2020; Tay et al., 2020). In this study, we show that SARS-CoV-2 has the ability to directly infect cardiomyocytes, to impair both their electrophysiological and contractile properties, and to eventually induce cell death.

In agreement with earlier reports, we find that cardiomyocytes (but not smooth muscle cells) express ACE2, making them susceptible to SARS-CoV-2 infection. Our experiments in ACE2 KO hPSC-CMs formally demonstrate the key role of this factor for SARS-CoV-2 entry in this cell type. Interestingly, mRNA levels of ACE2 are heterogeneous within hPSC-CMs from the same culture, and the resulting protein is differentially abundant in hPSC-CMs from different genetic backgrounds. The cytopathic effects of SARS-CoV-2 infection also strongly vary between

(G–I) Virions egressing the cell. Dashed boxes in (G) are magnified in (H and I). The asterisks in (H and I) show smooth-walled vesicles. The white arrows show vesicles opening into the extracellular space (exocytosis), releasing their virions. Scale bars, 100 μm.

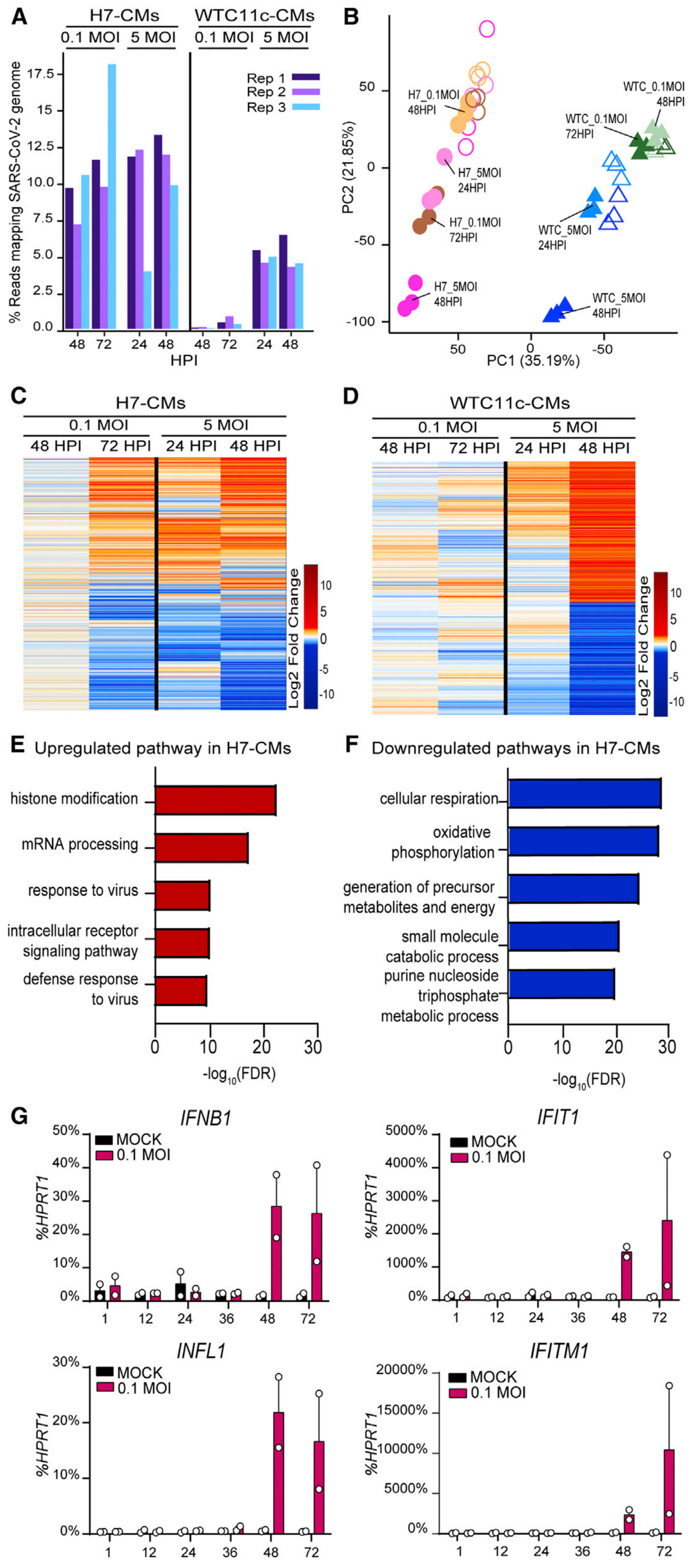


**Figure 4. Gene Expression Analysis in hPSC-CMs Infected with SARS-CoV-2**

(A) Percentage of RNA-seq reads mapping to the SARS-CoV-2 genome in hPSC-CMs infected with 0.1 or 5 MOI SARS-CoV-2 at different time points (three technical replicates per condition).

(B) Principal-component analysis (PCA) plot describing the gene expression variation between samples. Circles and triangles identify H7-CMs and WTC11c-CMs, respectively. The outline of empty symbols (uninfected samples) are color-matched to filled symbols (infected samples) by time point and MOI.

(C and D) Heatmaps showing significant differentially expressed genes for infected samples versus mock in H7-CMs (C) and WTC11c-CMs (D).

(E) Gene ontology (GO) analysis of upregulated pathways in H7-CMs infected with 5 MOI at 48 hpi.

(F) GO analysis of downregulated pathways in H7-CMs infected with 5 MOI at 48 hpi.

(**G**) qRT-PCR of interferon response genes in H7 hESC-CMs infected with SARS-CoV-2 at 0.1 MOI. Mean ± SEM of two independent experiments.

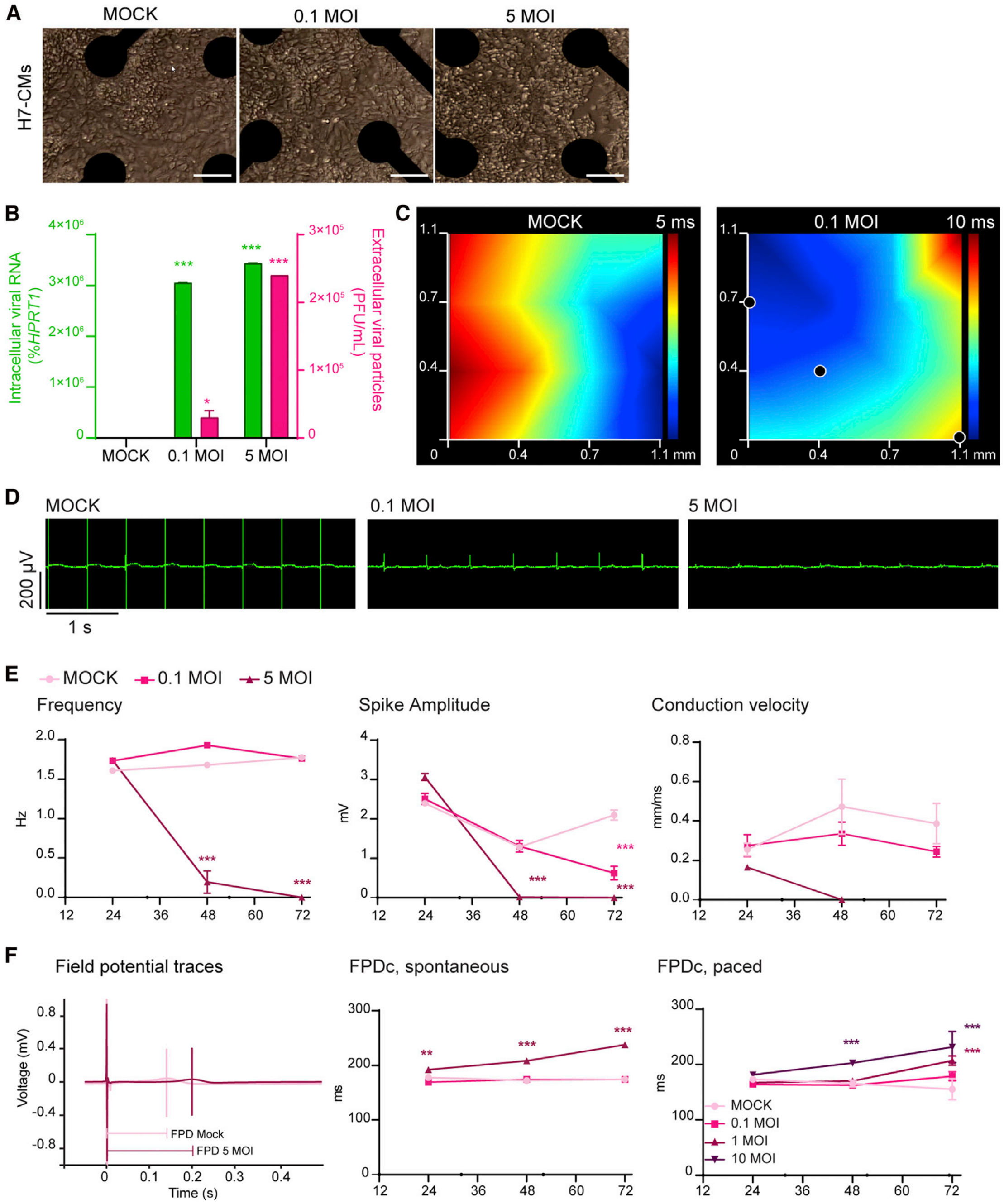


**Figure 5. Electrophysiological Alterations in hPSC-CMs Infected with SARS-CoV-2**

(A and B) (A) Representative images of SARS-CoV-2-infected H7 hESC-CMs on MEA wells at 72 hpi. Scale bars, 50 μm. (B) Intracellular and extracellular viral particles in H7 hESC-CMs seeded and infected in the MEA plate. Intracellular viral particles are plotted on the right axis as viral RNA (percent of *HPRT1*); extracellular viral particles are plotted on the left axis as PFU/mL. Data are shown as mean ± SEM of eight wells. Differences versus mock control by two-way ANOVA with Sidak correction for multiple comparisons (* $p < 0.05$, *** $p < 0.001$).

(C) Representative propagation maps at 72 hpi. The axes illustrate the position of 16 total electrodes, with black dots indicating inactive ones. Electrical propagation starts in the blue area and moves toward the red area, according to the color-coded time interval.

(D) Representative recordings of spontaneous electrical activity of SARS-CoV-2-infected H7 hESC-CMs at 72 hpi.

(E and F) (E) Representative quantifications of electrophysiological properties from MEA analyses in SARS-CoV-2-infected H7 hESC-CMs. Mean ± SEM of eight wells. Statistical analyses of intra-experimental variability as for (B). (F) Representative field potential traces in SARS-CoV-2-infected H7 hESC-CMs at 72 hpi, and quantification of FPD corrected by beat rate in spontaneous and paced experiments. Mean

(legend continued on next page)

cardiomyocytes derived from different hPSC lines. ACE2 expression and SARS-CoV-2 susceptibility may be similarly heterogeneous *in vivo*, both across different regions of the heart and within different subjects (Litvinukova et al., 2020). This might partially explain the discrepancy between the strong prevalence of heart damage in COVID-19 patients and the limited evidence for viral particles in the heart found by autopsy examinations. We suggest that future analyses should aim to sample various regions of the heart and focus on those patients that had shown the strongest cardiac symptoms.

A puzzling observation is that the cytopathic effects of SARS-CoV-2 infection expands to virtually the entire monolayer of hPSC-CMs, even though single-cell RNA-seq indicates that many cardiomyocytes do not express detectable levels of *ACE2*. One possible explanation is that ACE2 transcription is episodic and/or still meaningful low levels that are below the sensitivity of single-cell RNA-seq (either way resulting in sufficient protein levels to allow SARS-CoV-2 entry). Alternatively, cytotoxic stimuli triggered by SARS-CoV-2 might spread to the adjacent cells via gap junctions or through the supernatant as toxic cytokines and/or other danger signals. Finally, the fusogenic properties of the SARS-CoV-2 spike protein may mediate membrane fusion not only between viral and host membranes but also within cardiomyocytes, leading to intercellular viral spreading (Schneider et al., 2020). Spike protein fusogenicity is secondary to its proteolytic cleavage by host proteases, such as furin (Millet and Whittaker, 2015), which is expressed in hPSC-CMs. Noticeably, we observed direct fusion between the virus and hPSC-CMs, a process that may be mediated by proteolytically primed spike proteins, as in the case of MERS-CoV (Millet and Whittaker, 2014; Xia et al., 2020a, 2020b). Future studies are needed to clarify whether any of these non-mutually exclusive mechanisms are involved in the strong susceptibility of hPSC-CMs to SARS-CoV-2 infection, and whether similar events are recapitulated *in vivo*.

We found that hPSC-CMs are also extremely permissive to viral replication. Among other types of ultrastructural features, we detected the presence of double-membrane vesicles. These are used by viruses both to concentrate their building materials for efficient replication, and to evade the immune surveillance by “hiding” viral factors that can trigger the PAMP pathway (Wolff et al., 2020). Accordingly, we observed activation of interferon-responsive genes only at late time points of SARS-CoV-2 infection. The interferon response, which is part of the innate immune response activation (Loo and Gale, 2011), usually occurs within hours from viral infection. The fact that cardiomyocytes infected with SARS-CoV-2 show a delayed response may facilitate viral replication to high levels (Lei et al., 2020). Furthermore, RNA-seq showed that SARS-CoV-2 infection affects pathways involved in RNA regulation. SARS-CoV-2 can impair RNA splicing to evade the intracellular innate immune response (Banerjee et al., 2020), which may be the case also in hPSC-CMs. Moreover, oxidative phosphorylation and mitochondrial function are severely downregulated in the infected cells. SARS-CoV-2 may shift cellular metabolism to promote glycolytic metabolic activity in support of viral replication (Icard et al., 2021), providing yet another way to boost its replication also in hPSC-CMs.

The presence of highly replicating virus severely affects both the morphology and the function of hPSC-CMs. Infected cardiomyocytes lose cytoskeletal organization, become packed with different types of vesicles, and show broad alterations of gene expression. Using an MEA system, which has been validated to detect potential arrhythmogenic properties of novel drugs (Blinova et al., 2018), we identified several electrophysiological abnormalities induced by SARS-CoV-2 infection. Prolongation of FPD is particularly noticeable. This measurement reflects the interval between membrane depolarization and repolarization, and as such represents an *in vitro* surrogate of the QT interval measured by an electrocardiogram. It is well known that prolongation of the QT interval is pro-arrhythmogenic (Chiang and Roden, 2000). Thus, FPD prolongation in SARS-CoV-2-infected hPSC-CMs may be an *in vitro* surrogate phenotype mirroring the arrhythmias observed in ~20% of COVID-19 patients (Malaty et al., 2020). Last, but not least, we found marked impairment in both contractile function and histological organization in 3D-EHTs infected with SARS-CoV-2. If similar effects were to occur in the hearts of some COVID-19 patients, this could contribute to cardiac dysfunction. Overall, hPSC-CMs on MEAs and/or organized in 3D-EHTs may represent valuable scalable platforms to identify active compounds that may provide therapeutic value.

Collectively, our results support the notion that, independent of inflammation or coagulopathy, SARS-CoV-2 can cause direct functional heart damage by either inducing cell death and/or by impairing electro-mechanical functions. One limitation of this study is our reliance on hPSC-CMs, which are well known for their functional immaturity (Guo and Pu, 2020; Karbassi et al., 2020; Marchiano et al., 2019). While the *in vitro* systems we used have been successfully leveraged to model electrophysiological and contractile alterations due to drugs or inherited mutations (Blinova et al., 2018; Paik et al., 2020), their application to modeling COVID-19 still requires further validation.

± SEM of eight and six wells for spontaneously beating and paced cells, respectively. Statistical analyses of intra-experimental variability as for (B) (**$p < 0.01$).

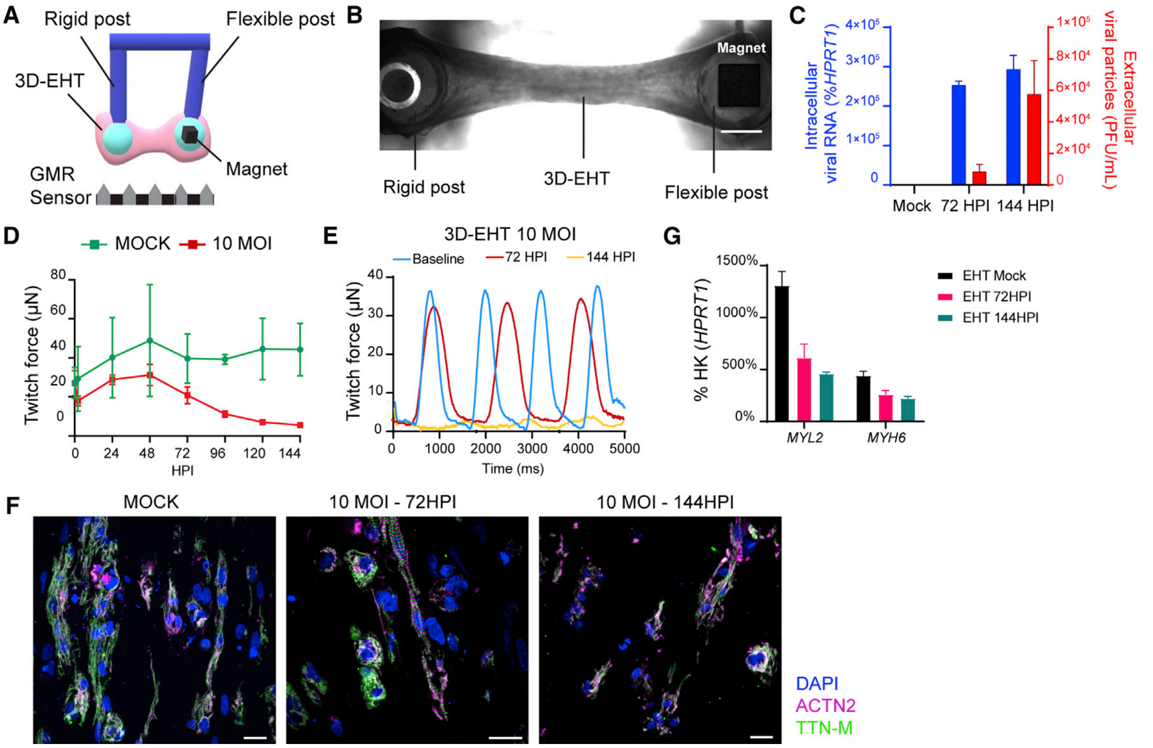


**Figure 6. Force Production of 3D-EHTs Made from hiPSC-CMs Progressively Declines during SARS-CoV-2 Infection**

(A) Schematic representation of the magnetic sensing system: the 3D-EHT is suspended between two posts (one rigid, one flexible). The magnet is localized inside the flexible post, and the post movement during 3D-EHT contraction is recorded by giant magnetoresistance (GMR) sensor located at the bottom of the dish (converting displacement into voltage changes).

(B) Representative picture of a 3D-EHT. Scale bar, 1 mm.

(C) SARS-CoV-2 viral RNA and particles detected in 3D-EHTs infected at 10 MOI at specified time points. Intracellular viral RNA is plotted on the right axis as percent *HPRT1*; extracellular viral particles are plotted on the right axis as PFU/mL. Mean ± SEM of two 3D-EHTs per condition.

(D) Representative time course analysis of twitch force in 3D-EHTs from WTC11c hiPSC-CMs after SARS-CoV-2 infection at 10 MOI. Data are shown as mean ± SEM for two mock controls and four infected 3D-EHTs.

(E) Representative twitch traces of 3D-EHT at 10 MOI at different time points.

(F and G) (F) Immunofluorescent images of 3D-EHTs sections at different time points. Scale bars, 20 μm. (G) qRT-PCR of sarcomeric genes in 3D-EHTs infected with SARS-CoV-2 at 10 MOI. Mean ± SEM of two 3D-EHTs.

Nevertheless, a recent report by Dolhnikoff et al. (2020) identified coronaviral particles in the cytoplasm of cardiomyocytes, endothelial cells, and fibroblasts by electron microscopy in the heart of an 11-year-old child who died from multi-system inflammatory syndrome in children following COVID-19 infection. This indicates that *in vivo* cardiomyocytes with substantially greater maturity than used here are susceptible to SARS-CoV-2 infection. COVID-19 patients are commonly treated with steroids to control systemic inflammation. However, our data suggest that treatments aimed to control the direct damage of SARS-CoV-2, not only by preventing infection but also by preventing viral replication or rescuing cardiac function, should also be taken into consideration to prevent long-term cardiovascular complications.

## EXPERIMENTAL PROCEDURES

### Cell Culture

RUES2 hESCs and WTC11c hiPSCs were maintained and differentiated using small-molecule modulators of the WNT pathway (Bertero et al., 2019a). H7 hESCs were differentiated in suspension culture format by collaborators at the Center for Applied Technology Development at the City of Hope in California. H9 hESCs were maintained and differentiated into lateral mesoderm- and neural crest-derived SMCs as described previously (Bargehr et al., 2016; Serrano et al., 2019). ACE2 KO clones were generated using CRISPR-Cas9 ribonucleoprotein complexes (Synthego).

### Gene Expression Analysis

Bulk RNA-seq datasets from RUES2 hESC-CMs had been previously generated and analyzed (Bertero et al., 2019a). Bulk mRNA-seq data

from infected cardiomyocytes were generated by constructing mRNA-seq libraries using the KAPA mRNA HyperPrep Kit (Kapa Biosystems). Libraries were sequenced on an Illumina NovaSeq. For single-cell RNA-seq analysis, a single-cell suspension was generated from RUES2 hESC-CMs and single-cell RNA-seq was performed using the Chromium Next GEM Single Cell 3′ Kit (10X Genomics). For real-time qRT-PCR, RNA from infected cardiomyocytes was harvested using TRIzol reagent. cDNA was obtained with M-MLV reverse transcriptase (Invitrogen), and qRT-PCR was performed with SYBR Select Master Mix (Applied Biosystems). Primers are reported in Table S5.

### Western Blot

hPSC-CMs were lysed using RIPA Buffer. Samples were run on mini-PROTEAN TGX precast gels (Bio-Rad) and then transferred onto polyvinylidene fluoride membranes. Primary and secondary antibodies (Supplemental Experimental Procedures) were incubated in blocking buffer, and fluorescent signals were acquired using a GelDoc Imager (Bio-Rad).

### SARS-CoV-2 Generation

All experiments using live virus were performed in the Biosafety Level 3 (BSL-3) facility at the University of Washington in compliance with the BSL-3 laboratory safety protocols (CDC BMBL 5$^{th}$ ed.) and the recent CDC guidelines for handling SARS-CoV-2. SARS-related coronavirus 2, Isolate USA-WA1/2020 (SARS-CoV-2) and icSARS-CoV-2mNG were obtained from BEI Resources (NR-52281) and the University of Texas (Xie et al., 2020), respectively, and propagated in VERO cells (USAMRIID). Viral preparations and culture supernatant from SARS-CoV-2-infected cardiomyocytes were titered using a plaque assay.

### Viral Infection

SARS-CoV-2 wild-type or expressing mNeonGreen protein was diluted to the desired MOI in DMEM and incubated on hPSC-CMs or hPSC-SMCs for 1 h at 37°C (non-infected [mock] controls were incubated with DMEM only). Cells were then washed with Dulbecco's Phosphate-buffered Saline (DPBS) and cultured in the appropriate maintenance media.

### Immunofluorescence

hPSC-CMs were fixed with 4% paraformaldehyde (PFA) and permeabilized using 0.25% Triton X-100 (Sigma-Aldrich) before staining with primary and secondary antibodies (Supplemental Experimental Procedures).

### Electron Microscopy

hPSC-CMs were fixed with Karnovsky's fixative. Heavy metal impregnation was performed as detailed elsewhere (Deerinck et al., 2010). Thin sections were viewed using a JEOL 1230 transmission electron microscope.

### Electrophysiological Analysis with MEA

CytoView MEA plates (Axion BioSystems) were coated with Matrigel and hPSC-CMs were plated as described previously (Bertero et al., 2019b). SARS-CoV-2 effects on hPSC-CMs electrophysiology were recorded using the Maestro Pro system and analyzed with Cardiac Analysis Software v.3.1.8 (all from Axion BioSystems).

### 3D-EHTs Analysis

Post-suspended, fibrin-based 3D-EHTs were generated with hPSC-CMs and HS27a stromal cells (ATCC) at a 1:10 ratio. Twitch force was recorded by tracking the movement of magnets embedded in the flexible posts, as described previously (Bielawski et al., 2016). For immunostaining, 3D-EHTs were arrested in diastole, fixed in 4% PFA, and embedded in Tissue-Tek O.C.T. before sectioning and staining.

### Statistical Analyses

Statistical analyses were performed only for experiments with more than two replicates using Prism 8.1.3 (GraphPad). The type and number of replicates, the statistics plotted, the statistical test used, and the test results are described in the figure legends.

### Data and Code Availability

The data supporting this publication have been made available in GEO under accession numbers: GSE157175 (single-cell RNA-seq) and GSE162736 (bulk RNA-seq).

## SUPPLEMENTAL INFORMATION

Supplemental Information can be found online at https://doi.org/10.1016/j.stemcr.2021.02.008. More extensive descriptions of all methods and reagents are included in the Supplemental Experimental Procedures section.

## AUTHOR CONTRIBUTIONS

S.M. performed hiPSC-CM differentiation, viral infections, western blots, viral plaque, MEA and 3D-EHTs assays, and wrote the first draft of the manuscript. T.-Y.H. cultured and expanded SARS-CoV-2, performed immunofluorescence and collected samples for RNA-seq. A.K. generated and characterized *ACE2* KO cell lines and performed qRT-PCR. T.H. designed and fabricated the 3D-EHT magnetic sensing system, and analyzed the data. L.S.W., J.C., and E.S. performed library preparation and RNA-seq data analysis. J.B., H.D., M.C., and L.P.O. differentiated hPSC-SMCs and contributed to experimental design. H.R. contributed to experimental design and differentiated hiPSC-CMs. X.Y. contributed to experimental design and analyzed both bulk and single-cell RNA-seq data. L.P. and S.S. contributed to experimental design and supervised the experiments. B.N. performed electron microscopy and contributed to data analysis and writing the manuscript. A.B. contributed to the study conception, experimental design and execution, and assisted in writing the manuscript. N.J.S., M.G., and C.E.M. conceived and supervised the study, obtained research funding, and contributed to data analysis and writing the manuscript.

## ACKNOWLEDGMENTS

We thank Drs. Farid Moussavi-Harami, Kenta Nakamura, and Daniel Yang for helpful discussions during the development of this project, and Dr. Aidan Fenix for experimental support. We are grateful to Axion BioSystems for proving material and

assistance for the MEA experiments. This research was assisted by the University of Washington Cell Analysis Facility (Department of Immunology), the Lynn and Mike Garvey Cell Imaging Core (Institute for Stem Cell and Regenerative Medicine), and the Vision Core Facility laboratory. We would also like to acknowledge the staff of the University of Washington BSL-3 facility, as well as our Environmental Health and Safety staff, who provided assistance during experiments and ensured our safety. S.M. is supported by a Postdoctoral Fellowship from the UW Institute for Stem Cell and Regenerative Medicine. This work was supported in part by the National Science Foundation (CMMI-166173 to N.J.S.), NIH (R01 HL149734 to N.J.S.; R01 AI118916, R01 AI127463, R01 AI145296, R21 AI145359, U01 AI151698, UM1 AI148684, and U19 AI100625 to M.G.; R01 HL128362 and R01 HL146868 to C.E.M.), the British Heart Foundation (FS/18/46/33663, RG/17/5/32936, and RM/17/2/33380 to S.S.), the Robert B. McMillen Foundation and from the State of Washington and philanthropical support to the UW Institute for Stem Cell and Regenerative Medicine.

Received: January 18, 2021
Revised: February 8, 2021
Accepted: February 9, 2021
Published: March 9, 2021

## REFERENCES

Ajaz, S., McPhail, M.J., Singh, K.K., Mujib, S., Trovato, F.M., Napoli, S., and Agarwal, K. (2021). Mitochondrial metabolic manipulation by SARS-CoV-2 in peripheral blood mononuclear cells of patients with COVID-19. Am. J. Physiol. Cell Physiol. *320*, C57–C65.

Alhogbani, T. (2016). Acute myocarditis associated with novel Middle East respiratory syndrome coronavirus. Ann. Saudi Med. *36*, 78–80.

Andersen, K.G., Rambaut, A., Lipkin, W.I., Holmes, E.C., and Garry, R.F. (2020). The proximal origin of SARS-CoV-2. Nat. Med. *26*, 450–452.

Arentz, M., Yim, E., Klaff, L., Lokhandwala, S., Riedo, F.X., Chong, M., and Lee, M. (2020). Characteristics and outcomes of 21 critically ill patients with COVID-19 in Washington State. JAMA *323*, 1612–1614.

Baggiano, A., Rizzo, S., Basso, C., and Pontone, G. (2020). A patient with rapid worsening dyspnoea during Covid-19 pandemic. Eur. Heart J. https://doi.org/10.1093/eurheartj/ehaa988.

Banerjee, A.K., Blanco, M.R., Bruce, E.A., Honson, D.D., Chen, L.M., Chow, A., Bhat, P., Ollikainen, N., Quinodoz, S.A., Loney, C., et al. (2020). SARS-CoV-2 disrupts splicing, translation, and protein trafficking to suppress host defenses. Cell *183*, 1325–1339.e1.

Bargehr, J., Low, L., Cheung, C., Bernard, W.G., Iyer, D., Bennett, M.R., Gambardella, L., and Sinha, S. (2016). Embryological origin of human smooth muscle cells influences their ability to support endothelial network formation. Stem Cells Transl. Med. *5*, 946–959.

Bertero, A., Fields, P.A., Ramani, V., Bonora, G., Yardimci, G.G., Reinecke, H., Pabon, L., Noble, W.S., Shendure, J., and Murry, C.E. (2019a). Dynamics of genome reorganization during human cardiogenesis reveal an RBM20-dependent splicing factory. Nat. Commun. *10*, 1538.

Bertero, A., Fields, P.A., Smith, A.S.T., Leonard, A., Beussman, K., Sniadecki, N.J., Kim, D.H., Tse, H.F., Pabon, L., Shendure, J., et al. (2019b). Chromatin compartment dynamics in a haploinsufficient model of cardiac laminopathy. J. Cell Biol. *218*, 2919–2944.

Bhatla, A., Mayer, M.M., Adusumalli, S., Hyman, M.C., Oh, E., Tierney, A., Moss, J., Chahal, A.A., Anesi, G., Denduluri, S., et al. (2020). COVID-19 and cardiac arrhythmias. Heart Rhythm *17*, 1439–1444.

Bielawski, K.S., Leonard, A., Bhandari, S., Murry, C.E., and Sniadecki, N.J. (2016). Real-time force and frequency analysis of engineered human heart tissue derived from induced pluripotent stem cells using magnetic sensing. Tissue Eng. Part C Methods *22*, 932–940.

Blinova, K., Dang, Q., Millard, D., Smith, G., Pierson, J., Guo, L., Brock, M., Lu, H.R., Kraushaar, U., Zeng, H., et al. (2018). International multisite study of human-induced pluripotent stem cell-derived cardiomyocytes for drug proarrhythmic potential assessment. Cell Rep. *24*, 3582–3592.

Bojkova, D., Wagner, J.U.G., Shumliakivska, M., Aslan, G.S., Saleem, U., Hansen, A., Luxan, G., Gunther, S., Pham, M.D., Krishnan, J., et al. (2020). SARS-CoV-2 infects and induces cytotoxic effects in human cardiomyocytes. Cardiovasc. Res. *116*, 2207–2215.

Bradley, B.T., Maioli, H., Johnston, R., Chaudhry, I., Fink, S.L., Xu, H., Najafian, B., Deutsch, G., Lacy, J.M., Williams, T., et al. (2020). Histopathology and ultrastructural findings of fatal COVID-19 infections in Washington State: a case series. Lancet *396*, 320–332.

Chen, L., Li, X., Chen, M., Feng, Y., and Xiong, C. (2020a). The ACE2 expression in human heart indicates new potential mechanism of heart injury among patients infected with SARS-CoV-2. Cardiovasc. Res. *116*, 1097–1100.

Chen, S., Yang, L., Nilsson-Payant, B., Han, Y., Jaffre, F., Zhu, J., Wang, P., Zhang, T., Redmond, D., Houghton, S., et al. (2020b). SARS-CoV-2 infected cardiomyocytes recruit monocytes by secreting CCL2. Res. Sq. https://doi.org/10.21203/rs.3.rs-94634/v1.

Cheung, C., Bernardo, A.S., Trotter, M.W., Pedersen, R.A., and Sinha, S. (2012). Generation of human vascular smooth muscle subtypes provides insight into embryological origin-dependent disease susceptibility. Nat. Biotechnol. *30*, 165–173.

Chiang, C.E., and Roden, D.M. (2000). The long QT syndromes: genetic basis and clinical implications. J. Am. Coll. Cardiol. *36*, 1–12.

Corman, V.M., Muth, D., Niemeyer, D., and Drosten, C. (2018). Hosts and sources of endemic human coronaviruses. Adv. Virus Res. *100*, 163–188.

Cui, J., Li, F., and Shi, Z.L. (2019). Origin and evolution of pathogenic coronaviruses. Nat. Rev. Microbiol. *17*, 181–192.

Deerinck, T., Bushong, E., Lev-Ram, V., Shu, X., Tsien, R., and Ellisman, M. (2010). Enhancing serial block-face scanning electron microscopy to enable high resolution 3-D nanohistology of cells and tissues. Microsc. Microanal. *16*, 1138–1139.

Dolhnikoff, M., Ferreira Ferranti, J., de Almeida Monteiro, R.A., Duarte-Neto, A.N., Soares Gomes-Gouvea, M., Viu Degaspare, N., Figueiredo Delgado, A., Montanari Fiorita, C., Nunes Leal, G., Rodrigues, R.M., et al. (2020). SARS-CoV-2 in cardiac tissue of a child with COVID-19-related multisystem inflammatory syndrome. Lancet Child Adolesc. Health *4*, 790–794.

Dong, N., Cai, J., Zhou, Y., Liu, J., and Li, F. (2020). End-stage heart failure with COVID-19: strong evidence of myocardial injury by 2019-nCoV. JACC Heart Fail. *8*, 515–517.

Driggin, E., Madhavan, M.V., Bikdeli, B., Chuich, T., Laracy, J., Biondi-Zoccai, G., Brown, T.S., Der Nigoghossian, C., Zidar, D.A., Haythe, J., et al. (2020). Cardiovascular considerations for patients, health care workers, and health systems during the COVID-19 pandemic. J. Am. Coll. Cardiol. *75*, 2352–2371.

Ghosh, S., Dellibovi-Ragheb, T.A., Kerviel, A., Pak, E., Qiu, Q., Fisher, M., Takvorian, P.M., Bleck, C., Hsu, V.W., Fehr, A.R., et al. (2020). Beta-Coronaviruses use lysosomes for egress instead of the biosynthetic secretory pathway. Cell *183*, 1520–1535.e14.

Guo, T., Fan, Y., Chen, M., Wu, X., Zhang, L., He, T., Wang, H., Wan, J., Wang, X., and Lu, Z. (2020). Cardiovascular implications of fatal outcomes of patients with coronavirus disease 2019 (COVID-19). JAMA Cardiol. *5*, 811–818.

Guo, Y., and Pu, W.T. (2020). Cardiomyocyte maturation: new phase in development. Circ. Res. *126*, 1086–1106.

Hikmet, F., Mear, L., Edvinsson, A., Micke, P., Uhlen, M., and Lindskog, C. (2020). The protein expression profile of ACE2 in human tissues. Mol. Syst. Biol. *16*, e9610.

Hoffmann, M., Kleine-Weber, H., Schroeder, S., Kruger, N., Herrler, T., Erichsen, S., Schiergens, T.S., Herrler, G., Wu, N.H., Nitsche, A., et al. (2020). SARS-CoV-2 cell entry depends on ACE2 and TMPRSS2 and is blocked by a clinically proven protease inhibitor. Cell *181*, 271–280.e8.

Hopkins, J. (2020). Cronavirus disease 2019 (COVID-19) situation map. https://coronavirus.jhu.edu/map.html.

Huang, C., Wang, Y., Li, X., Ren, L., Zhao, J., Hu, Y., Zhang, L., Fan, G., Xu, J., Gu, X., et al. (2020a). Clinical features of patients infected with 2019 novel coronavirus in Wuhan, China. Lancet *395*, 497–506.

Huang, L., Zhao, P., Tang, D., Zhu, T., Han, R., Zhan, C., Liu, W., Zeng, H., Tao, Q., and Xia, L. (2020b). Cardiac involvement in patients recovered from COVID-2019 identified using magnetic resonance imaging. JACC Cardiovasc. Imaging *13*, 2330–2339.

Icard, P., Lincet, H., Wu, Z., Coquerel, A., Forgez, P., Alifano, M., and Fournel, L. (2021). The key role of Warburg effect in SARS-CoV-2 replication and associated inflammatory response. Biochimie *180*, 169–177.

Inciardi, R.M., Lupi, L., Zaccone, G., Italia, L., Raffo, M., Tomasoni, D., Cani, D.S., Cerini, M., Farina, D., Gavazzi, E., et al. (2020). Cardiac involvement in a patient with coronavirus disease 2019 (COVID-19). JAMA Cardiol. *5*, 819–824.

Kang, Y.L., Chou, Y.Y., Rothlauf, P.W., Liu, Z., Soh, T.K., Cureton, D., Case, J.B., Chen, R.E., Diamond, M.S., Whelan, S.P.J., et al. (2020). Inhibition of PIKfyve kinase prevents infection by Zaire ebolavirus and SARS-CoV-2. Proc. Natl. Acad. Sci. U S A *117*, 20803–20813.

Karbassi, E., Fenix, A., Marchiano, S., Muraoka, N., Nakamura, K., Yang, X., and Murry, C.E. (2020). Cardiomyocyte maturation: advances in knowledge and implications for regenerative medicine. Nat. Rev. Cardiol. *17*, 341–359.

Lei, X., Dong, X., Ma, R., Wang, W., Xiao, X., Tian, Z., Wang, C., Wang, Y., Li, L., Ren, L., et al. (2020). Activation and evasion of type I interferon responses by SARS-CoV-2. Nat. Commun. *11*, 3810.

Li, Y., Zhou, W., Yang, L., and You, R. (2020). Physiological and pathological regulation of ACE2, the SARS-CoV-2 receptor. Pharmacol. Res. *157*, 104833.

Lindner, D., Fitzek, A., Brauninger, H., Aleshcheva, G., Edler, C., Meissner, K., Scherschel, K., Kirchhof, P., Escher, F., Schultheiss, H.P., et al. (2020). Association of cardiac infection with SARS-CoV-2 in confirmed COVID-19 autopsy cases. JAMA Cardiol. *5*, 1281–1285.

Litvinukova, M., Talavera-Lopez, C., Maatz, H., Reichart, D., Worth, C.L., Lindberg, E.L., Kanda, M., Polanski, K., Heinig, M., Lee, M., et al. (2020). Cells of the adult human heart. Nature *588*, 466–472.

Loo, Y.M., and Gale, M., Jr. (2011). Immune signaling by RIG-I-like receptors. Immunity *34*, 680–692.

Madjid, M., Miller, C.C., Zarubaev, V.V., Marinich, I.G., Kiselev, O.I., Lobzin, Y.V., Filippov, A.E., and Casscells, S.W., 3rd. (2007). Influenza epidemics and acute respiratory disease activity are associated with a surge in autopsy-confirmed coronary heart disease death: results from 8 years of autopsies in 34,892 subjects. Eur. Heart J. *28*, 1205–1210.

Malaty, M., Kayes, T., Amarasekera, A.T., Kodsi, M., MacIntyre, C.R., and Tan, T.C. (2020). Incidence and treatment of arrhythmias secondary to coronavirus infection in humans: a systematic review. Eur. J. Clin. Invest. *51*, e13428.

Marchiano, S., Bertero, A., and Murry, C.E. (2019). Learn from your elders: developmental biology lessons to guide maturation of stem cell-derived cardiomyocytes. Pediatr. Cardiol. *40*, 1367–1387.

Millet, J.K., and Whittaker, G.R. (2014). Host cell entry of Middle East respiratory syndrome coronavirus after two-step, furin-mediated activation of the spike protein. Proc. Natl. Acad. Sci. U S A *111*, 15214–15219.

Millet, J.K., and Whittaker, G.R. (2015). Host cell proteases: critical determinants of coronavirus tropism and pathogenesis. Virus Res. *202*, 120–134.

Nishiga, M., Wang, D.W., Han, Y., Lewis, D.B., and Wu, J.C. (2020). COVID-19 and cardiovascular disease: from basic mechanisms to clinical perspectives. Nat. Rev. Cardiol. *17*, 543–558.

Ojha, V., Verma, M., Pandey, N.N., Mani, A., Malhi, A.S., Kumar, S., Jagia, P., Roy, A., and Sharma, S. (2020). Cardiac magnetic resonance imaging in coronavirus disease 2019 (COVID-19): a systematic review of cardiac magnetic resonance imaging findings in 199 patients. J. Thorac. Imaging https://doi.org/10.1097/RTI.0000000000000574.

Ou, X., Liu, Y., Lei, X., Li, P., Mi, D., Ren, L., Guo, L., Guo, R., Chen, T., Hu, J., et al. (2020). Characterization of spike glycoprotein of SARS-CoV-2 on virus entry and its immune cross-reactivity with SARS-CoV. Nat. Commun. *11*, 1620.

Oudit, G.Y., Kassiri, Z., Jiang, C., Liu, P.P., Poutanen, S.M., Penninger, J.M., and Butany, J. (2009). SARS-coronavirus modulation of myocardial ACE2 expression and inflammation in patients with SARS. Eur. J. Clin. Invest. *39*, 618–625.

Paik, D.T., Chandy, M., and Wu, J.C. (2020). Patient and disease-specific induced pluripotent stem cells for discovery of

personalized cardiovascular drugs and therapeutics. Pharmacol. Rev. *72*, 320–342.

Puntmann, V.O., Carerj, M.L., Wieters, I., Fahim, M., Arendt, C., Hoffmann, J., Shchendrygina, A., Escher, F., Vasa-Nicotera, M., Zeiher, A.M., et al. (2020). Outcomes of cardiovascular magnetic resonance imaging in patients recently recovered from coronavirus disease 2019 (COVID-19). JAMA Cardiol. *5*, 1265–1273.

Rajpal, S., Tong, M.S., Borchers, J., Zareba, K.M., Obarski, T.P., Simonetti, O.P., and Daniels, C.J. (2021). Cardiovascular magnetic resonance findings in competitive athletes recovering from COVID-19 infection. JAMA Cardiol. *6*, 116–118.

Ranucci, M., Ballotta, A., Di Dedda, U., Bayshnikova, E., Dei Poli, M., Resta, M., Falco, M., Albano, G., and Menicanti, L. (2020). The procoagulant pattern of patients with COVID-19 acute respiratory distress syndrome. J. Thromb. Haemost. *18*, 1747–1751.

Ruan, Q., Yang, K., Wang, W., Jiang, L., and Song, J. (2020). Clinical predictors of mortality due to COVID-19 based on an analysis of data of 150 patients from Wuhan, China. Intensive Care Med. *46*, 846–848.

Schneider, J.P.,D., Navaratnarajah, C., Halfmann, P., Clemens, D., Ye, D., Changsung, K., Barkhymer, A., Cohle, S., Banks, A., Mehta, A., et al. (2020). SARS-CoV-2 direct cardiac damage through spike-mediated cardiomyocyte fusion. Res. Sq. https://doi.org/10.21203/rs.3.rs-95587/v1.

Serrano, F., Bernard, W.G., Granata, A., Iyer, D., Steventon, B., Kim, M., Vallier, L., Gambardella, L., and Sinha, S. (2019). A novel human pluripotent stem cell-derived neural crest model of Treacher Collins syndrome shows defects in cell death and migration. Stem Cells Dev. *28*, 81–100.

Shang, J., Wan, Y., Luo, C., Ye, G., Geng, Q., Auerbach, A., and Li, F. (2020). Cell entry mechanisms of SARS-CoV-2. Proc. Natl. Acad. Sci. U S A *117*, 11727–11734.

Sharma, A., Garcia, G., Jr., Wang, Y., Plummer, J.T., Morizono, K., Arumugaswami, V., and Svendsen, C.N. (2020). Human iPSC-derived cardiomyocytes are susceptible to SARS-CoV-2 infection. Cell Rep. Med. *1*, 100052.

Shi, S., Qin, M., Shen, B., Cai, Y., Liu, T., Yang, F., Gong, W., Liu, X., Liang, J., Zhao, Q., et al. (2020). Association of cardiac injury with mortality in hospitalized patients with COVID-19 in Wuhan, China. JAMA Cardiol. *5*, 802–810.

Shulla, A., Heald-Sargent, T., Subramanya, G., Zhao, J., Perlman, S., and Gallagher, T. (2011). A transmembrane serine protease is linked to the severe acute respiratory syndrome coronavirus receptor and activates virus entry. J. Virol. *85*, 873–882.

Snijder, E.J., Limpens, R., de Wilde, A.H., de Jong, A.W.M., Zevenhoven-Dobbe, J.C., Maier, H.J., Faas, F., Koster, A.J., and Barcena, M. (2020). A unifying structural and functional model of the coronavirus replication organelle: tracking down RNA synthesis. PLoS Biol. *18*, e3000715.

Tay, M.Z., Poh, C.M., Renia, L., MacAry, P.A., and Ng, L.F.P. (2020). The trinity of COVID-19: immunity, inflammation and intervention. Nat. Rev. Immunol. *20*, 363–374.

Verdecchia, P., Cavallini, C., Spanevello, A., and Angeli, F. (2020). The pivotal link between ACE2 deficiency and SARS-CoV-2 infection. Eur. J. Intern. Med. *76*, 14–20.

Wang, D., Hu, B., Hu, C., Zhu, F., Liu, X., Zhang, J., Wang, B., Xiang, H., Cheng, Z., Xiong, Y., et al. (2020). Clinical characteristics of 138 hospitalized patients with 2019 novel coronavirus-infected pneumonia in Wuhan, China. JAMA *323*, 1061–1069.

Wolff, G., Melia, C.E., Snijder, E.J., and Barcena, M. (2020). Double-membrane vesicles as platforms for viral replication. Trends Microbiol. *28*, 1022–1033.

Xia, S., Lan, Q., Su, S., Wang, X., Xu, W., Liu, Z., Zhu, Y., Wang, Q., Lu, L., and Jiang, S. (2020a). The role of furin cleavage site in SARS-CoV-2 spike protein-mediated membrane fusion in the presence or absence of trypsin. Signal Transduct. Target. Ther. *5*, 92.

Xia, S., Liu, M., Wang, C., Xu, W., Lan, Q., Feng, S., Qi, F., Bao, L., Du, L., Liu, S., et al. (2020b). Inhibition of SARS-CoV-2 (previously 2019-nCoV) infection by a highly potent pan-coronavirus fusion inhibitor targeting its spike protein that harbors a high capacity to mediate membrane fusion. Cell Res. *30*, 343–355.

Xie, X., Muruato, A., Lokugamage, K.G., Narayanan, K., Zhang, X., Zou, J., Liu, J., Schindewolf, C., Bopp, N.E., Aguilar, P.V., et al. (2020). An infectious cDNA clone of SARS-CoV-2. Cell Host Microbe *27*, 841–848.e3.

Xu, H., Hou, K., Xu, R., Li, Z., Fu, H., Wen, L., Xie, L., Liu, H., Selvanayagam, J.B., Zhang, N., et al. (2020). Clinical characteristics and risk factors of cardiac involvement in COVID-19. J. Am. Heart Assoc. *9*, e016807.

Yang, K.C., Breitbart, A., De Lange, W.J., Hofsteen, P., Futakuchi-Tsuchida, A., Xu, J., Schopf, C., Razumova, M.V., Jiao, A., Boucek, R., et al. (2018). Novel adult-onset systolic cardiomyopathy due to MYH7 E848G mutation in patient-derived induced pluripotent stem cells. JACC Basic Transl. Sci. *3*, 728–740.

Yang, L., Han, Y., Nilsson-Payant, B.E., Gupta, V., Wang, P., Duan, X., Tang, X., Zhu, J., Zhao, Z., Jaffre, F., et al. (2020). A human pluripotent stem cell-based platform to study SARS-CoV-2 tropism and model virus infection in human cells and organoids. Cell Stem Cell *27*, 125–136.e7.

Yang, N., and Shen, H.M. (2020). Targeting the endocytic pathway and autophagy process as a novel therapeutic strategy in COVID-19. Int. J. Biol. Sci. *16*, 1724–1731.

Zhou, F., Yu, T., Du, R., Fan, G., Liu, Y., Liu, Z., Xiang, J., Wang, Y., Song, B., Gu, X., et al. (2020). Clinical course and risk factors for mortality of adult inpatients with COVID-19 in Wuhan, China: a retrospective cohort study. Lancet *395*, 1054–1062.

Zhu, N., Zhang, D., Wang, W., Li, X., Yang, B., Song, J., Zhao, X., Huang, B., Shi, W., Lu, R., et al. (2020). A novel coronavirus from patients with pneumonia in China, 2019. N. Engl. J. Med. *382*, 727–733.

Zumla, A., Chan, J.F., Azhar, E.I., Hui, D.S., and Yuen, K.Y. (2016). Coronaviruses—drug discovery and therapeutic options. Nat. Rev. Drug Discov. *15*, 327–347.

Zylla, M.M., Merle, U., Vey, J.A., Korosoglou, G., Hofmann, E., Muller, M., Herth, F., Schmidt, W., Blessing, E., Goggelmann, C., et al. (2021). Predictors and prognostic implications of cardiac arrhythmias in patients hospitalized for COVID-19. J. Clin. Med. *10*, 133.

Stem Cell Reports, Volume *16*

# Supplemental Information

# SARS-CoV-2 Infects Human Pluripotent Stem Cell-Derived Cardiomyocytes, Impairing Electrical and Mechanical Function

**Silvia Marchiano, Tien-Ying Hsiang, Akshita Khanna, Ty Higashi, Leanne S. Whitmore, Johannes Bargehr, Hongorzul Davaapil, Jean Chang, Elise Smith, Lay Ping Ong, Maria Colzani, Hans Reinecke, Xiulan Yang, Lil Pabon, Sanjay Sinha, Behzad Najafian, Nathan J. Sniadecki, Alessandro Bertero, Michael Gale Jr., and Charles E. Murry**

# Supplementary Information

**Supplementary Figure 1 – hPSC-CMs express SARS-CoV-2 receptors and processing factors.**

**Supplementary Figure 2 – hPSC-CMs are permissive to SARS-CoV-2 infection and replication.**

**Supplementary Figure 3 – Genotyping and validation of WTC11c hiPSC *ACE2* knockout clones.**

**Supplementary Figure 4 – Gene expression analysis of WTC11c-CMs infected with SARS-CoV-2.**

**Supplementary Figure 5 – Electrophysiological alterations in hPSC-CMs infected with SARS-CoV-2.**

**Supplementary Figure 6 – WTC11c hiPSC-CMs 3D-EHTs infected with SARS-CoV-2.**

**Supplementary Table 1 – Extended data from GO analysis (upregulated pathways for H7-CMs).**

**Supplementary Table 2 – Extended data from GO analysis (downregulated pathways for H7-CMs).**

**Supplementary Table 3 – Extended data from GO analysis (upregulated pathways for WTC11c-CMs).**

**Supplementary Table 4 – Extended data from GO analysis (downregulated pathways for WTC11c-CMs).**

**Supplementary Table 5 – RT-qPCR primer sequences.**

**Supplemental Experimental Procedures**

**Supplemental References**

**Supplementary Video 1 – 3D-EHT before SARS-CoV-2 infection (online).**

**Supplementary Video 2 – SARS-CoV-2 infected 3D-EHT at 144 HPI (online).**

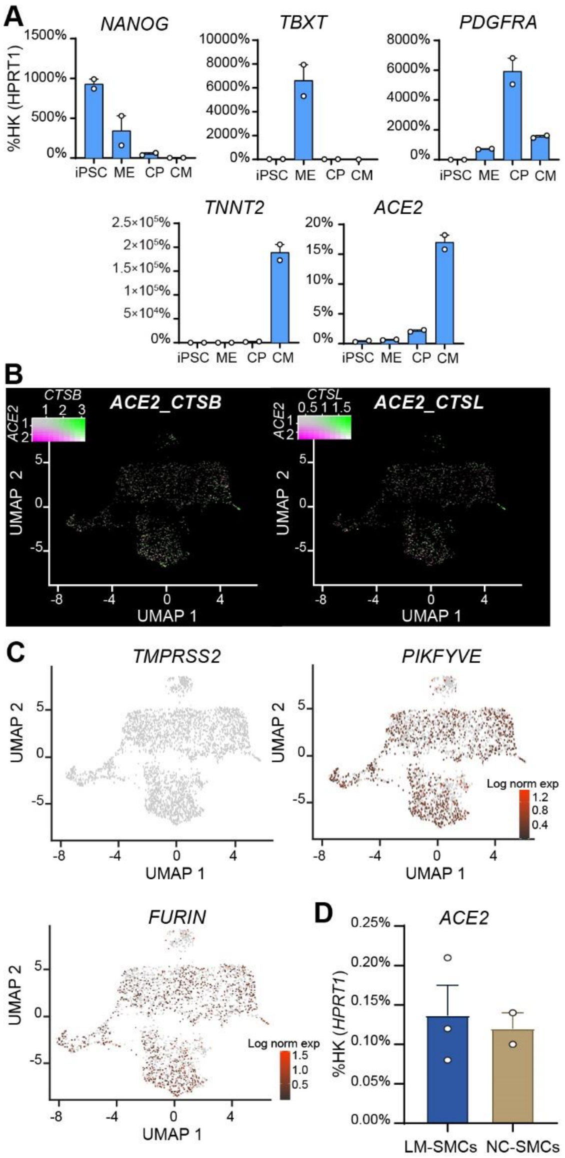


**Supplementary Figure 1 – hPSC-CMs express SARS-CoV-2 receptors and processing factors.** (**A**) RT-qPCR analysis during WTC11c hiPSC-CM differentiation (two independent experiments). iPSC: induced pluripotent stem cells (day 0); ME: mesoderm (day 2); CP: cardiac progenitors (day 5); CM: cardiomyocytes (day 14). Mean ± SEM of two independent experiments. (**B-C**) sc-RNA-seq gene expression heatmaps from RUES2 hESC-CMs after dimensionality reduction through UMAP. In B, plots showcase double positive cells for *ACE2* and *CTSL*, and *ACE2* and *CTSB*. Related to Figure 1B. (**D**) RT-qPCR analysis of H9 hESC-SMCs (LM: lateral plate mesoderm-derived; NC: neural crest-derived). Mean ± SEM of 3 differentiation batches. In one batch of NC-SMCs, *ACE2* was undetectable (Ct > 40). Related to Figure 1C.

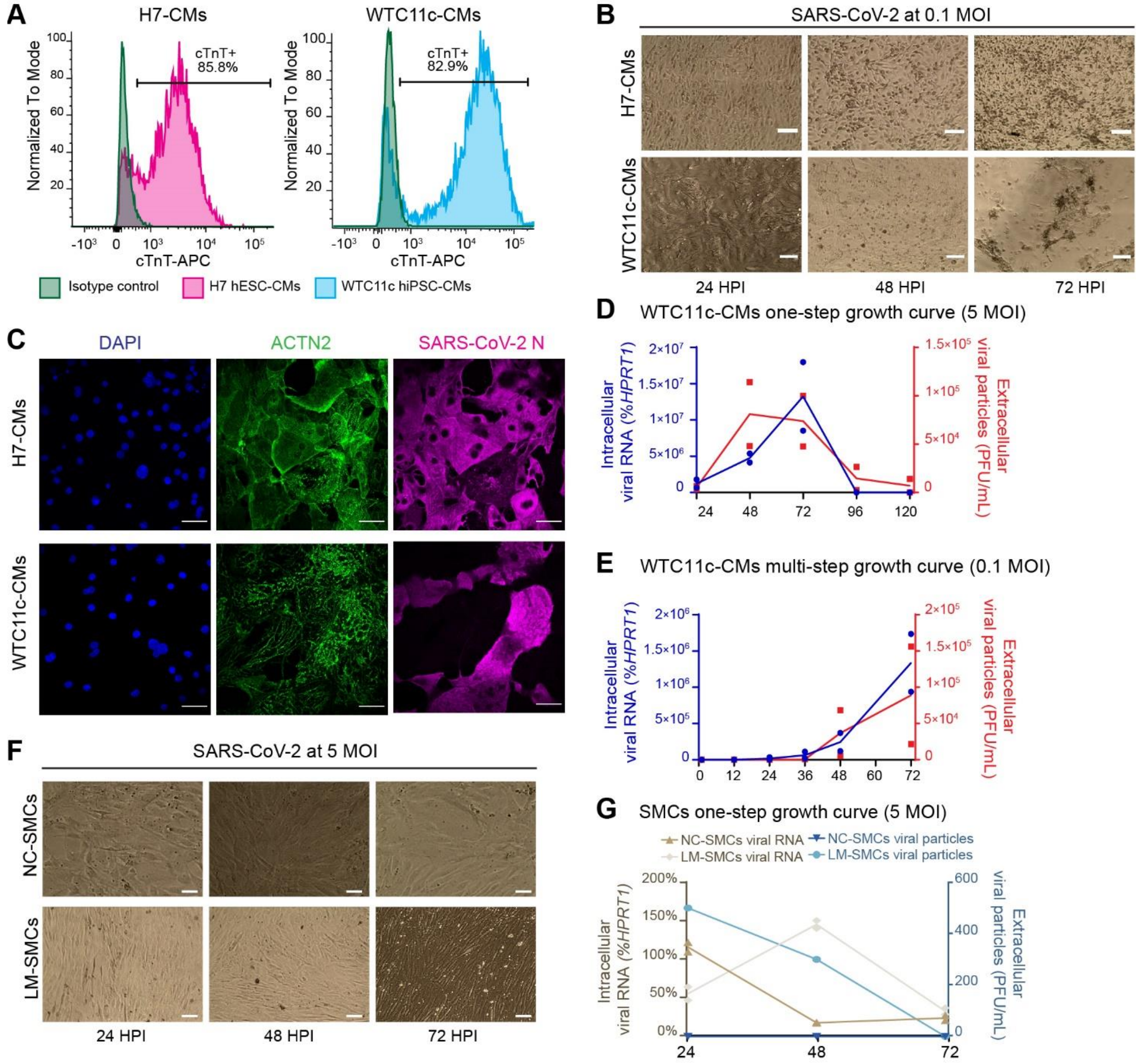


**Supplementary Figure 2 – hPSC-CMs are permissive to SARS-CoV-2 infection and replication.** (**A**) Representative flow cytometry analyses for cardiac troponin T (cTnT) expression in H7 hESC-CMs and WTC11c hiPSC-CMs. (**B**) Representative images of H7 hESC-CMs and WTC11c hiPSC-CMs infected with SARS-CoV-2 at 0.1 MOI during a time course of 72 h. Scale bars: 100 µm. Related to Figure 2A. (**C**) Single channel images for the immunostainings of SARS-CoV-2-infected hPSC-CMs shown in Figure 2B. (**D**) One-step viral growth curve in WTC11c hiPSC-CMs infected with SARS-CoV-2 at 5 MOI. (**E**) Multi-step viral growth curve in WTC11c hiPSC-CMs infected with SARS-CoV-2 at 0.1 MOI. For both D and E, lines connect the mean of two independent experiments. Viral RNA indicating intracellular viral replication is plotted on the left y axis as % of *HPRT1*. Viral particles secreted in the supernatant are plotted on the right y axis as PFU/mL. (**F**) Representative images of LM-SMCs and NC-SMCs infected with SARS-CoV-2 at 5 MOI during a time course of 72 h. Scale bars: 100 µm. (**G**) One-step viral growth curve in LM-SMCs and NC-SMCs infected with SARS-CoV-2 at 5 MOI. Viral RNA indicating intracellular viral replication is plotted on the left y axis as % of *HPRT1*. Plots as for panels D-E. Lines connect the means of two independent experiments.

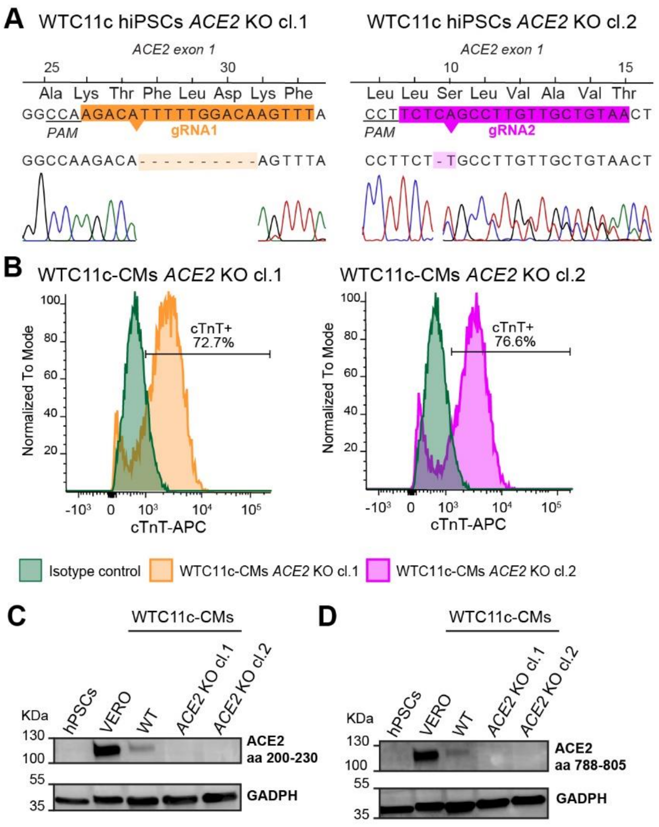


**Supplementary Figure 3 – Genotyping and validation of WTC11c hiPSC *ACE2* knockout clones. Related to Figures 2E, F.** (**A**) Genotyping by Sanger sequencing of WTC11c hiPSCs *ACE2* knockout (KO) clone 1 and clone 2. The location of sgRNAs protospacer sequences is highlighted. (**B**) Flow cytometry analyses of WTC11c-CMs *ACE2* KO clones. (**C-D**) Western blot for ACE2 N-terminal domain (C) and C-terminal domain (D) in WTC11c-CMs *ACE2* KO clones, confirming loss of full length ACE2 as well as of potential truncations (no bands detected at a lower molecular weight).

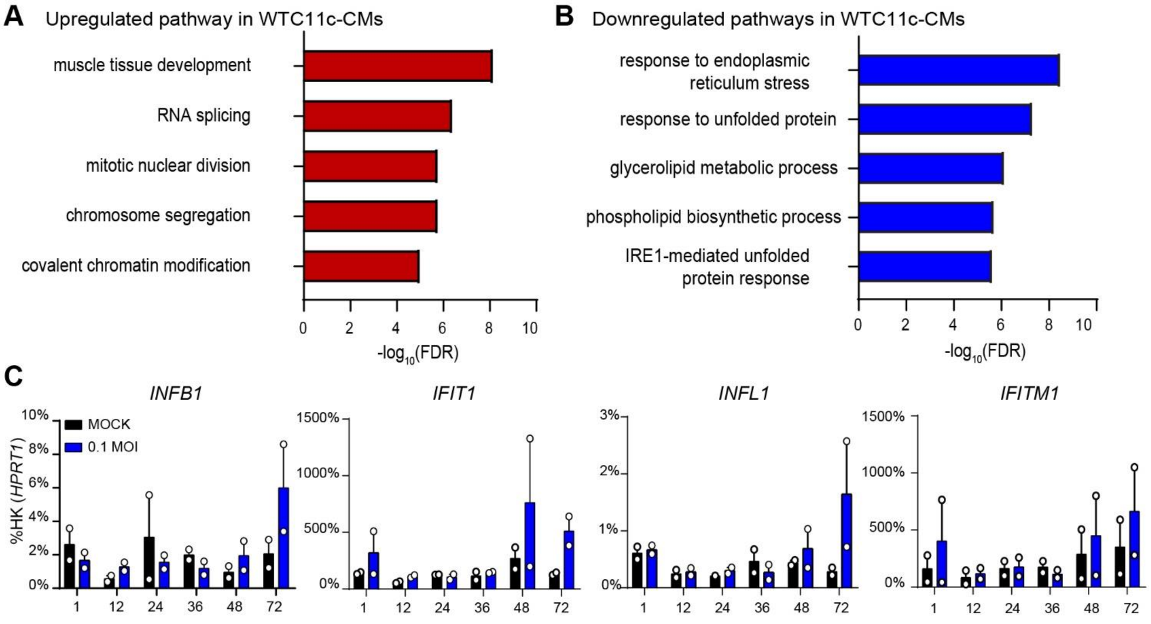


**Supplementary Figure 4 – Gene expression analysis of WTC11c-CMs infected with SARS-CoV-2.** (**A**) GO analysis of upregulated pathways in WTC11c-CMs infected with SARS-CoV-2 at 5 MOI after 48 HPI. (**B**) GO analysis of downregulated pathways in WTC11c-CMs infected with SARS-CoV-2 at 5 MOI after 48 HPI. (**C**) RT-qPCR of interferon response genes in WTC11c hiPSC-CMs infected with SARS-CoV-2 at 0.1 MOI. Mean ± SEM of 2 independent experiments.

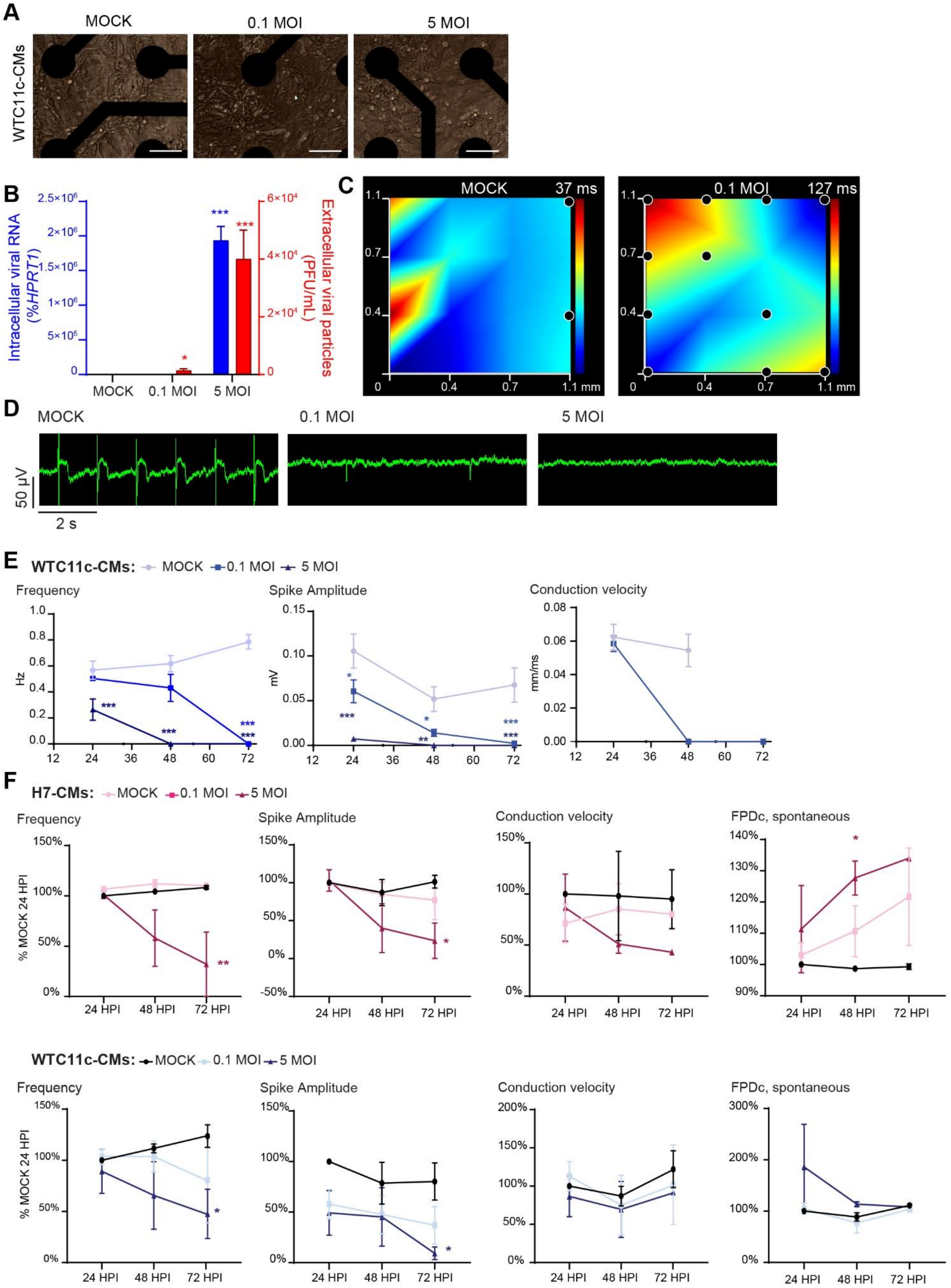

**Supplementary Figure 5 – Electrophysiological alterations in hPSC-CMs infected with SARS-CoV-2.** (**A**) Representative images of SARS-CoV-2-infected WTC11c hiPSC-CMs on MEA well at 72 HPI. Scale bars: 50 µm. (**B**) SARS-CoV-2 viral RNA and extracellular particles detected in WTC11c hiPSC-CMs in MEA plate. Viral RNA is plotted on the right axis as % *HPRT1*, extracellular viral particles are plotted on the left axis as PFU/mL. (**C**) Representative propagation maps of SARS-CoV-2-infected WTC11c hiPSC-CMs at 72 HPI. (**D**) Representative recording of spontaneous electrical activity of SARS-CoV-2-infected WTC11c hiPSC-CMs at 72 HPI. (**E**) Representative quantification of electrophysiological properties from MEA analyses on SARS-CoV-2-infected WTC11c hiPSC-CMs. Mean ± SEM of 8 wells. Differences versus mock calculated by two-way ANOVA with Sidak correction for multiple comparisons (* = $p < 0.05$; ** = $p < 0.01$; *** = $p < 0.001$). (**F**) Aggregated quantification of electrophysiological properties of SARS-CoV-2-infected hPSC-CMs. Data are shown as percentage of MOCK samples at 24 HPI. Mean ± SEM of 3 independent experiments. Statistical analyses as for panel E. Related to Figures 5E, F.

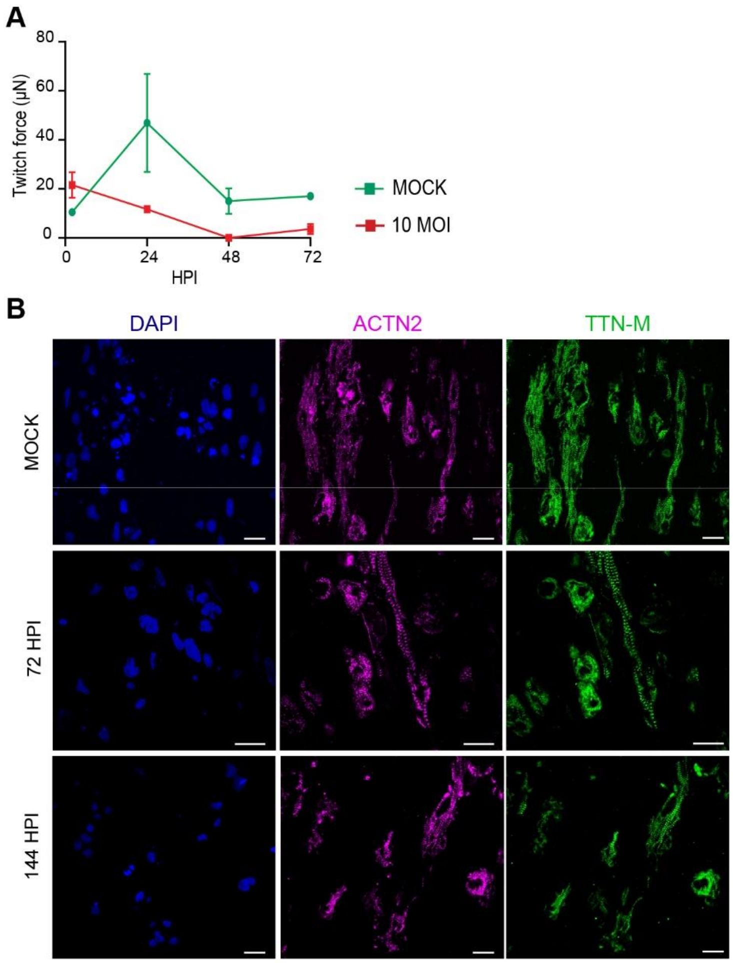


**Supplementary Figure 6 - WTC11c hiPSC-CMs 3D-EHTs infected with SARS-CoV-2.** (**A**) Additional example of a time course analysis of twitch force in 3D-EHTs from WTC11c hiPSC-CMs after SARS-CoV-2 infection at 10 MOI (see Fig. 6D). Data are shown as mean ± SEM for 2 mock controls and 4 infected 3D-EHTs. (**B**) Single channel images for the immunostainings of SARS-CoV-2-infected 3D-EHTs shown in Figure 6F.

## Supplementary Table 1 – Extended data from GO analysis (upregulated pathway for H7-CMs)

| H7-CMs 0.1 MOI at 48 HPI | | | |
|---|---|---|---|
| Ranking | **Pathway** | **FDR** | **$-Log_{10}(FDR)$** |
| **1** | Defense response to virus | 1.76E-07 | 6.75448733 |
| **2** | Mesenchymal cell differentiation | 6.60E-07 | 6.18045606 |
| **3** | Renal system development | 6.75E-07 | 6.17091075 |
| **4** | Kidney development | 7.57E-07 | 6.12101888 |
| **5** | Mesenchyme development | 7.57E-07 | 6.12101888 |
| **6** | Cardiac chamber development | 1.89E-06 | 5.72452411 |
| **7** | Urogenital system development | 1.89E-06 | 5.72452411 |
| **8** | Response to type I interferon | 3.23E-06 | 5.4912459 |
| **9** | Epithelial tube morphogenesis | 3.23E-06 | 5.4912459 |
| **10** | Cardiac septum development | 5.44E-06 | 5.2644011 |
| **11** | Mesenchymal cell development | 5.44E-06 | 5.2644011 |
| **12** | Regulation of cell morphogenesis | 5.87E-06 | 5.23160859 |
| **13** | Type I interferon signaling pathway | 6.91E-06 | 5.16025268 |
| **14** | Cellular response to type I interferon | 6.91E-06 | 5.16025268 |
| **15** | Connective tissue development | 8.28E-06 | 5.08184627 |
| H7-CMs 5 MOI at 48 HPI | | | |
| Ranking | **Pathway** | **FDR** | **$-Log_{10}(FDR)$** |
| ***1*** | *Histone modification* | *1.76E-23* | *22.7544873* |
| **2** | Covalent chromatin modification | 1.14E-22 | 21.941574 |
| ***3*** | *mRNA processing* | *2.85E-18* | *17.5458668* |
| **4** | Peptidyl-lysine modification | 3.30E-15 | 14.4814861 |
| **5** | RNA splicing | 7.22E-15 | 14.1417035 |
| **6** | Regulation of hemopoiesis | 4.55E-12 | 11.3423069 |
| **7** | Regulation of mRNA processing | 3.81E-11 | 10.4186952 |
| ***8*** | *Response to virus* | *3.81E-11* | *10.4186952* |
| **9** | Myeloid cell differentiation | 3.81E-11 | 10.4186952 |
| ***10*** | *Intracellular receptor signaling pathway* | *4.93E-11* | *10.3073293* |
| **11** | Regulation of RNA splicing | 1.44E-10 | 9.84163751 |
| ***12*** | *Defense response to virus* | *1.56E-10* | *9.80773284* |
| **13** | Nuclear export | 1.56E-10 | 9.80773284 |
| **14** | Histone acetylation | 2.95E-10 | 9.52954751 |
| **15** | Mesenchyme development | 3.64E-10 | 9.4392169 |

The pathways in italic are the ones plotted in Fig. 4E.

## Supplementary Table 2 – Extended data from GO analysis (downregulated pathway for H7-CMs)

| H7-CMs 0.1 MOI at 48 HPI | | | |
|---|---|---|---|
| Ranking | **Pathway** | **FDR** | **$-Log_{10}(FDR)$** |
| **1** | ATP synthesis coupled electron transport | 2.64E-27 | 26.57840 |
| **2** | Cellular respiration | 2.64E-27 | 26.57840 |
| **3** | Mitochondrial ATP synthesis coupled electron transport | 2.64E-27 | 26.57840 |
| **4** | Mitochondrion organization | 2.64E-27 | 26.57840 |
| **5** | Respiratory electron transport chain | 4.58E-27 | 26.33913 |
| **6** | Mitochondrial respiratory chain complex assembly | 6.16E-26 | 25.21042 |
| **7** | Oxidative phosphorylation | 1.06E-25 | 24.97469 |
| **8** | mRNA processing | 2.75E-25 | 24.56067 |
| **9** | RNA splicing | 1.86E-23 | 22.73049 |
| **10** | ATP metabolic process | 3.68E-23 | 22.43415 |
| **11** | Electron transport chain | 3.68E-23 | 22.43415 |
| **12** | Ribonucleoprotein complex biogenesis | 4.55E-22 | 21.34199 |
| **13** | Ribonucleoside monophosphate metabolic process | 5.01E-22 | 21.30016 |
| **14** | Purine nucleoside triphosphate metabolic process | 2.07E-21 | 20.68403 |
| **15** | Purine ribonucleoside monophosphate metabolic process | 2.85E-21 | 20.54516 |
| H7-CMs 5 MOI at 48 HPI | | | |
| Ranking | **Pathway** | **FDR** | **$-Log_{10}(FDR)$** |
| ***1*** | *Cellular respiration* | *2.93E-27* | *26.53264* |
| **2** | Mitochondrial respiratory chain complex assembly | 2.93E-27 | 26.53264 |
| **3** | ATP synthesis coupled electron transport | 2.93E-27 | 26.53264 |
| **4** | Mitochondrial ATP synthesis coupled electron transport | 5.06E-27 | 26.29585 |
| ***5*** | *Oxidative phosphorylation* | *8.45E-27* | *26.07325* |
| **6** | Respiratory electron transport chain | 2.20E-26 | 25.65758 |
| **7** | Electron transport chain | 1.63E-25 | 24.78667 |
| ***8*** | *Generation of precursor metabolites and energy* | *2.42E-23* | *22.61618* |
| **9** | NADH dehydrogenase complex assembly | 1.50E-22 | 21.82507 |
| **10** | Mitochondrial respiratory chain complex I assembly | 1.50E-22 | 21.82507 |
| **11** | Energy derivation by oxidation of organic compounds | 8.80E-22 | 21.05552 |
| **12** | Mitochondrial electron transport, NADH to ubiquinone | 6.75E-21 | 20.17091 |
| ***13*** | *Small molecule catabolic process* | *6.77E-20* | *19.16946* |
| ***14*** | *Purine nucleoside triphosphate metabolic process* | *4.02E-19* | *18.39547* |
| **15** | Purine ribonucleotide metabolic process | 8.21E-19 | 18.08548 |

The pathways in italic are the ones plotted in Fig. 4F.

**Supplementary Table 3 – Extended data from GO analysis (upregulated pathway for WTC11c-CMs)**

| WTC11c-CMs 0.1 MOI at 48 HPI | | | |
|---|---|---|---|
| Ranking | **Pathway** | **FDR** | **-Log$_{10}$(FDR)** |
| **1** | RNA splicing | 8.80E-07 | 6.05551733 |
| **2** | Double-strand break repair | 1.06E-05 | 4.97633608 |
| **3** | Sister chromatid segregation | 3.52E-05 | 4.45345734 |
| **4** | mRNA processing | 4.84E-05 | 4.31515464 |
| **5** | RNA splicing, via transesterification reactions | 6.91E-05 | 4.16025268 |
| **6** | RNA splicing, via transesterification reactions with bulged adenosine as nucleophile | 6.91E-05 | 4.16025268 |
| **7** | mRNA splicing, via spliceosome | 6.91E-05 | 4.16025268 |
| **8** | Mitotic nuclear division | 0.000165 | 3.78251606 |
| **9** | Chromosome segregation | 0.00025422 | 3.59478649 |
| **10** | Nuclear division | 0.000264 | 3.57839607 |
| **11** | Mitotic sister chromatid segregation | 0.000264 | 3.57839607 |
| **12** | Organelle fission | 0.00052067 | 3.28344023 |
| **13** | Nuclear chromosome segregation | 0.00052123 | 3.28296995 |
| **14** | Double-strand break repair via homologous recombination | 0.00075429 | 3.12246412 |
| **15** | Recombinational repair | 0.000825 | 3.08354605 |
| WTC11c-CMs 5 MOI at 48 HPI | | | |
| Ranking | **Pathway** | **FDR** | **-Log$_{10}$(FDR)** |
| **1** | Muscle organ development | 2.11E-12 | 11.6753061 |
| ***2*** | *Muscle tissue development* | *7.04E-09* | *8.15242734* |
| **3** | Striated muscle tissue development | 9.68E-09 | 8.01412464 |
| **4** | Sister chromatid segregation | 3.96E-07 | 6.40230481 |
| ***5*** | *RNA splicing* | *3.96E-07* | *6.40230481* |
| **6** | Reproductive system development | 3.96E-07 | 6.40230481 |
| **7** | Reproductive structure development | 8.93E-07 | 6.04935702 |
| ***8*** | *Mitotic nuclear division* | *1.66E-06* | *5.77931092* |
| ***9*** | *Chromosome segregation* | *1.66E-06* | *5.77931092* |
| **10** | Rhythmic process | 1.67E-06 | 5.77676373 |
| **11** | Heart morphogenesis | 5.04E-06 | 5.29756946 |
| **12** | Mitotic sister chromatid segregation | 5.65E-06 | 5.24820785 |
| **13** | Peptidyl-lysine modification | 8.80E-06 | 5.05551733 |
| ***14*** | *Covalent chromatin modification* | *9.97E-06* | *5.00115967* |
| **15** | Nuclear chromosome segregation | 9.97E-06 | 5.00115967 |

The pathways in italic are the ones plotted in Supplementary Fig. 4A.

## Supplementary Table 4 – Extended data from GO analysis (downregulated pathway for WTC11c-CMs)

| WTC11c-CMs 0.1 MOI at 48 HPI | | | |
|---|---|---|---|
| Ranking | **Pathway** | **FDR** | **-Log$_{10}$(FDR)** |
| **1** | Glycerolipid metabolic process | 8.80E-06 | 5.05551733 |
| **2** | Glycoprotein metabolic process | 1.76E-05 | 4.75448733 |
| **3** | Response to endoplasmic reticulum stress | 3.81E-05 | 4.41869523 |
| **4** | Glycerolipid biosynthetic process | 7.54E-05 | 4.12246412 |
| **5** | Glycerophospholipid metabolic process | 7.54E-05 | 4.12246412 |
| **6** | Urogenital system development | 7.54E-05 | 4.12246412 |
| **7** | Protein localization to plasma membrane | 7.54E-05 | 4.12246412 |
| **8** | Glycoprotein biosynthetic process | 0.00012711 | 3.89581648 |
| **9** | Renal system development | 0.00012711 | 3.89581648 |
| **10** | Fat cell differentiation | 0.000184 | 3.73518218 |
| **11** | Kidney development | 0.000184 | 3.73518218 |
| **12** | Phospholipid metabolic process | 0.000352 | 3.45345734 |
| **13** | Membrane lipid metabolic process | 0.00037231 | 3.42909799 |
| **14** | Glycolipid metabolic process | 0.00054686 | 3.26212611 |
| **15** | Liposaccharide metabolic process | 0.00064533 | 3.1902159 |
| WTC11c-CMs 5 MOI at 48 HPI | | | |
| Ranking | **Pathway** | **FDR** | **-Log$_{10}$(FDR)** |
| ***1*** | *Response to endoplasmic reticulum stress* | *3.26E-09* | *8.4873156* |
| ***2*** | *Response to unfolded protein* | *4.84E-08* | *7.31515464* |
| **3** | Response to topologically incorrect protein | 1.03E-07 | 6.98857054 |
| **4** | Small molecule catabolic process | 1.21E-07 | 6.91721463 |
| **5** | Glycerophospholipid metabolic process | 2.99E-07 | 6.52403841 |
| **6** | Lipid modification | 4.25E-07 | 6.37127058 |
| ***7*** | *Glycerolipid metabolic process* | *7.42E-07* | *6.12976336* |
| **8** | Organic acid catabolic process | 9.68E-07 | 6.01412464 |
| **9** | Carboxylic acid catabolic process | 9.68E-07 | 6.01412464 |
| **10** | Glycoprotein metabolic process | 9.68E-07 | 6.01412464 |
| **11** | Protein folding | 1.28E-06 | 5.89279003 |
| **12** | Glycosylation | 1.83E-06 | 5.73675857 |
| ***13*** | *Phospholipid biosynthetic process* | *2.03E-06* | *5.69233943* |
| ***14*** | *IRE1-mediated unfolded protein response* | *2.33E-06* | *5.63344364* |
| **15** | Glycoprotein biosynthetic process | 4.84E-06 | 5.31515464 |

The pathways in italic are the ones plotted in Supplementary Fig. 4B.

## Supplementary Table 5 – RT-qPCR primer sequences used in this study

| Target | Forward primer (5' – 3') | Reverse primer (5' – 3') |
|---|---|---|
| *HPRT1* | TGACACTGGCAAAACAATGCA | GGTCCTTTTCACCAGCAAGCT |
| *NANOG* | TTTGTGGGCCTGAAGAAAACT | AGGGCTGTCCTGAATAAGCAG |
| *TBXT* | CAAATCCTCATCCTCAGTTTG | GTCAGAATAGGTTGGAGAATTG |
| *PDGFRA* | GCTCACTTCACTCTCCCCAAAG | CCGGCGTTCCTGGTCTTAG |
| *TNNT2* | TTCACCAAAGATCTGCTCCTCGCT | TTATTACTGGTGTGGAGTGGGTGTGG |
| *ACE2* | CCATCAGGATGTCCCGGAG | TGGAGGCATAAGGATTTTCTCCA |
| *SARS-CoV-2_E* | GAACCGACGACGACTACTAGC | ATTGCAGCAGTACGCACACA |
| *IFIT1* | AGAAGCAGGCAATCACAGAAAA | CTGAAACCGACCATAGTGGAAAT |
| *IFITM1* | TACTCCGTGAAGTCTAGGGACAG | AACAGGATGAATCCAATGGTCA |
| *IFNB* | CTTGGATTCCTACAAAGAAGCAGC | TCCTCCTTCTGGAACTGCTGCA |
| *IFNL* | AACTGGGAAGGGCTGCCACATT | GGAAGACAGGAGAGCTGCAACT |
| *MYL2* | TTGGGCGAGTGAACGTGAAAA | CCGAACGTAATCAGCCTTCAG |
| *MYH6* | GCCCTTTGACATTCGCACTG | GGTTTCAGCAATGACCTTGCC |

## Supplemental Experimental Procedures

**Cell culture.** Undifferentiated RUES2 hESCs (RUESe002-A; WiCell) and WTC11c hiPSCs (a gift of Dr. Bruce Conklin, Gladstone Institutes, San Francisco) were maintained in mTeSR1 (Stemcell Technologies) on tissue culture dishes coated with Matrigel (Corning) at 0.17 mg/mL, and passaged as small clumps using Versene (Gibco). Cardiomyocytes from RUES2 and WTC11c were differentiated as previously described (Bertero et al., 2019). Briefly, undifferentiated cells were seeded at 1,000 cells/cm$^2$ in mTeSR1 supplemented with 10 µM Y-27632 (Tocris) on Matrigel-coated dishes. After 24 h media was changed to mTeSR1 with 1 µM CHIR-99021 (Cayman). On day 0 mesoderm differentiation was induced with 3 µM CHIR-99021 in RPMI-1640 media (ThermoFisher) supplemented with 500 µg/mL BSA (Sigma-Aldrich) and 213 µg/mL ascorbic acid (Sigma-Aldrich), denoted as RBA media. On day 2 media was changed to RBA containing 2 µM WNTC59 (Selleckchem). On day 4 media was changed to plain RBA. On day 6 media was changed to RPMI-1640 plus B-27 supplement (ThermoFisher), with further media changes every other day. Heat-shock was performed on day 13 for 30 min at 42 °C, and on day 14 cardiomyocytes were dissociated and frozen in CS10 cryopreservation media (Sigma-Aldrich). H7 hESCs (WA07; WiCell) were differentiated in suspension culture format by collaborators at the Center for Applied Technology Development at the City of Hope in California (as previously described (Chen et al., 2015), received by dry-ice shipment, and stored in liquid nitrogen before use.

Undifferentiated H9 hESCs (WA09; WiCell) were maintained and differentiated into LM-SMCs and NC-SMCs as previously described (Bargehr et al., 2016; Serrano et al., 2019). In brief, early mesoderm differentiation was commenced in chemically defined medium with polyvinyl alcohol (CDM-PVA) supplemented with FGF2 (20 ng/mL), LY294002 (10 mM), and BMP4 (10 ng/mL) for 1.5 days. Subsequently, lateral mesoderm differentiation was started in CDM-PVA with FGF2 (20 ng/mL) and BMP4 (50 ng/mL) for 3.5 days. For SMC differentiation, LM cells were resuspended as single cells in CDM-PVA supplemented with PDGF-BB (10 ng/mL) and TGF- β1 (2 ng/mL) for 12 days with media change every other day. To generate NC-SMCs, the NC intermediate was produced by culturing H9 hESCs in CDM-PVA with FGF2 (12 ng/mL) and SB431542 (10 µM; Tocris), which were then split into single cells after 4 days. NC cells were cultured with daily media changes and passaged as needed. After passage 5, SMC induction was initiated with the addition of CDM-PVA with PDGF-BB (10ng/mL) and TGF-β1 (2 ng/mL) for 12 days with media changes every other day. Both LM- and NC-SMCs were subsequently maintained in DMEM F12 (Gibco) supplemented with 10% FBS for 7 days prior to freezing. Following their generation in Cambridge, UK, cells were shipped in liquid nitrogen to Seattle, WA, USA.

**Gene-editing for *ACE2* KO cell line and clonal isolation.** WTC11c hiPSCs were electroporated with CRISPR/Cas9 ribonucleoprotein complexes targeting exon 1 of ACE2 (Liang et al., 2015). Briefly, a mixture of 60 pmol of *Sp*Cas9 2xNLS nuclease (Synthego) and 60 pmol of single guide RNA mix (protospacers 5'-TTTGAACAGGTTTTTACAG-3'; 5'-AATGTCGTTGTTCCGACTCT-3' and 5'-CTGTAAGAGAAGTCATTATA-3'; Synthego) were incubated in 300 µL of Neon Buffer R (ThermoFisher) at room temperature for 1 h. 500,000 WTC11c hiPSCs were resuspended in the ribonucleoprotein mixture, electroporated (1 pulse at 1,300 V for 30 ms), and immediately replated on matrigel-coated 6-well plate in mTeSR1 supplemented with 10 µM Y-27632. Clonal isolation was performed through limiting dilution during passaging. Briefly, the targeted WTC11c hiPSC pool was harvested as single-cell suspension using 0.75X TrypLE in Versene at 37 °C for 3 min, collected with mTeSR1 supplemented with 10 µM Y-27632, serially diluted to reach a density of 5 cell/mL, and seeded onto 96-well plates at a density of 0.5 cell/well. Single cell-derived clones were subsequently maintained and differentiated in cardiomyocytes as described above. Standard G-banding analysis was performed on undifferentiated cells to confirm absence of karyotype abnormalities (Diagnostic Cytogenetics, Seattle WA).

**Genotyping**. Genomic DNA (gDNA) was isolated from *ACE2* KO clones with the DNeasy Blood & Tissue kit (QIAGEN) according to manufacturer's instructions. 500 ng of gDNA was amplified using Q5 High Fidelity 2X Master Mix (NEB) and 5 µM of forward and reverse primers (5'- GCCATAAAGTGACAGGAGAG-3' and 5'-GAAATCCTGACTGTGATGAG-3'). PCR products were purified using the QIAquick PCR purification kit (QIAGEN) and analyzed by Sanger sequencing (sequencing primer: 5'TTTGATTTCCCTTTTCAGTT-3').

**Flow cytometry**. Cardiac differentiation efficiency was determined by flow cytometry in hPSC-CMs fixed with 4% paraformaldehyde for 15 min at room temperature. Cells were centrifuged at 300 g for 5 min and incubated at room temperature for 1 h with either APC-cTnT antibody (Miltenyi Biotech #130-120-543) or APC-IgG1 isotype control antibody (Miltenyi Biotech #130-120-709), both used at 1:100 dilution in DPBS (Gibco) with 5% fetal bovine serum (FBS) and 0.75% saponin. Following washes with DPBS with 5% FBS, samples were run on a BD FACSCanto II flow cytometer and data from 10,000 valid events were acquired with the BD FACSDIVA software. Analysis was performed with FlowJo v10.7.

**RNA-seq.** Bulk RNA-seq datasets from differentiating RUES2 hESC-CMs had been previously generated and analyzed (Bertero et al., 2019) (GEO dataset: GSE106688). Bulk mRNA-seq data from infected cardiomyocytes were generated by constructing mRNA-seq libraries using the KAPA mRNA HyperPrep Kit (Kapa Biosystems) and 100 ng of RNA. Both the RNA and resulting libraries were quality controlled and quantified using a 4200 TapeStation System (Agilent Technologies, Inc.) and a Qubit Fluorometer (Invitrogen). Libraries were sequenced on an Illumina NovaSeq using S2 200 cycle flow cells which generates paired-end reads of 100 nucleotides. Each sample was sequenced to a minimum of 20 million mapped reads. Read quality for each sample was first assessed with FASTQC (Andrews, 2010) and then adapters and bases with phred quality score below 20 were removed from the end of the reads with TrimGalore (Martin, Cutadapt Removes Adapter

Sequences From High-Throughput Sequencing Reads ). Globin and rRNA reads were filtered out using bowtie2 v2.1.0 (Langmead and Salzberg, 2012), and remaining reads were mapped to the hg19 genome using STARv2.7.5a (Dobin et al., 2013). Read counts for each gene were quantified using HTSeqv0.12.4 (Anders et al., 2015). We also mapped reads to the SARS-CoV-2 genome (GCF_009858895.2) using bowtie2v2.1.0 to quantify how many of the reads were derived from virus. Genes with a read count average of less than 10 across all samples were removed. Read counts were normalized using TMM normalization and differentially expressed (DE) genes were determined using the EdgeR and limma Bioconductor packages (Ritchie et al., 2015; Robinson et al., 2010; Robinson, 2010). Significant DE genes had to have an adjusted p-value of less than 0.05 and an absolute fold change greater than 1.5. TopGO R package with Fisher's Exact Test was used for Gene Ontology enrichment analysis (Alexa et al., 2006).

**Western blot.** Cell pellets were incubated in RIPA Buffer supplemented with 1X Protease inhibitors (ThermoFisher) at 4 °C for 20 min. Samples were then centrifuged at 21,000 g for 15 min at 4 °C, and protein concentration in the supernatant was quantified with BCAssay (ThermoFisher). 25 µg of protein were mixed with 1X non-reducing SDS sample buffer and incubated at 37 °C for 30 min. Samples were run on 4-20% mini-PROTEAN TGX precast gels (Bio-Rad) and then transferred on PVDF membranes. Membranes were incubated with 5% non-fat dry milk in TBS buffer supplemented with 0.1% Tween-20 (blocking buffer) for 1 h at room temperature. Primary antibodies were incubated in blocking buffer for 2 h at room temperature (rabbit anti-ACE2 C-terminal domain [Abcam #ab15348, used at 1:500 dilution], rabbit anti-ACE2 N-terminal domain [Novusbio #SN0754, used at 1:500 dilution], mouse anti-cTnT [ThermoFisher #MA5-12960 used at 1:200 dilution], and mouse anti-GAPDH [Abcam #ab8245, used at 1:3,000 dilution]). Membranes were washed and further incubated with fluorescent dye-conjugated secondary antibodies for 1 h at room temperature (AlexaFluor 647 goat anti-rabbit IgG1 and AlexaFluor 488 goat anti-mouse IgG1, both used at 1:1,000 dilution in blocking buffer) and fluorescent signals were acquired using with a GelDoc Imager (Bio-Rad). Quantification of bands intensity was performed using Fiji software.

**Single cell RNA-seq.** A single cell suspension was generated from cultures of RUES2 hESC-CMs at day 30 of differentiation, and single cell RNA-seq was performed using the Chromium NextGEM Single Cell 3' kit (10X Genomics). 10,000 cells per condition were loaded on independent microfluidics channels to generate Gel bead-in-Emulsion (GEMs), which were further processed according to the manufacturer's instructions to generate Illumina-compatible sequencing libraries. The sample was analyzed using two runs of high output NextSeq 500 with a 150 cycle kit, reading 28 base pairs for read 1 (barcode and UMI), 91 base pairs for read 2 (3' end of cDNAs), and 7 base pairs for the i7 index. Initial data analysis relied on the cellranger package from 10X Genomics. Cellranger mkfastq was used to transfer demultiplexed raw base call files into library-specific FASTQ files. The FASTQ files were separately mapped to the GRC38 human reference genome using STAR as a part of the cellranger pipeline. Gene expression counts were done using cellranger count based on Gencode v25 annotation, and cell identifiers and Unique Molecular Identifiers (UMI) were filtered and corrected with default setting. Raw cellranger count outputs was aggregated and visualized by a subsampling procedure using cellranger aggr. Downstream analysis was performed in the R package Seurat (Butler et al., 2018; Stuart et al., 2019). Filters were applied to eliminate cells with less than 1,000 genes detected, with over 40,000 UMIs, or with over 35% mitochondrial gene reads. Post filtering, cell-to-cell gene expression was normalized by total expression, multiplied by the scale factor of 10000, and the result is log-transformed [Log norm exp]). Data dimensionality reduction was done by principal component analysis on the top 2,000 most variable genes. The top 10 principal components (PCs) that explained most variance were selected, as confirmed by an Elbow plot. For visualization, UMAP dimensionality reduction for the top 10 PCs was performed to produce coordinates for cells in 2 dimensional space. The relative expression levels of genes of interest were plotted using the FeaturePlot function in Seurat.

**SARS-CoV-2 generation.** All experiments using live virus were performed in the Biosafety Level 3 (BSL-3) facility at the University of Washington in compliance with the BSL-3 laboratory safety protocols (CDC BMBL 5$^{th}$ ed.) and the recent CDC guidelines for handling SARS-CoV-2. Before removing samples from BSL-3 containment, samples were inactivated by Trizol or 4% paraformaldehyde, and the absence of viable SARS-CoV-2 was confirmed for each sample by plaque assays. SARS-Related Coronavirus 2, Isolate USA-WA1/2020 (SARS-CoV-2) and icSARS-CoV-2mNG were obtained from BEI Resources (NR-52281) and the University of Texas (Xie et al., 2020), respectively and propagated in VERO cells (USAMRIID). Briefly, VERO cells were maintained in DMEM (Gibco) supplemented with 10% heat-inactivated FBS, 100 U/mL penicillin, and 100 U/mL streptomycin at 37 °C in a 5% $CO_2$ humidified incubator. To generate virus stock, cells were washed once with DPBS and infected with SARS-CoV-2 in serum-free DMEM. After 1 h of virus adsorption, the inoculum was replaced with DMEM supplemented with 2% heat-inactivated FBS, and cells were incubated at 37 °C in a 5% $CO_2$ incubator until ~70% of cells manifested cytopathic effects. The virus was harvested by collecting the culture supernatant followed by centrifugation at 3,000 g for 15 min at 4 °C to remove the cell debris. Virus titer was then measured by plaque assay on VERO cells (as described below), and stocks were stored at -80°C.

**SARS-CoV-2 titering.** Viral preparations and culture supernatant from SARS-CoV-2-infected cardiomyocytes were titered using a plaque assay. Briefly, 350,000 VERO cells were seeded in 12-well plates and incubated for 1 h at 37 °C with 10-fold dilutions of virus-containing media. A solution of 1:1 1% agarose and 2X DMEM supplemented with 4% heat-inactivated FBS, L-glutamine, 1X antibiotic-antimycotic (Gibco), and 220 mg/mL sodium pyruvate was layered on top of the cells, followed by incubation at 37 °C for 2 days. After fixing with 10% formaldehyde, the agarose layer was removed and cells

were stained with 0.5% crystal violet solution in 20% ethanol. Plaques were counted, and the virus titer in the original sample was assessed as plaque-formation unit per mL (PFU/mL).

**Viral infection.** Cryopreserved hPSC-CMs were thawed and plated in RPMI-1640 supplemented with B-27, 5% FBS, and 10 µM Y-27632. After 24 h the media was replaced with RPMI-1640 supplemented with B-27 only. After 3 days, cardiomyocytes were harvested with Versene supplemented with 0.5% Trypsin (Gibco) at 37 °C for 5 min to obtain single-cell suspensions. 250,000 cardiomyocytes were seeded in Matrigel-coated 12-well plates in RPMI-1640 supplemented with B-27, 5% FBS, and 10 µM Y-27632. The media was replaced with RPMI-1640 supplemented with B-27 the next day, and then every other day for 1 week. SARS-CoV-2 wild-type or expressing Neon green protein was diluted to the desired MOI in DMEM and incubated on hPSC-CMs for 1 h at 37 °C (non-infected [mock] controls were incubated with DMEM only). Cells were then washed with DPBS and cultured in RPMI-1640 supplemented with B-27. Cryopreserved hPSC-SMCs were thawed in DMEM F12 supplemented with 10% FBS and 10 µM Y-27632. After 24 h, the media was replaced with DMEM F12 supplemented with 10% FBS only. After 4 days, smooth muscle cells were harvested with 0.5% Trypsin (Gibco) at 37 °C for 5 min to obtain single-cell suspensions. 100,000 smooth muscles cells were seeded in gelatin-coated 12-well plates in DMEM F12 supplemented with 10% FBS and 10 µM Y-27632. The media was replaced after 24 h with fresh DMEM F12 with 10% FBS. After 5 days from replating, hPSC-SMCs were incubated with SARS-CoV-2 diluted to the desired MOI in DMEM for 1 h at 37 °C (non-infected [mock] controls were incubated with DMEM only). Cells were then washed with DPBS and cultured in DMEM F12 supplemented with 10% FBS.

**Immunofluorescence.** 200,000 cardiomyocytes were plated on glass-bottom 24-well plate (CellVis) and infected as described above. Cells were fixed with 4% paraformaldehyde in DPBS for 30 min at room temperature and then washed 3 times with DPBS for 5 min. Cells were permeabilized using 0.25% Triton X-100 (Sigma-Aldrich) in DPBS and blocked for 1 h with 10% normal goat serum supplemented with 0.1% Tween-20 in DPBS. Primary antibodies were incubated overnight at 4 °C in DPBS with 1% normal goat serum and 0.1% Tween-20 (rabbit anti-2019-nCoV NP [Sino Biological #40143-R019, used at 1:200 dilution], and mouse anti-Sarcomeric α-actinin [Abcam ab# ab9465, used at 1:500 dilution]). Cell were washed three times with DPBS containing 0.2% Tween-20, and incubated for 1 h at room temperature with secondary antibodies diluted in DPBS supplemented with 1% BSA and 0.1% Tween-20 (AlexaFluor 594 goat anti-rabbit IgG1 and AlexaFluor 647 goat anti-mouse IgG1, both used at 1:1,000 dilution). DAPI (Sigma-Aldrich) was diluted at 300 nM in water and incubated on the cells for 15 min at room temperature, followed by three washes in DPBS containing 0.2% Tween-20. Images were taken with a 40x oil objective on a Nikon Eclipse microscope with Yokogawa W1 spinning disk head, and formatted with Fiji software.

**Electron Microscopy.** 200,000 cardiomyocytes were plated on 35-mm petri dish (Corning) and infected as described above. Cells were fixed with Karnovsky's fixative for 30 min at room temperature and then washed 3 times with 0.1 M PIPES buffer, for 5 min. Heavy metal impregnation was performed as detailed elsewhere (Deerinck, 2010). The embedded cellular layer was cut into small pieces and re-embedded in en-face and perpendicular orientations in Durcopan (EMS). Thin sections were viewed using a JEOL 1230 transmission electron microscope.

**Gene expression analysis and viral RNA detection.** Infected cardiomyocytes were washed once with DPBS and incubated with 400 µL per well of Trizol reagent (Invitrogen) for 10 min at room temperature. Chloroform was added in a 5:1 ratio to Trizol, and samples were incubated at room temperature for 2 min. The aqueous phase was separated by centrifugation (21,000 g for 15 min at 4 °C) and incubated with isopropanol (1:1 ratio) and 25 µg of Glycoblue (ThermoFisher) for 10 min at room temperature. RNA pellets were harvested by centrifugation (21,000 g for 15 min at 4 °C), washed twice with 75% ethanol, and resuspended in nuclease-free water. cDNA was prepared with M-MLV reverse transcriptase according to the manufacturers' instruction. Quantitative real-time reverse transcription PCR (RT-qPCR) was performed with SYBR Select Master Mix (Applied Biosystems) using 10 ng of cDNA and 400 nM forward and reverse primers (Supplementary Table 5). Reactions were run on a CFX384 Real-Time System (Bio-Rad), and data was analyzed using the ΔΔCt method using *HPRT1* as the housekeeping gene. Primers were designed using PrimerBlast, and confirmed to amplify a single product.

**Electrophysiological analysis with MEA.** Cryopreserved cardiomyocytes were thawed and cultured as described above. CytoView MEA 48- and 24-well plates (Axion BioSystems) were coated with 0.17 mg/mL of Matrigel for 1 h at 37 °C. 50,000 (48-well plate) or 100,000 (24-well plate) hPSC-CMs were resuspended in 6 µL or 10 µL, respectively, and plated on each MEA well, as previously described (Hayes et al., 2019). Media was changed with RPMI-1640 supplemented with B-27 every other day for 1 week. One the day of viral infection, cells were washed once with DPBS and incubated with 50 µL (48-well plate) or 100 µL (24-well plate) of SARS-CoV-2 suspension for 1 h at 37 °C. Media was replaced with RPMI-1640 supplemented with B-27, and the plate was transferred directly into Maestro Pro system (Axion BioSystems) and kept at 37 °C with 5% $CO_2$ for the duration of the experiment. Electrophysiological recordings were taken for 5 min at specified time points using Axis software version 2.0.4. (Axion BioSystems). Voltage was acquired simultaneously for all the electrodes at 12.5 kHz, with a low-pass digital filter of 2 kHz for noise reduction. The beat detection threshold was 100 µV, and the FPD detection used a polynomial regression algorithm with the threshold set at 1.5 × noise to detect repolarization waves. Pacing was performed at 2 Hz with an alternating square wave (± 1 V, 100 nA, 8.33 kHz) delivered through the dedicated stimulator in the Maestro Pro system to a selected electrode (not used for recording). Automated analysis was performed using Cardiac

Analysis Software v3.1.8 (Axion BioSystems), which automatically computes the Fridericia correction to account for beat rate variability during FPD measurements [$FPDc = FPD/(\text{beat period})^{1/3}$].

**Contractility analysis with 3D-EHTs.** 3D-EHTs were generated from hPSC-CMs embedded with stromal cells in a 3D fibrin gel suspended between pairs of silicone posts, as previously described (Bielawski et al., 2016). For each pair of silicone posts one was flexible and had a 1 $mm^3$ magnet embedded in its tip, and the other post was rendered rigid by embedding a 1.1 mm glass capillary tube. Each 3D-EHT was casted in a mold made of 2% agarose by adding 500,000 WTC11c hiPSC-CMs and 50,000 HS27a stromal cells (ATCC) in a fibrin gel solution (89 µL RPMI-1640 supplemented with B-27, 5.5 µl of DMEM/F12, [Gibco], 2.5 µL of 200 mg/mL bovine fibrinogen [Sigma-Aldrich], and 3 µL of 100 U/mL thrombin [Sigma-Aldrich]). The cell-gel mixture was incubated at 37 °C for 2 h to allow for fibrin polymerization. Afterwards, 3D-EHTs were transferred from the agarose molds to 24-well tissue culture dishes containing 3D-EHT media (RPMI-1640 media supplemented with B-27 and 5 mg/mL aminocaproic acid [Sigma-Aldrich]. Media was changed every other day for 2 weeks. For SARS-CoV-2 infection, 3D-EHTs were temporarily housed in 2% agarose molds, and 200 µL of viral solution in DMEM was used to infect each single tissue for 1 h at 37 °C (DMEM was used for mock controls). 3D-EHTs were then transferred in fresh 3D-EHT media for the rest of the experiment. Twitch force was recorded by tracking the movement of magnets embedded in the flexible posts, as previously described (Bielawski et al., 2016). Briefly, we used a custom-built printed circuit board (PCB) containing giant magnetoresistive (GMR) sensors (NVE, Eden Prairie, MN) in a Wheatstone bridge configuration and relying on instrumentation amplifiers and operational amplifiers to filter out signal noise. 3D-EHTs in the 24-well dish were placed into a 3D-printed caddy that contained the PCB with GMR sensors such that the flexible, magnetic posts of 3D-EHTs were directly above each GMR sensor. Data from the PCB was collected by LabView (National Instruments) on a laptop in the BSL-3 facility. The voltage traces from the magnetic sensors were analyzed for amplitude and frequency using a custom Matlab protocol. The amplitudes were then converted from voltage to twitch force using a characterization constant.

**3D-EHTs immunostaining.** At the indicated time points, 3D-EHTs were harvested for immunofluorescence. 3D-EHTs were treated with 150 mM KCl to arrest contraction in diastole, and fixed in 4% paraformaldehyde for 30 min at room temperature. 3D-EHTs were dehydrated with 30% sucrose overnight before embedding in TissueTek O.C.T. compound (VWR). Cryoblocks were sectioned at a 5 µm thickness. Slides housing tissue sections were blocked and permeabilized with 1% Bovine Serum Albumin (Sigma-Aldrich) and 0.1% Triton-X-100 (Sigma-Aldrich) for 1 h at room temperature. Primary antibodies were diluted in the blocking buffer and incubated overnight at 4 °C (mouse anti-Sarcomeric α-actinin [Abcam ab# ab9465, used at 1:200 dilution], and mouse anti-Titin M-line [Myomedix #M8M10, used at 1:200 dilution]). After three washes with DPBS (5min/each), slides were incubated for 1 h at room temperature with secondary antibodies diluted in blocking buffer (AlexaFluor 488 goat anti-rabbit IgG1 and AlexaFluor 647 goat anti-mouse IgG1, both used at 1:500 dilution). Tissue slides were mounted with coverslips using Vectashield mounting media with DAPI (Vector). Images were taken with a 60x oil objective on a Nikon Ti microscope and formatted with Fiji software.

## Supplemental References


Alexa, A., Rahnenfuhrer, J., and Lengauer, T. (2006). Improved scoring of functional groups from gene expression data by decorrelating GO graph structure. Bioinformatics *22*, 1600-1607.

Anders, S., Pyl, P.T., and Huber, W. (2015). HTSeq--a Python framework to work with high-throughput sequencing data. Bioinformatics *31*, 166-169.

Andrews, S. (2010). FastQC: a quality control tool for high throughput sequence data.

Bielawski, K.S., Leonard, A., Bhandari, S., Murry, C.E., and Sniadecki, N.J. (2016). Real-Time Force and Frequency Analysis of Engineered Human Heart Tissue Derived from Induced Pluripotent Stem Cells Using Magnetic Sensing. Tissue Eng Part C Methods *22*, 932-940.

Butler, A., Hoffman, P., Smibert, P., Papalexi, E., and Satija, R. (2018). Integrating single-cell transcriptomic data across different conditions, technologies, and species. Nat Biotechnol *36*, 411-420.

Chen, V.C., Ye, J., Shukla, P., Hua, G., Chen, D., Lin, Z., Liu, J.C., Chai, J., Gold, J., Wu, J.*, et al.* (2015). Development of a scalable suspension culture for cardiac differentiation from human pluripotent stem cells. Stem Cell Res *15*, 365-375.

Dobin, A., Davis, C.A., Schlesinger, F., Drenkow, J., Zaleski, C., Jha, S., Batut, P., Chaisson, M., and Gingeras, T.R. (2013). STAR: ultrafast universal RNA-seq aligner. Bioinformatics *29*, 15-21.

Langmead, B., and Salzberg, S.L. (2012). Fast gapped-read alignment with Bowtie 2. Nat Methods *9*, 357-359.

Liang, X., Potter, J., Kumar, S., Zou, Y., Quintanilla, R., Sridharan, M., Carte, J., Chen, W., Roark, N., Ranganathan, S.*, et al.* (2015). Rapid and highly efficient mammalian cell engineering via Cas9 protein transfection. J Biotechnol *208*, 44-53.

Martin, M. (Cutadapt Removes Adapter Sequences From High-Throughput Sequencing Reads ). Cutadapt Removes Adapter Sequences From High-Throughput Sequencing Reads EMBnet.

Ritchie, M.E., Phipson, B., Wu, D., Hu, Y., Law, C.W., Shi, W., and Smyth, G.K. (2015). limma powers differential expression analyses for RNA-sequencing and microarray studies. Nucleic Acids Res *43*, e47.

Robinson, M.D., McCarthy, D.J., and Smyth, G.K. (2010). edgeR: a Bioconductor package for differential expression analysis of digital gene expression data. Bioinformatics *26*, 139-140.

Robinson, M.D., Oshlack, A. (2010). A scaling normalization method for differential expression analysis of RNA-seq data. Genome Biol *11*.

Stuart, T., Butler, A., Hoffman, P., Hafemeister, C., Papalexi, E., Mauck, W.M., 3rd, Hao, Y., Stoeckius, M., Smibert, P., and Satija, R. (2019). Comprehensive Integration of Single-Cell Data. Cell *177*, 1888-1902 e1821.
